# Detecting and quantifying liquid–liquid phase separation in living cells by model-free calibrated half-bleaching

**DOI:** 10.1038/s41467-022-35430-y

**Published:** 2022-12-16

**Authors:** Fernando Muzzopappa, Johan Hummert, Michela Anfossi, Stanimir Asenov Tashev, Dirk-Peter Herten, Fabian Erdel

**Affiliations:** 1 Molecular, Cellular and Developmental Biology Unit (MCD), Center for Integrative Biology (CBI), CNRS, UPS, Toulouse, France; 2 College of Medical and Dental Sciences & School of Chemistry, University of Birmingham, Birmingham, UK; 3 Centre of Membrane Proteins and Receptors (COMPARE), Universities of Birmingham and Nottingham, Birmingham, UK

**Keywords:** Permeation and transport, Confocal microscopy, Imaging and sensing, Intrinsically disordered proteins

## Abstract

Cells contain numerous substructures that have been proposed to form via liquid–liquid phase separation (LLPS). It is currently debated how to reliably distinguish LLPS from other mechanisms. Here, we benchmark different methods using well-controlled model systems in vitro and in living cells. We find that 1,6-hexanediol treatment and classical FRAP fail to distinguish LLPS from the alternative scenario of molecules binding to spatially clustered binding sites without phase-separating. In contrast, the preferential internal mixing seen in half-bleach experiments robustly distinguishes both mechanisms. We introduce a workflow termed model-free calibrated half-FRAP (MOCHA-FRAP) to probe the barrier at the condensate interface that is responsible for preferential internal mixing. We use it to study components of heterochromatin foci, nucleoli, stress granules and nuage granules, and show that the strength of the interfacial barrier increases in this order. We anticipate that MOCHA-FRAP will help uncover the mechanistic basis of biomolecular condensates in living cells.

## Abstract (web summary)

It is currently debated how to reliably distinguish liquid–liquid phase separation (LLPS) from other mechanisms. Here the authors report model-free calibrated half-FRAP (MOCHA-FRAP) to probe the barrier at the condensate interface that is responsible for preferential internal mixing in LLPS.

## Introduction

**Fig. 1:**
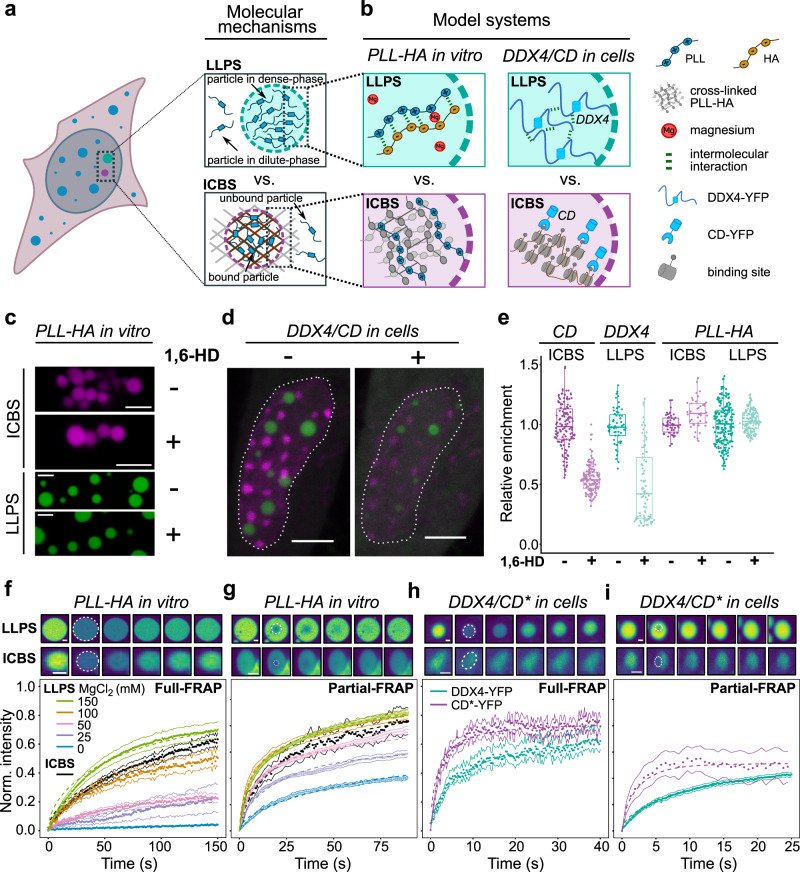
Benchmarking 1,6-hexanediol treatment and classical FRAP using well-defined model systems. **a** Schematic representation of membrane-less structures formed by LLPS (top) or ICBS (bottom). **b** Schematic representation of the model systems used in this work: PLL-HA coacervates undergoing LLPS in vitro (top, left), PLL undergoing ICBS in vitro (bottom, left), DDX4-YFP undergoing LLPS in living cells (top, right) and the chromodomain of CBX1/HP1β undergoing ICBS at pericentric heterochromatin enriched for H3K9me2/3 (bottom, right). **c**, Microscopy images of PLL-HA coacervates (green) and PLL undergoing ICBS (magenta) in the absence or presence of 1,6-hexanediol. Scale bars, 5 µm. **d** Microscopy images of a living cell expressing DDX4-YFP and CD-mKate, in the absence or presence of 1,6-hexanediol. Scale bars, 5 µm. **e** Quantification of PLL-FITC, DDX4-YFP and CD-mKate enrichment at structures of interest. Error bars represent the standard deviation. **f** Full-FRAP experiments of PLL-HA coacervates undergoing LLPS, in the absence or presence of different amounts of magnesium chloride (colored curves), and PLL undergoing ICBS (black curve). Top panels show representative snapshots before the bleach and 0, 5, 15, 50 and 75 s after the bleach. The snapshots for LLPS correspond to PLL-HA with 150 mM MgCl_2_. Scale bars, 2 µm. **g** Same as panel f but for partial-FRAP. **h** Same as panel f but for DDX4-YFP (cyan curve) and CD*-YFP (magenta curve) in living cells. **i** Same as panel h but for partial-FRAP. Error bars in panels **f**–**i** represent the standard error of the mean. Source data are provided as a Source Data file.

Phase separation has recently received tremendous interest as a putative organizing principle behind cellular compartmentalization^1^. Many proteins can undergo LLPS in the test tube, and many cellular substructures that are dynamic and appear as puncta under the microscope have been proposed to form via LLPS. However, recent work has suggested that some of these substructures are formed by different mechanisms^2–7^. An alternative model for structures that are associated with a very long polymeric scaffold, like a segment of a chromosome, is that they arise from proteins undergoing low-valency interactions with spatially clustered binding sites (ICBS) on the scaffold^8^, without undergoing LLPS (Fig. 1a). This model applies for example to proteins that have only one or two binding sites to interact with the scaffold and do not interact with one another. It also applies to proteins that can interact with one another and can in principle undergo LLPS but do not do so because their concentration is too low. Hence, ICBS describes scenarios in which proteins do not phase-separate because they do not establish enough multivalent interactions that are required for LLPS^1,9^. An extended discussion of both models is provided in Supplementary Note 1. Depending on the properties of the proteins, both ICBS and LLPS may go along with bridging-induced polymer–polymer phase separation (PPPS/BIPS) that reorganizes the polymeric scaffold into separated domains^3,10,11^. Substructures formed by ICBS or LLPS differ from each other although they also share some similarities and may appear as puncta under the microscope^2,3,8^. One of the arguably most interesting hallmarks of LLPS is the presence of a separated pool of proteins that preferentially move within the substructure, resulting in a locally increased concentration of mobile proteins. In the ICBS model, there is no such separation of protein pools, and the concentration of mobile proteins, which are available for binding reactions, is the same inside and outside of the substructure^8^ (see Supplementary Note 1). Despite our rapidly increasing knowledge about dynamic cellular structures and their constituents, it is currently debated how to identify structures formed by LLPS in living cells and how to distinguish them from those formed by other mechanisms such as ICBS^2,7,12,13^. Besides their morphology and ability to fuse, their resistance to the aliphatic alcohol 1,6-hexanediol (1,6-HD) and the turnover of their components seen in fluorescence recovery after photobleaching (FRAP) experiments have been assessed to infer if they might be formed by LLPS. However, we currently lack a side-by-side comparison of these assays performed on different types of structures, and it is, therefore, unclear how the different readouts are related to the underlying mechanisms.

Here, we address this issue by benchmarking different methods using well-controllable systems that serve as models for LLPS and ICBS in vitro and in living cells (Fig. 1b and Supplementary Fig. 1). On the one hand, as in vitro models, we use coacervates composed of FITC-labeled Poly-L-Lysine (PLL) and Hyaluronic Acid (HA), a well-characterized LLPS system whose material properties can be tuned by addition of salt^14,15^. To mimic ICBS, we use FITC-labeled PLL interacting with chemically crosslinked PLL-HA coacervates that serve as immobile binding site clusters (Supplementary Fig. 2). On the other hand, as live-cell models, we use mouse fibroblasts expressing DDX4-YFP, which forms liquid-like nuage granules that are partitioned from chromatin^16^, and different versions of the chromodomain (CD) of CBX1/HP1β^17,18^, a well-characterized low-valency binder of histone H3 lysine 9 di/trimethylation (H3K9me2/3) that localizes in clusters at pericentric heterochromatin repeats. Our results indicate that 1,6-HD treatment can retrieve information about the nature of the molecular interactions within a substructure but does not distinguish between LLPS and ICBS. Likewise, full- or partial-FRAP experiments can yield very similar recovery curves for molecules undergoing LLPS or ICBS, and can, therefore, not be used to reliably identify the underlying mechanism. However, we find that half-FRAP experiments can distinguish between LLPS and ICBS, as the fluorescence signal in the non-bleached half shows a more pronounced dip in the case of LLPS. We show in molecular dynamics simulations that the depth of this dip scales with the strength of the attractive intermolecular interactions, which are responsible for the occurrence of LLPS and the formation of an interfacial barrier that restricts the exchange of molecules between the phases. By conducting half-FRAP on a panel of LLPS systems, we determine the relationship between the dip depth and the interfacial energy per molecule. Based on these results, we establish a model-free calibrated half-FRAP (MOCHA-FRAP) workflow that can be used to detect LLPS and to quantify the interfacial properties of biomolecular condensates in living cells.

## Results and discussion

### Hexanediol treatment probes the chemical nature of interactions rather than the presence of LLPS

We set out to benchmark different methods that are currently used to study biomolecular condensates in living cells. We first assessed the effect of 1,6-hexanediol (1,6-HD), an aliphatic alcohol that perturbs weak hydrophobic interactions and that is commonly used to probe structures suspected to be formed by LLPS^19^. We added 1,6-HD to PLL-HA coacervates undergoing LLPS, and to PLL undergoing ICBS. Both types of structures were resistant to 1,6-HD treatment (Fig. 1c), indicating that PLL and HA primarily interact via electrostatic interactions that are insensitive to 1,6-HD, regardless if molecules undergo LLPS or ICBS. Treatment of cells expressing DDX4-YFP and CD-mKate with 1,6-HD led to both decreased enrichment of DDX4 in nuage granules and decreased enrichment of CD at heterochromatin foci (Fig. 1d, e), suggesting that both DDX4 self-interactions within liquid-like nuage granules and binding interactions between CD and H3K9me2/3 involve hydrophobic interactions that are sensitive to 1,6-HD. Accordingly, we conclude that 1,6-HD treatment probes the presence of hydrophobic interactions rather than distinguishing between the underlying mechanisms LLPS and ICBS.

### Classical FRAP assays probe the dynamics of interactions rather than the presence of LLPS

Next, we benchmarked the FRAP assay using the model systems introduced above. In classical FRAP experiments, photobleaching is performed on an entire structure (full-FRAP) or a part of it (partial-FRAP), after which the fluorescence recovery in the bleach region is measured^8,20^. For ICBS, the recovery depends on the viscosity and the binding rate constants, while for LLPS, the recovery depends on the viscosities of the individual phases and, particularly in the case of full-FRAP, on the molecular exchange across the interface that separates the phases. For PLL-HA coacervates in the absence of salt, the slow recovery in full-FRAP and the fast recovery in partial-FRAP (Fig. 1f, g, blue curves) indicate that internal mixing within the coacervates is fast but exchange across their interface is slow. Increasing salt concentrations led to faster full- and partial-FRAP recoveries, suggesting that both internal mixing and exchange across the interface become faster when interactions between PLL and HA are screened (Fig. 1f, g, colored curves). Full- and partial-FRAP of PLL undergoing ICBS yielded recovery curves that resembled those of coacervates at intermediate salt concentrations (Fig. 1f, g, black curves), reflecting turnover of PLL molecules at binding site clusters. Likewise, full- and partial-FRAP in cells expressing DDX4-YFP or an engineered CD-YFP version that binds tightly to H3K9me3^17^, which we refer to as CD*-YFP, yielded recovery curves that were very similar to each other and could be fitted with the same kinetic model (Fig. 1h, i). Accordingly, recoveries in full- or partial-FRAP experiments can vary among condensates formed by LLPS under different conditions, in agreement with the broad range of rates reported for different LLPS systems^2^. Importantly, full- or partial-FRAP cannot distinguish between LLPS and ICBS.

### Half-FRAP can distinguish LLPS from the alternative ICBS model in vitro and in living cells

**Fig. 2:**
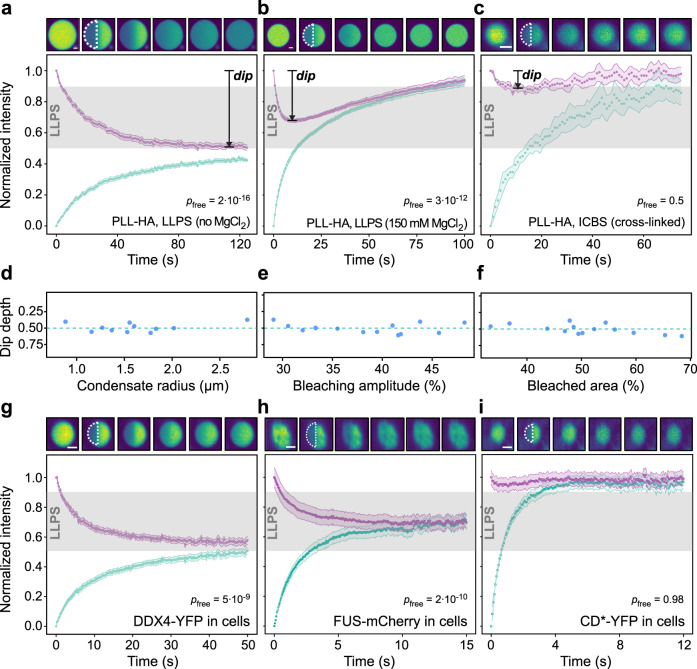
Half-FRAP differentiates condensates formed by LLPS from clusters formed by ICBS. **a**–**c** Half-FRAP curves, including the normalized intensity in the bleached half (cyan) and the non-bleached half (magenta). Gray areas in the plots indicate the range of dip depths in the non-bleached half that correspond to LLPS. Half-FRAP curves for PLL-HA coacervates without magnesium chloride (**a**), PLL-HA coacervates with 150 mM magnesium chloride (**b**) and PLL undergoing ICBS (**c**) are shown. Top panels show representative snapshots before the bleach and 0, 5 15, 50, and 75 s after the bleach. *p*-values based on a one-sided Student’s *t*-test against the dip depths obtained for free diffusion are indicated. **d** Dip depths measured for half-bleached PLL-HA coacervates of different sizes. **e** Dip depths measured for PLL-HA coacervates that were half-bleached with different laser powers. **f** Dip depths measured for PLL-HA coacervates after bleaching different area fractions of them. Half-FRAP in living cells expressing DDX4-YFP (**g**), FUS-mCherry (**h**) or CD*-YFP (**i**). Top panels show representative snapshots before the bleach and 0, 5, 15, 50, and 75 s after the bleach. Scale bars, 2 µm. Error bands in **a**–**c** and **g**–**i** represent the standard error of the mean. *p*-values based on a one-sided Student’s *t*-test against the dip depths obtained for free diffusion are indicated in a-c and g-i. Source data are provided as a Source Data file.

A special case of partial-FRAP, in which half of the structure of interest is bleached, has been introduced to probe the internal dynamics of cellular condensates^21^. However, these types of experiments have rarely been quantified, and it is currently not clear how the resulting recovery curves are related to the underlying molecular interactions. While the recovery of the bleached half alone cannot distinguish between LLPS and ICBS as shown for the conceptually similar partial-FRAP experiments above, we sought to assess how reliably the joint analysis of the fluorescence signals in both halves can distinguish both mechanisms. First, we carried out half-FRAP experiments using PLL-HA coacervates in the absence of salt. We observed an increase of fluorescence in the bleached half and a concomitant decrease in the non-bleached half (Fig. 2a). This signature reflects preferential internal mixing, during which molecules exchange between both halves of the coacervate without crossing the interface between the coacervate and the surrounding phase. The fluorescence in the non-bleached half decreased to half its initial value, meaning that the interior of the coacervate got nearly completely mixed while no significant exchange with the surrounding phase occurred. We refer to the maximum intensity decrease of the non-bleached half as “dip depth”, which can be determined in a model-free manner from the normalized and smoothed half-FRAP curves (see *Half-FRAP data analysis* in the “Methods” section for details). Large dip depths reflect a strong asymmetry of the recovery process, i.e., a large preference of bleached molecules to move into the non-bleached half rather than moving across the interface on the opposite side into the surrounding phase.

We next performed half-FRAP of PLL-HA coacervates in the presence of salt, which partially screens the interactions between PLL and HA. We found that the dip depth gradually decreased with increasing salt concentrations, while curves returned to their initial values more rapidly (Fig. 2b and Supplementary Fig. 3a). Accordingly, increasing salt concentrations led to an increasing exchange of molecules with the surrounding phase, reducing the relative contribution of the recovery coming from the non-bleached half of the coacervate. To determine the dip depth for free diffusion in the 1-phase regime, we conducted half-FRAP experiments in a solution of freely diffusing PLL (Supplementary Fig. 3b). These experiments yielded a dip depth of 10 ± 3%, corresponding to the level of internal mixing obtained in the absence of any interface that could hinder molecular exchange. Notably, the dip depths obtained for PLL-HA undergoing LLPS at all salt concentration were significantly larger than this value (Supplementary Fig. 3a–c, *p* < 10^−11^). In contrast, half-FRAP of PLL undergoing ICBS showed that the bleached half recovered its fluorescence while the dip depth in the non-bleached half was very small (Fig. 2c) and was not significantly larger than the dip depth observed for freely diffusing PLL (*p* = 0.5). When conducting half-FRAP experiments with other in vitro LLPS systems, including PEG-Rhodamine/dextran, DDX4-YFP/PEG (Supplementary Fig. 4) and GFP-HP1α/PEG (Supplementary Fig. 5a, b), we also observed preferential internal mixing with dip depths above 10%, indicating that this behavior is common across different LLPS systems. With appropriate normalization (Supplementary Fig. 6), the dip depth was rather independent of the condensate size, the relative size of the bleach region, and the bleach depth (Fig. 2d–f), while the recovery time changed with the condensate size and the size of the bleach region as expected (Supplementary Fig. 7a). For smaller condensates, the signal-to-noise ratio decreased, potentially limiting the robustness of the measurement. In our hands, condensates with radii down to ∼1 µm yielded reliable results (Fig. 2d and Supplementary Fig. 7b).

We next conducted half-FRAP experiments in living cells, using the models described above. We observed a large dip depth of 45 ± 3% for DDX4-YFP in liquid-like nuage granules (Fig. 2g), and a small dip depth of 5 ± 2% for the engineered CD*-YFP fusion that acts as a low-valency binder of H3K9me2/3 clusters in heterochromatin foci (Fig. 2i). Consistently, we also obtained dip depths that were significantly larger than 10% for other liquid-like condensates, i.e., for the RNA-binding protein FUS in cytosolic stress granules (Fig. 2h, *p* = 2 × 10^−10^), for NPM1 in nucleoli (Supplementary Fig. 8a, c,*p* = 6 × 10^−5^), and for the intrinsically disordered RGG repeats of LAF-1 in cytosolic condensates (Supplementary Fig. 8b, d,*p* = 1 × 10^−4^). These results indicate that preferential internal mixing, reflected by dip depths that are significantly larger than 10%, signals the presence of an interfacial barrier in cellular structures formed by LLPS.

### Theory and simulations show that attractive intermolecular interactions give rise to an interfacial barrier that induces preferential internal mixing

**Fig. 3:**
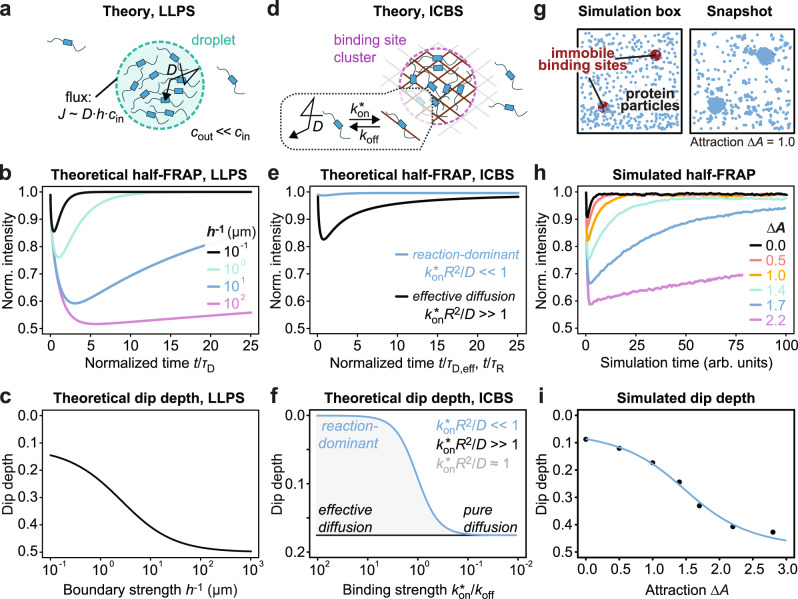
Theoretical and simulated half-FRAP curves relate the dip depth to the underlying molecular properties. **a** Model describing the LLPS scenario. The condensate interface was modeled as a boundary that attenuates the diffusive flux. **b** Theoretically predicted half-FRAP curves in the non-bleached half for the LLPS model. The curves are plotted versus time divided by the characteristic diffusion time *τ*_D_ = *R*^2^/4*D*, showing that the dip depth is independent of the diffusion coefficient *D* and condensate radius *R*. **c** Relationship between dip depth and boundary strength *h*^−1^ in the LLPS model. **d** Model describing the ICBS scenario. The binding reaction was modeled as a pseudo first-order reaction with rates ${k}_{{{{{{\rm{on}}}}}}}^{*}$ and *k*_off_, where each particle could interact with only one binding site at a time. **e** Theoretically predicted half-FRAP curves in the non-bleached half for the ICBS model. For fast binding (${k}_{{{{{{\rm{on}}}}}}}^{*}$R^2^/*D* » 1), the model converges to a pure diffusion model with an effective diffusion coefficient *D*_eff_ = *D*/(1+${k}_{{{{{{\rm{on}}}}}}}^{*}$/*k*_off_). For strong and slow binding (${k}_{{{{{{\rm{on}}}}}}}^{*}$R^2^/*D* « 1), it converges to a reaction-dominant model in which the recovery is governed by the dissociation rate. The respective curves are plotted versus time divided by *τ*_D,eff_ = *R*^2^/4*D*_eff_ (black curve) and *τ*_R_ = 1/*k*_off_ (blue curve), showing that the dip depth is independent of the effective diffusion coefficient and the dissociation rate, respectively. **f** Relationship between dip depth and binding strength ${k}_{{{{{{\rm{on}}}}}}}^{*}$/*k*_off_ in the ICBS model. **g** Schematic representation of the simulation setup including two clusters of immobile binding sites (red), mobile protein particles (blue) and solvent particles (not depicted). A representative snapshot showing the protein distribution after the number of proteins at each binding site cluster had reached a plateau is shown to the right. **h** Simulated half-FRAP curves in the non-bleached half for proteins with different intermolecular interaction strength ∆*A*. For ∆*A* < 1, the simulation corresponds to ICBS; for ∆*A* ≥ 1, the simulation corresponds to LLPS, with the system moving deeper into the 2-phase regime with increasing ∆*A*. The number of interaction partners was not restricted. **i**, Relationship between dip depth and intermolecular interaction strength ∆*A*.

To gain more insight into preferential internal mixing from a theoretical perspective, we calculated half-FRAP curves for molecules diffusing in a circular domain surrounded by a semi-permeable boundary, assuming a negligible concentration of molecules outside of the domain (Fig. 3a). The strength of the boundary is determined by the parameter *h*, which determines the flux at the boundary and whose inverse can be understood as the effective thickness of the boundary^22^. This model describes the LLPS scenario with a strong enrichment in the dense phase. The calculations show that the dip depth depends on the boundary strength *h*^−1^ (Fig. 3b, c) but not on the diffusion coefficient of the molecules, which only determines the scaling of the curves in Fig. 3b along the time axis. This is intuitive, as the dip depth reflects the asymmetry of the recovery process, i.e., the preference of molecules to leave the bleached half via the side facing the non-bleached half rather than the side facing the surrounding phase, which is independent of the actual time that molecules need to reach one of these sides. More details about these calculations are presented in *Half-FRAP model for diffusion in a circle with a semi-permeable boundary* in the “Methods” section.

Next, we calculated half-FRAP curves for molecules diffusing and interacting with immobile binding sites located in a circular domain (Fig. 3d), which rationalizes ICBS involving a long polymeric scaffold that remains within the domain during the recovery process. Interactions with binding sites are described by the rate constants ${k}_{{{{{{\rm{on}}}}}}}^{*}$ and *k*_off_, whose ratio can be understood as the binding strength. In the previously discussed limits of slow and fast binding^8^, FRAP curves can be plotted against normalized times that depend on the binding rates (Fig. 3e, see figure legend for details). While the dip depth converges to that for free diffusion in the limit of fast binding (Fig. 3e, black curve), the dip depth decreases in the limit of strong and slow binding (Fig. 3e, blue curve). This behavior is expected, as reaction–diffusion processes converge to free diffusion with a reduced effective diffusion coefficient in the limit of fast binding^8^. In the limit of strong and slow binding, molecules dissociate only sporadically from their binding sites to then quickly diffuse away, so that the number of bleached molecules found in the non-bleached half at a given time is negligible. For the intermediate case in which binding and diffusion occur on similar time scales, intermediate dip depths are obtained (Fig. 3f, gray area). More details about these calculations are presented in *Half-FRAP model for a reaction-diffusion process in a circle with a fully permeable boundary* in the “Methods” section.

In summary, the results in Fig. 3c, f show that ICBS coincides with dip depths that are equal or smaller than that for free diffusion in a homogenous solution, while LLPS coincides with dip depths that are larger than that for free diffusion. We next sought to quantitatively relate the dip depth to the underlying molecular interactions, which determine the boundary strength in the LLPS model above. Since the dip depth changed with the salt concentration in PLL-HA coacervates, we hypothesized that it is related to the strength of the attractive cohesive interactions among the molecules that drive LLPS. To test this hypothesis, we conducted coarse-grained dissipative particle dynamics (DPD) simulations of half-FRAP experiments with molecules exhibiting different intermolecular interaction strengths. We used a simulation box with two clusters of immobile binding sites where structures of interest could form (Fig. 3g), mimicking ICBS for weak attractive interactions and LLPS for sufficiently strong attractive interactions. The simulations showed that the dip depth indeed increased with increasing strength of attractive interactions (Fig. 3h), with a sigmoidal relationship between both quantities (Fig. 3i). Accordingly, the attractive intermolecular interactions that drive LLPS give rise to an interfacial barrier that induces preferential internal mixing, resulting in an increased dip depth in half-FRAP curves.

### Calibrated half-FRAP quantifies the interfacial barriers of biomolecular condensates

**Fig. 4:**
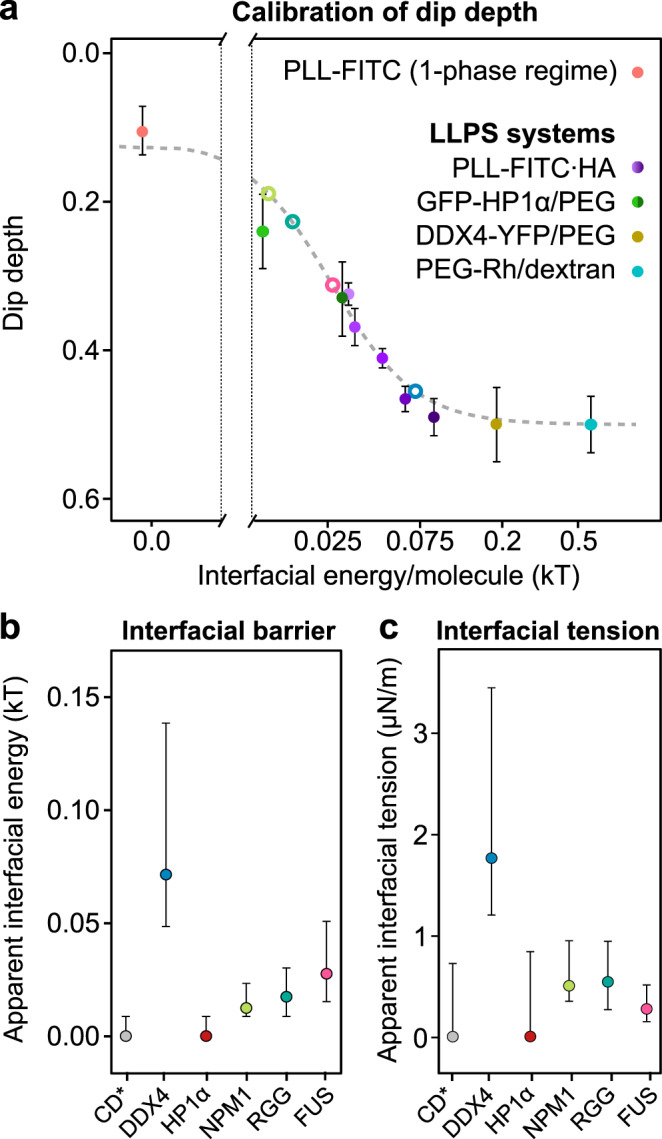
MOCHA-FRAP quantifies interfacial barriers based on the dip depth. **a** Relationship between dip depth and interfacial energy per molecule for PLL-HA undergoing LLPS (violet; magnesium chloride concentrations increase from dark to light color), GFP-HP1α/PEG (dark green: without ssDNA; light green: with ssDNA), DDX4-YFP/PEG (gold) and PEG-Rhodamine/dextran (cyan) measured in vitro. As a reference, free PLL in the 1-phase regime is shown (red). The error bars represent the standard error of the mean. Open circles represent dip depths obtained in live-cell experiments, following the same color code as in the panels below. Apparent interfacial energy per molecule, which serves as a proxy for the interfacial barrier (**b**), and apparent interfacial tension (**c**) calculated from the dip depth of CD*-YFP, DDX4-YFP, GFP-HP1α, NPM1-GFP, RGG-GFP-RGG and FUS-mCherry in living cells, using the calibration curve in panel a. Error bars represent the standard error of the mean. Source data are provided as a Source Data file.

We next sought to quantitatively relate the dip depth to the energetics underlying LLPS. To obtain the relevant parameters for the in vitro LLPS model systems used above, i.e., PLL-HA, PEG-Rhodamine/dextran, DDX4-YFP/PEG and GFP-HP1α/PEG, we first analyzed fusion events of the respective condensates (Supplementary Fig. 9), yielding the inverse capillary velocity that corresponds to the ratio of viscosity to surface tension, *η*/*γ* (Supplementary Fig. 10). Next, we determined the viscosity *η* of the condensates by measuring the diffusion of molecules inside of them (Supplementary Figs. 11, 12). These two measurements allowed us to retrieve the interfacial tension and, together with the estimated size of the molecules, the interfacial energy per molecule (see *Calculation of the interfacial energy per molecule* in the “Methods” section for details). For simple enough systems, the latter is expected to scale with the strength of the attractive cohesive interactions among molecules^23–25^, which have to be broken when a molecule leaves the condensate. We, therefore, hypothesized that it can serve as a proxy for the barrier that molecules in the condensate have to overcome when crossing the interface. We indeed obtained a sigmoidal master curve when plotting the dip depth, which reflects the strength of the interfacial barrier, versus the interfacial energy per molecule for the different in vitro LLPS systems we used here (Fig. 4a). This curve resembles the sigmoidal relationship between the dip depth and the interaction strength we obtained from the simulations above (Fig. 3i). The low energy values we determined here are consistent with the literature^14,26^, which suggests that the respective condensates might not only be stabilized by attractive cohesive interactions but also by entropic contributions from the release of hydration water or counterions^14^.

Based on the master curve obtained with the in vitro systems, we used the dip depth extracted from half-FRAP experiments in living cells to quantify the interfacial barriers for DDX4-YFP in nuage granules, NPM1-GFP in nucleoli, and RGG-GFP-RGG (derived from LAF-1) and FUS-mCherry in cytoplasmic condensates (Fig. 4b). In addition, we reanalyzed published data for GFP-HP1α in heterochromatin foci^4^ according to the same workflow we used here for the other proteins. Under the assumption that the attractive intermolecular interactions established by the labeled proteins of interest govern the global behavior of the respective cellular structure, we also calculated apparent interfacial tensions (Fig. 4c). This assumption is expected to hold true for condensates that (i) do not show additional interfacial resistance^20^, so that their interfacial barrier arises exclusively from attractive intermolecular interactions, and (ii) lack additional compounds that modulate their mechanical properties, such as other phase-separating proteins or long polymeric scaffolds like DNA/chromatin^27,28^.

On the one end of the spectrum of interfacial barriers in living cells, the small dip depths obtained for CD*-YFP and GFP-HP1α in heterochromatin foci revealed the absence of any barrier, indicating that both proteins undergo ICBS. This contrasts with the barriers of ~0.01 kT and ~0.03 kT that we obtained for GFP-HP1α/PEG condensates with and without single-stranded DNA in vitro, respectively, which are formed by LLPS. On the other end of the spectrum, the interfacial barrier for DDX4-YFP in nuage granules amounted to ~0.07 kT, which was the highest barrier we observed in living cells. It is smaller than the value we obtained for DDX4-YFP/PEG in vitro (~0.18 kT), which might mean that cellular nuage granules contain other molecules, such as RNAs, which reduce the interfacial barrier. The interfacial barrier for NPM1 amounted to ~0.01 kT, which corresponds to an apparent interfacial energy of ~0.5 µJ/m^2^ that is in agreement with previous rheological measurements of nucleoli in HeLa cells^29^. This correspondence suggests that the same molecular interactions that give rise to preferential internal mixing of NPM1 might also determine the rheological properties of the nucleolus. The interfacial barrier for FUS-mCherry amounted to ~0.03 kT, which corresponds to an apparent interfacial energy of ~0.3 µJ/m^2^ and falls in the range of values reported for FUS and FUS-DNA condensates in vitro^26,30,31^. The apparent interfacial energy of ~0.5 µJ/m^2^ that we obtained for RGG-GFP-RGG condensates is about 300-fold lower than the values reported for full-length LAF-1^32^ and RGG-RGG in vitro^26^. We speculate that this difference is at least partly due to the presence of RNA within cellular RGG-GFP-RGG condensates, which has been shown to change the properties of LAF-1 condensates^32^.

The results above show that cellular condensates differ from each other with respect to their interfacial properties, reflecting differences in the underlying molecular interactions that likely translate into functional differences. The interfacial properties of condensates reconstituted in vitro can significantly differ from those of their cellular counterparts, and the conditions used to induce LLPS in vitro, such as the concentration of salt and nucleic acid components, can strongly affect these properties (Fig. 4 and Supplementary Table 4). For example, GFP-HP1α condensates reconstituted in vitro with or without single-stranded DNA exhibit different interfacial barriers, while the absence of an interfacial barrier for GFP-HP1α in heterochromatin foci indicates that HP1α does not undergo LLPS in living mouse fibroblast cells (Supplementary Fig. 5). The latter might be due to the long chromosomal segments that are present in heterochromatin foci, which can be expected to influence the properties of these foci and the proteins they contain^28^. These observations highlight the importance of live-cell experiments to complement in vitro work with minimal systems, and we anticipate that MOCHA-FRAP will be a valuable tool in this respect.

### MOCHA-FRAP – a tool to study membrane-less structures in vitro and in living cells

We introduce here a model-free calibrated half-FRAP (MOCHA-FRAP) approach to detect membrane-less condensates formed by LLPS via the signature of preferential internal mixing. We demonstrate that MOCHA-FRAP can distinguish LLPS from the alternative scenario of molecules interacting with spatially clustered binding sites on a long polymeric scaffold without undergoing LLPS. We show that MOCHA-FRAP can retrieve quantitative information about the characteristic energy barrier at the interface of cellular substructures, which molecules have to overcome when exchanging with the surrounding medium. This barrier represents an intuitive measure of how separated the protein pools inside and outside of a cellular substructure are, which might in some contexts be more instructive than identifying the occurrence of LLPS per se. For the panel of proteins and cellular substructures we studied here, we find that the interfacial barriers are quite low, with energies well below the thermal energy. This is consistent with the fast molecular exchange and the small interfacial tensions recently reported for different biomolecular condensates^1,2,26^. By comparison, interfacial barriers for membrane-bound structures tend to be much larger, with energies well above the thermal energy^33,34^.

MOCHA-FRAP is compatible with live-cell experiments using commercial confocal microscopes. A tutorial providing a compact overview of the workflow and the interpretation of the results is contained in Supplementary Note 2, and an interactive analysis pipeline is available online (see link in the “Code availability”). MOCHA-FRAP has the typical limitations of fluorescence microscopy, namely that the protein of interest has to be labeled and that the structure of interest has to be large enough to be resolved and half-bleached. We anticipate that MOCHA-FRAP will facilitate the study of phase separation processes in biology.

## Methods

### Material

Information about all the materials used in this work is detailed in Table S1.

### Preparation of PLL-HA coacervates and cross-linked PLL-HA coacervates

Solutions of Poly-L-Lysine hydrobromide (PLL, 15–30 kDa; P7890, Sigma), Poly-L-Lysine-FITC hydrobromide (PLL-FITC, 15–30 kDa; P3543, Sigma) and Hyaluronic Acid sodium salt (HA, 8–15 kDa; 40583, Sigma) were prepared in 50 mM Tris-Cl pH 8 at a concentration of 10 mg/mL. FITC-labeled PLL was spiked into unlabeled PLL at a 1:500 ratio. PLL-HA coacervates were prepared by mixing PLL and HA at a mass ratio of 1:4 and a final concentration of 10 mg/mL^14^, resulting in net charge neutralization. This solution was diluted 1:10 in 50 mM Tris-Cl pH 8, 7.5% PEG, with or without MgCl_2_ (25 mM, 50 mM, 100 mM, or 150 mM), to obtain the coacervate suspensions used for the experiments.

In order to prepare cross-linked PLL-HA coacervates, Poly-L-Lysine hydrobromide (PLL, 15–30 kDa; P7890, Sigma), Hyaluronic Acid sodium salt (HA, 8–15 kDa; 40583, Sigma) and Hyaluronate Rhodamine (HA-Rh, 10 kDa; HA-Rhodamine-10k, HAWorks) were dissolved in PBS at 10 mg/mL. Rhodamine-labeled HA was spiked into unlabeled HA at a 1:500 ratio. Formation of coacervates and cross-linking was carried out in a single step in cross-linking solution containing 1 mg/mL PLL-HA (HA-Rh 1:500), 7.5% PEG, 0.4 mg/mL 1-ethyl-3-(3-dimethylaminopropyl) carbodiimide (EDC) and 1.1 mg/mL N-Hydroxysulfosuccinimide sodium salt (sulfo-NHS). The mix was incubated for 2 h at room temperature before the reaction was quenched by addition of an equal volume of 50 mM Tris-Cl pH 8. The cross-linked coacervates were sedimented by centrifugation and resuspended in an equal volume of fresh 50 mM Tris-Cl pH 8 buffer. To study PLL undergoing ICBS at cross-linked coacervates, the latter were diluted 1:10 in a solution of 0.1 mg/mL PLL-FITC and incubated at room temperature for 30 min before the experiments were carried out.

In order to prepare ATTO 647N-labeled PLL-HA coacervates used for FCS experiments, PLL at a concentration of 10 mg/mL was labeled with ATTO 647N NHS ester (AD 647N-31, ATTO-TEC) according to the manufacturer’s instructions. Free ATTO 647N dye was removed using a Zeba MicroSpin desalting column 7 K MWCO (89877, Thermofisher). PLL-ATTO 647N was spiked into unlabeled PLL at a 1:10,000 ratio, and coacervates were prepared as described above.

### PEG labeling and preparation of PEG/dextran droplets

Rhodamine-labeled PEG was prepared using NH2-PEG-NH2 (8 kDa; PG2-AM-8K, NANOCS) and NHS-Rhodamine (46406, Thermofisher), according to the manufacturer’s instructions, and was purified using a Zeba MicroSpin desalting column. Labeled PEG was spiked into unlabeled PEG (40% v/v) at a 1:10 ratio, mixed with FITC-Dextran (150 kDa; 46946, Sigma) at final concentrations of 1% v/v PEG and 20% v/v dextran, and incubated for 15 min at room temperature to form PEG droplets (minority phase) within dextran (majority phase).

### Plasmids

For 1,6-hexanediol assays, we used a DDX4-YFP plasmid^16^ and a CD-mKate plasmid (generated from the previously described CD-RFP plasmid^35^, which encodes aa 10–80 of CBX1/HP1β, by replacing RFP with mKate2) for transfection as described below. For FRAP experiments, we used the same DDX4-YFP plasmid^16^ (LLPS model) and a plasmid encoding an engineered chromodomain of CBX1/HP1β (CBX1-V22E/K25E/D59S), which binds strongly to H3K9me2/3^17^ (ICBS model). To use the same fluorescent protein for both LLPS and ICBS, we replaced the sequence coding for PA-mCherry in the plasmid pcDNA3-CBX1mut-PA-mCherry (Addgene plasmid # 138251) by the sequence coding for YFP (obtained from the DDX4-YFP plasmid mentioned above). For half-FRAP in living cells, we also used plasmids coding for NPM1-GFP^4^, RGG-GFP-RGG^36^ (Addgene plasmid # 124939) and FUS-mCherry^37^.

For expression of recombinant GFP-HP1α, the previously described pET28/GFP-HP1α plasmid was used^4^. For expression of recombinant DDX4-YFP, the sequence coding for DDX4-YFP was amplified from the DDX4-YFP plasmid for mammalian expression mentioned above^16^ by PCR (see Table S1 for primer sequences), and the PCR product was digested with BamHI and EcoRI and inserted into a pGEX-GST expression plasmid.

### Cell culture, transfection and live-cell experiments

NIH 3T3 cells (ATCC CRL-1658) were grown at 37 °C in a humidified atmosphere with 5% CO_2_, in Dulbecco’s Modified Eagle Medium (DMEM) supplemented with 10% fetal bovine serum (FBS; 11550356, Life Technologies), 1% Penicillin/Streptomycin (P4333, Sigma) and 1× Gibco GlutaMAX. For microscopy experiments, cells were seeded in LabTek chambered coverslips (155411, NUNC) at a density of 40,000 cells per well and transfected with Lipofectamine 2000 (11668019, Invitrogen) according to the manufacturer’s protocol.

### Purification of recombinant proteins and preparation of the respective condensates

The GST-DDX4-YFP construct was expressed in *E. coli* Rosetta cells (70956, Sigma). Cells were grown in LB medium supplemented with 1 mM IPTG at 18 °C overnight. Subsequently, cells were pelleted and resuspended in lysis buffer (150 mM NaH_2_PO_4_ pH 7.5, 300 mM NaCl, 10 mM imidazole, 25% glycerol, 4% sarkosyl, 1000 U benzonase, 1 mg/mL lysozyme, 0.5% Triton X-100, 1 mM DTT, 0.1 mM PMSF). Cells were sonicated and the supernatant was loaded onto a GSTrap FF column (17-5130-01, GE Healthcare) and washed with washing buffer (50 mM Tris-Cl pH 8, 300 mM NaCl, 2.5 mM DTT). Subsequently, 2 mL of 3C-PreScission enzyme were loaded onto the column and incubated at 4 °C for 4 h. DDX4-YFP was eluted and collected in fractions. Eluates were dialyzed against storage buffer without glycerol (20 mM HEPES pH 7.8, 200 mM KCl, 1 mM DTT). The protein concentration was determined by UV-spectroscopy, using the theoretical extinction coefficient *ε*_DDX4-YFP_ = 32,680 M^−1^ cm^−1^ at 280 nm. To prepare DDX4-YFP condensates in vitro, DDX4-YFP was diluted to 150 µM in 100 mM Tris-Cl pH 8, 150 mM NaCl, 1% PEG 20,000 (813000, Fluka).

Recombinant GFP-HP1α was purified as previously described^4^. To prepare GFP-HP1α condensates in vitro, GFP-HP1α was diluted to 50 µM in 100 mM Tris-Cl pH 8, 300 mM CaCl_2_, 0.33% PEG 20,000 (813000, Fluka). When indicated, a single-stranded 96-mer DNA oligonucleotide (see Table S1 for details) was added at a final concentration of 50 µM.

### 1,6-Hexanediol assay

NIH 3T3 cells expressing both DDX4-YFP and CD-mKate were visualized on a Zeiss LSM 710 confocal light scanning microscope (Carl Zeiss, Oberkochen, Germany), equipped with a 63x/NA 1.2 oil immersion objective. The cell culture medium was replaced by Leibovitz’s L-15 medium (21083-027, Themofisher) before the experiment, and experiments were conducted at 37 °C. Prior to 1,6-hexanediol treatment, a *z*-stack of a field-of-view containing multiple cells was recorded. Subsequently, 1,6-hexanediol was gently added to a final concentration of 10% (v/v). Images were acquired each 2 min, and a final *z*-stack was recorded after 5 minutes. Initial and final *z*-stacks were transformed into maximum *z*-projections and were analyzed using an R script^38–40^ to quantify the signal enrichment at structures of interest.

### Fluorescence correlation spectroscopy measurements

FCS measurements were performed with a custom-built confocal microscope based on an ASI Rapid Automated Modular Microscope (RAMM) system with added precision objective scanning by a one-axis piezo scanner (P-721 PIFOC) and a two-axis piezo stage (P-733.2CD, both Physik Instrumente). Excitation light from a Picoquant LDH-D-C-640B picosecond diode laser was coupled into a polarization-maintaining fiber (Schäfter & Kirchhoff) and was linearly polarized with a film polarizer (Thorlabs). The laser light was reflected by a dichroic mirror (z532/640, CHROMA) towards a 100× NA 1.45 objective (Alpha Plan-Fluar, Zeiss). Emitted fluorescence light passed the dichroic and was filtered with a notch filter (488/532/631-640 nm, AHF Analysetechnik). After spatial filtering with two 75 mm lenses and a 100 µm pinhole, fluorescence light was split into parallel and perpendicular components with a broadband polarizing beam splitter (PBS201, Thorlabs). Each component was split again with 50:50 beamsplitters (Thorlabs) and focused (focal length 200 mm) onto in total 4 SPAD detectors (SPCM AQR-14, Perkin-Elmer). Detected signals were processed with a HydraHarp 400 multichannel time-correlated single photon counting system and the SymPhoTime 64 software platform (both PicoQuant).

Samples for FCS were prepared in 8-well chambered LabTek coverslips. For solution measurements, glycerol was mixed with 50 mM Tris-Cl pH 8 to obtain the specified concentration, and labeled PLL was added to reach a final concentration of 5 nM. For measurements in PLL-HA coacervates, chambers were incubated with 30% PEG (v/v) for 15 min to passivate the surface. ATTO 647N-labeled PLL-HA coacervates were then prepared as described above. FCS measurements in solution were conducted approximately 5 µm above the glass surface, measurements in droplets were conducted above the surface close to the center of the droplet. All FCS measurements were conducted at 20 µW average excitation power for 2 min. The first 20 s of the data were discarded to exclude bleaching of an immobile fraction.

FCS data were pre-binned into bins of 1.25 µs and then cross-correlated between all detectors using the multipletau algorithm implemented in the multipletau python package^41^. Averaged correlation curves were globally fitted with the function

1 $$G\left(\tau \right)=\frac{1}{N}{\left(1+{\left(\frac{\tau }{{\tau }_{{{{{{\rm{D}}}}}}}}\right)}^{\alpha }\right)}^{-1}{\left(1+\frac{1}{{\kappa }^{2}}{\left(\frac{\tau }{{\tau }_{{{{{{\rm{D}}}}}}}}\right)}^{\alpha }\right)}^{-\frac{1}{2}}\left(1+{f}_{{{{{{\rm{T}}}}}}}\exp \left(-\frac{\tau }{{\tau }_{{{{{{\rm{T}}}}}}}}\right)\right)$$

where *N* is the number of particles in the focal volume, *τ*_D_ is the translational diffusion time, *τ*_T_ is the triplet correlation time, *f*_T_ is the triplet amplitude, *α* is the anomaly parameter, and *κ* is the structural parameter, which was held fixed for the fit.

### Droplet coalescence measurements

Initially, the surface of 8-well chambered LabTek coverslips was washed with water and then with rinse buffer (10 mM Tris-Cl pH 8, 100 mM NaCl), followed by an incubation with a liposome mix for passivation, which contained 10 mg/mL 1,2-dioleoyl-sn-glycero-3-phosphocholine (DOPC, 850375P, Avanti lipids) and 1 mg/mL 1,2-dioleoyl-sn-glycero-3-phosphoethanolamine-N-[methoxy(polyethylene glycol)−2000] ammonium salt (DOPE-PEG, 880130P, Avanti lipids) in rinse buffer and which was prepared as previously described^42^. Subsequently, the liposome mix was removed and the coverslips were washed with rinse buffer. Freshly prepared PLL-HA coacervates, PEG/dextran droplets or DDX4-YFP/PEG condensates (5 µL) were loaded onto the passivated chamber, and coalescence events were recorded with a custom-built microscope equipped with an Olympus halogen light source, excitation/emission filters (MDF-FITC and MDF-MCHA, Thorlabs), a 60×/NA 1.2 water objective, and an Andor iXon Ultra 897 EM-CCD camera. Movies with a length of 5 min were recorded at a frame rate of 83 Hz (one frame each 12 ms) to capture fusion events of droplets of different sizes near the glass surface. Additional experiments, in which samples were centrifuged prior to the measurement to induce the formation of larger droplets, were also conducted. The images were analyzed with an R script. The inverse capillary velocity was determined as previously described^14^. Briefly, for each fusion event, the eccentricity *e* = (*R*_max_ − *R*_min_)/(*R*_max_ + *R*_min_) was measured at each time point, and its relaxation was fitted to a formula of the form *e*(*t*) = *e*_post_ + (*e*_pre_ − *e*_post_)·exp(−*t*/*τ*_e_), where *e*_pre_ and *e*_post_ are the eccentricities before and after coalescence, respectively, and *τ*_e_ is the relaxation time. The inverse capillary velocity (*η*/*γ*) was determined by fitting the relaxation time versus the final size of the droplet after coalescence (*R*_final_) to a linear equation of the form *τ*_e_(*R*_final_) = (*η*/*γ*)·*R*_final_.

### Calculation of the interfacial energy per molecule

To determine the interfacial energy per molecule in a condensate formed by LLPS, we first determined the viscosity and the interfacial tension of the respective condensate (for PLL-HA, PEG-Rhodamine/dextran, DDX4-YFP/PEG and GFP-HP1α/PEG). First, the viscosity *η* of PLL-HA coacervates was determined from the translational diffusion times of PLL within coacervates and in glycerol-water mixtures of known viscosity^43^ obtained by FCS (Supplementary Fig. 12a, b). The diffusion times for PLL-HA coacervates were also estimated from half-FRAP. As half-FRAP has a lower time resolution than FCS, it is expected to only yield accurate results for sufficiently slow diffusion processes. To determine diffusion times with half-FRAP, we measured the recovery of the bleached half (*F*_half_) and of the entire droplet (*F*_full_) in the same half-FRAP experiment. The first quantity (*F*_half_) depends on both internal diffusion in the condensate and exchange across the boundary of the condensate, while the second quantity (*F*_full_) only depends on the exchange across the boundary of the condensate. To focus only on the diffusion within the condensate, we calculated the difference between both quantities:

2 $${F}_{{{{{{\rm{diff}}}}}}}\left(t\right)={F}_{{{{{{\rm{half}}}}}}}\left(t\right)-{F}_{{{{{{\rm{full}}}}}}}\left(t\right)$$

Subsequently, we fitted this quantity to a simple diffusion model:^44^

3 $${F}_{{{{{{\rm{diff}}}}}}}\left(t\right)={{Ae}}^{-\frac{{2\tau }_{{{{{{\rm{D}}}}}}}}{t}}\left[{I}_{0}\left(2{\tau }_{{{{{{\rm{D}}}}}}}/t\right)+{I}_{1}\left(2{\tau }_{{{{{{\rm{D}}}}}}}/t\right)\right]$$

where *I*_0_ and *I*_1_ are modified Bessel functions and *τ*_D_ = *R*^2^/(4*D*), with *R* being the radius of the bleach region and *D* the diffusion coefficient. As we found a linear relationship between the diffusion times obtained with this half-FRAP approach in PLL-HA coacervates and the diffusion times obtained with FCS in the same types of coacervates (Supplementary Fig. 12c), we concluded that the half-FRAP approach is valid for the respective range of diffusion times. We next determined the diffusion times for PEG-Rhodamine/dextran, DDX4-YFP/PEG and GFP-HP1α/PEG condensates by half-FRAP, resulting in even larger values (slower diffusion/higher viscosity) compared to those obtained in PLL-HA coacervates, indicating that diffusion in these condensates is slow enough to be assessed by half-FRAP.

Using the viscosity *η* and the inverse capillary velocity *η*/*γ* determined by droplet coalescence measurements (Supplementary Fig. 10), we determined the interfacial tension *γ* = *η*/(*η*/*γ*), which reflects the force acting on a molecule at the interface or, equivalently, the energy that is required to increase the interfacial area of the condensate. Based on this value, we calculated the interfacial energy per molecule. To this end, we approximated each molecule as a sphere with a radius that equals its hydrodynamic radius *R*_h_ (see Table S3). Assuming that each molecule at the interface contributes a surface that equals half of this sphere, the interfacial energy per molecule amounts to

4 $${\varDelta G}_{{{{{{\rm{interface}}}}}}/{{{{{\rm{molecule}}}}}}}=\gamma \left(4\pi{R}_{{{\rm{h}}}}^{2}\right)/2$$

For a simple enough system, i.e., in the absence of surfactants or other compounds that would modulate the properties of the interface, the interfacial energy is expected to scale with the attractive cohesive interactions among the phase-separating molecules^23–25^, which are broken when a molecule leaves the condensate.

### Coarse-grained simulations of half-FRAP experiments

Molecular dynamics simulations of half-FRAP experiments were conducted using LAMMPS^45^ along with the USER-DPD package. A force field for DPD^46^ was used to describe interactions among particles. It includes a conservative repulsive force among particles that is proportional to the parameter *A*, a dissipative force that is proportional to the parameter *γ*, and a random force that is proportional to the parameter *σ*. The dissipative and the random force are related to each other by the fluctuation-dissipation theorem, so that it is sufficient to choose either *γ* or *σ*. The simulations shown in Fig. 3 were conducted with *γ* = 10^6^, *A*_11_ = *A*_22_ = 12, *A*_23_ = 10, and *A*_12_ = *A*_13_ = 12 + ∆*A* (units ‘nano’). Here, *A*_ij_ denotes the interaction between molecules of type *i* and *j*, with types 1,2 and 3 corresponding to solvent particles, protein particles and immobile particles acting as immobile binding sites. The parameter ∆*A* was varied between 0 and 3 as indicated in Fig. 3. The size of the unit cell was 68, and the density of DPD particles per unit cell was 3. Simulations were run under isothermal conditions using the Shardlow splitting algorithm to integrate the DPD equations of motion. To simulate half-FRAP experiments, particles residing in the respective region of interest (ROI) in a chosen bleach frame were marked and subsequently followed. Several FRAP curves were obtained from a single simulation by considering different frames as bleach frames, and the resulting curves were averaged.

### Full-, partial- and half-FRAP measurements

Fluorescence recovery after photobleaching (FRAP) experiments were performed on a Zeiss LSM 710 confocal light scanning microscope (Carl Zeiss, Oberkochen, Germany), equipped with a 63×/NA 1.2 oil immersion objective. Live-cell experiments were performed with transfected cells in Leibovitz’s L-15 medium incubated at 37 °C. For in vitro experiments, samples (5 µL) were placed on 8-well chambered LabTek coverslips that had been passivated beforehand with 15% PEG and extensively rinsed. The in vitro experiments were conducted within the first 15 min after pipetting the sample and at room temperature to minimize evaporation effects. Typically, images were acquired at 128 × 512 pixels at a scan speed corresponding to 200 ms per image, and 300 images were acquired over 5 min (or 75 s for live-cell experiments), with an interval of 800 ms between subsequent images (or 0 ms for live-cell experiments). Before photobleaching, 3 to 5 images were recorded. Bleaching parameters, i.e., laser intensity and scanning time, were chosen to reach approximate 50% of bleaching in the shortest possible time. The bleaching area was selected according to the type of experiment (full-, partial- or half-FRAP). In the case of half-FRAP, which relies on the analysis of the bleached and the non-bleached half, it is important to optimize the bleaching step (intensity and area of the bleaching spot) to minimize bleaching in the other (non-bleached) half. Half-FRAP experiments were conducted until the signals in both halves had converged to each other or until the signal in the non-bleached half had reached its initial pre-bleach value.

### Full- and partial-FRAP data analysis

Approximately 10 FRAP experiments were performed and averaged to get a single FRAP curve (see *Statistics and Reproducibility*). For each experiment, an unbleached object in the field of view was used as internal reference to quantify unwanted acquisition photobleaching. In the case of full- and partial-FRAP, curves were calculated according to

5 $${{{{{{\rm{FRAP}}}}}}}_{{{{{{\rm{full}}}}}}/{{{{{\rm{part}}}}}}}\left(t\right)=\frac{{I}_{{{{{{\rm{B}}}}}}}(t)-{I}_{{{{{{\rm{BG}}}}}}}(t)}{{I}_{{{{{{\rm{REF}}}}}}}(t)-{I}_{{{{{{\rm{BG}}}}}}}(t)}$$

Here, *I*_B_, *I*_NB_, *I*_BG_ and *I*_REF_ denote the average intensities in the bleached and non-bleached ROI, the background of the image and the non-bleached internal reference, respectively. Next, the FRAP curves were normalized with respect of the number of bleached molecules:

6 $${{{{{\rm{FRAP}}}}}^\prime}{ }_{{{{{{\rm{full}}}}}}/{{{{{\rm{part}}}}}}}(t)=\frac{{{{{{{\rm{FRAP}}}}}}}_{{{{{{\rm{full}}}}}}/{{{{{\rm{part}}}}}}}\left(t\right)-{{{{{{\rm{FRAP}}}}}}}_{{{{{{\rm{full}}}}}}/{{{{{\rm{part}}}}}}}\left({t}_{{{{{{\rm{bleach}}}}}}}\right)}{{{{{{{\rm{FRAP}}}}}}}_{{{{{{\rm{full}}}}}}/{{{{{\rm{part}}}}}}}({t}_{{{\rm{pre}}}})-{{{\rm{FRAP}}}}_{{{\rm{full}}}/{{{\rm{part}}}}}\left({t}_{{{\rm{bleach}}}}\right)}$$

Here, *t*_bleach_ and *t*_pre_ are the acquisition times of the first post-bleach frame and the last pre-bleach frame, respectively. Thus, full- and partial-FRAP curves are double-normalized, i.e., they equal unity before bleaching and zero in the first post-bleach frame. Since for a diffusion process, the recovery time scales with the squared size of the bleach ROI (*R*^2^), we normalized the time as *t*_norm_ = *t*/*R*^2^ to compare curves with different-sized bleach regions to each other.

### Half-FRAP data analysis

To analyze half-FRAP experiments, an R script was used to segment the images and retrieve the average intensity of the bleached half (*I*_B_), the non-bleached half (*I*_NB_), the background of the image (*I*_BG_) and a non-bleached structure (*I*_REF_) in each frame. These intensity values were used to calculate FRAP curves for the bleached half, *FRAP*_B_(*t*), and the non-bleached half, FRAP_NB_(*t*), according to

7 $${{{{{{\rm{FRAP}}}}}}}_{{{{{{\rm{B}}}}}}/{{{{{\rm{NB}}}}}}}\left(t\right)=\frac{{I}_{{{{{{\rm{B}}}}}}/{{{{{\rm{NB}}}}}}}(t)-{I}_{{{{{{\rm{BG}}}}}}}(t)}{{I}_{{{{{{\rm{REF}}}}}}}(t)-{I}_{{{{{{\rm{BG}}}}}}}(t)}$$

These curves were subsequently normalized as described in the following and as shown in Supplementary Fig. 6.

First, curves were corrected for unwanted photobleaching in the non-bleached half. There might be bleached molecules in the nominal non-bleached half because the focal volume of the microscope overlapped with the non-bleached half, or because the molecules moved from the bleached to the non-bleached half during bleaching. The corrected curves read:

8 $${{{{{{\rm{FRAP}}}}}}}_{{{{{{\rm{B}}}}}}/{{{{{\rm{NB}}}}}}}^{{{{{\rm{I}}}}}}\left(t\right)={{{{{{\rm{FRAP}}}}}}}_{{{{{{\rm{B}}}}}}/{{{{{\rm{NB}}}}}}}\left(t\right)+\left[{{{{{{\rm{FRAP}}}}}}}_{{{{{{\rm{NB}}}}}}}({t}_{{{{{{\rm{pre}}}}}}})-{{{{{{\rm{FRAP}}}}}}}_{{{{{{\rm{NB}}}}}}}\left({t}_{{{{{{\rm{bleach}}}}}}}\right)\right]$$

Here, *t*_pre_ and *t*_bleach_ are the acquisition times of the last pre-bleach frame and the first post-bleach frame, respectively.

Additionally, FRAP_B_ and FRAP_NB_ were multiplied by the size of their respective ROIs (*N*_B_ and *N*_NB_, respectively) to obtain curves that are proportional to the number of particles in each half:

9 $${{{{{{\rm{FRAP}}}}}}}_{{{{{{\rm{B}}}}}}/{{{{{\rm{NB}}}}}}}^{{{{{\rm{II}}}}}}\left(t\right)={{{{{{\rm{FRAP}}}}}}}_{{{{{{\rm{B}}}}}}/{{{{{\rm{NB}}}}}}}^{{{{{\rm{I}}}}}}\left(t\right)\frac{{N}_{{{{{{\rm{B}}}}}}/{{{{{\rm{NB}}}}}}}}{{N}_{{{{{{\rm{B}}}}}}}+{N}_{{{{{{\rm{NB}}}}}}}}$$

Then, the curves were normalized with respect to the number of bleached molecules:

10 $${{{{{{\rm{FRAP}}}}}}}_{{{{{{\rm{B}}}}}}/{{{{{\rm{NB}}}}}}}^{{{{{\rm{III}}}}}}\left(t\right)=\frac{{{{{{{\rm{FRAP}}}}}}}_{{{{{{\rm{B}}}}}}/{{{{{\rm{NB}}}}}}}^{{{{{\rm{II}}}}}}\left(t\right)-{{{{{{\rm{FRAP}}}}}}}_{{{{{{\rm{B}}}}}}/{{{{{\rm{NB}}}}}}}^{{{{{\rm{II}}}}}}\left({t}_{{{{{{\rm{bleach}}}}}}}\right)}{{{{{{{\rm{FRAP}}}}}}}_{{{{{{\rm{B}}}}}}}^{{{{{\rm{II}}}}}}({t}_{{{{{{\rm{pre}}}}}}})-{{{{{{\rm{FRAP}}}}}}}_{{{{{{\rm{B}}}}}}}^{{{{{\rm{II}}}}}}\left({t}_{{{{{{\rm{bleach}}}}}}}\right)}$$

The resulting FRAP curves are proportional to the ROI sizes and double-normalized. Next, an additive offset was applied to the signal in the non-bleached half to normalize to unity before the bleach

11 $${{{{{{\rm{FRAP}}}}}}}_{{{{{{\rm{NB}}}}}}}^{{{{{{\rm{IV}}}}}}}\left(t\right)=1+{{{{{{\rm{FRAP}}}}}}}_{{{{{{\rm{NB}}}}}}}^{{{{{{\rm{III}}}}}}}\left(t\right)$$

In the presence of an “immobile” fraction of molecules that do not move during the course of the experiment because they tightly bind to “immobile” binding sites, the signal in both halves will not recover to the same level but there will be an offset between them that corresponds to the immobile fraction *X*_immobile_. To correct for these “immobile” molecules, which do neither exchange between the two halves nor cross the boundary of the condensate, the FRAP curves are corrected according to

12 $${{{{{{{\rm{FRAP}}}}}}}_{{{{{{\rm{NB}}}}}}}^{{{{{\rm{V}}}}}}\left(t\right)=\frac{{{{{{{\rm{FRAP}}}}}}}_{{{{{{\rm{NB}}}}}}}^{{{{{{\rm{IV}}}}}}}\left(t\right)-1}{1-{X}_{{{{{{\rm{immobile}}}}}}}}+1,\,{{{{{{\rm{FRAP}}}}}}}_{{{{{{\rm{B}}}}}}}^{{{{{{\rm{IV}}}}}}}\left(t\right)=\frac{{{{{{{\rm{FRAP}}}}}}}_{{{{{{\rm{B}}}}}}}^{{{{{{\rm{III}}}}}}}\left(t\right)}{1-{X}_{{{{{{\rm{immobile}}}}}}}}}$$

Here, *X*_immobile_ is the difference between the curves in the bleached and non-bleached half after both of them have reached their plateau.

The resulting curves reflect the change of the number of labeled molecules in each half. For each sample, at least 8–15 FRAP experiments were performed and averaged into a single curve (see *Statistics and Reproducibility*). While the dip depth remained the same when droplets of different sizes were bleached, the rates of recovery varied. Thus, before averaging individual FRAP curves, we normalized the time according to *t*_norm_ = *t*/*R*^2^, where *R* is the radius of the bleached droplet. Afterwards, we multiplied the curves by the mean squared radius 〈*R*〉^2^ to convert back to time.

Finally, averaged FRAP curves were smoothed with a Savitzky–Golay filter^47^ to reduce the contribution of noise (using filter order *p* = 2 and filter length *n* = 21), and the dip depth was numerically determined from the minimum of the smoothed curves. Dip depths that were larger than 10% were then compared to the dip depth for free diffusion of PLL-FITC in a homogenous solution using a one-sided Student’s *t*-test. The *p*-value obtained from this test determines if the measured dip depth is significantly larger than the dip depth for free diffusion, i.e., it falls into the LLPS regime, considering the respective standard deviations and sample sizes.

### Half-FRAP model for diffusion in a circle with a semi-permeable boundary (LLPS scenario)

To consider diffusion in a circular domain surrounded by a semi-permeable boundary, which reflects the LLPS scenario with a negligible concentration outside of the condensate, we solve the two-dimensional diffusion problem with a defined flux at the boundary. The diffusion equation in polar coordinates reads

13 $$\frac{\partial c\left(r,\varphi,t\right)}{\partial t}={D}_{{{{{{\rm{app}}}}}}}{\nabla }^{2}c\left(r,\varphi,t\right).$$

Here, *c*(*r,φ,t*) is the distribution of bleached particles in a circular domain of radius *R*, and *D*_app_ is their apparent diffusion coefficient. As we do not explicitly consider interactions with binding sites or obstacles in the model and rather approximate molecular transport in the domain as a simple diffusion process, the resulting value of *D*_app_ might be smaller than the free diffusion coefficient. To accurately quantify diffusion coefficients, which is not our main goal here, other approaches should be used. The flux *J* across the boundary of the domain is set to

14 $${\left.J=-{D}_{{{{{{\rm{app}}}}}}}\frac{\partial c\left(r,\varphi,t\right)}{\partial r}\right|}_{r=R}={D}_{{{{{{\rm{app}}}}}}}{hc}\left(R,\varphi,t\right).$$

Here, the parameter *h* controls the flux and the permeability *P* = *h*·*D*_app_ at the boundary (*h* = 0: impermeable boundary; *h* > 0: (semi-)permeable boundary). The inverse of *h* can be considered the effective thickness of the boundary^22^, as shifting the concentration profile on each side of the boundary by 1/(2*h*) would heal the discontinuity of the concentration profile that is caused by the boundary. The molecular origin of the reduced flux at the boundary are the multivalent interactions among the phase-separating molecules that create an interfacial barrier (see Fig. 3g–i and Supplementary Note 1).

The condition in Eq. 14 describes the situation in which the concentration of bleached particles outside of the domain is negligible, implying that bleached particles can rapidly move away from the bleach region and get highly diluted in the surrounding medium. Accordingly, this scenario applies for a small domain surrounded by a large permeable medium, especially if transport in the surrounding medium is faster than in the domain because its viscosity is lower and/or because it contains less/weaker immobile binding sites. The diffusion equation (Eq. 13) with the respective boundary condition (Eq. 14) is solved by^48^

15 $$c(r,\varphi,t)=\mathop{\sum }\limits_{n=-\infty }^{\infty }\mathop{\sum}\limits_{{\alpha }_{n}}{U}_{n,{\alpha }_{n}}(r,\varphi ){e}^{-{D}_{{{{{{\rm{app}}}}}}}{\alpha }_{n}^{2}t},$$

where

16 $${U}_{n,{\alpha }_{n}}\left(r,\varphi \right)	=\frac{{\alpha }_{n}^{2}{J}_{n}\left({\alpha }_{n}r\right)}{\pi {R}^{2}{J}_{n}^{2}\left({\alpha }_{n}R\right)\left({\alpha }_{n}^{2}+{h}^{2}-\frac{{n}^{2}}{{R}^{2}}\right)}{\int }_{\!\!\!0}^{2\pi }d{\varphi }^{{\prime} }\cos (n(\varphi -{\varphi }^{{\prime} }))\\ 	{\int }_{\!\!\!0}^{R}{r}^{{\prime} }d{r}^{{\prime} }{c}_{0}\left({r}^{{\prime} },{\varphi }^{{\prime} }\right){J}_{n}\left({\alpha }_{n}{r}^{{\prime} }\right).$$

Here, *c*_0_(*r*′,*φ*′) is the initial distribution of bleached particles in the domain, *J*_*n*_ is the Bessel function of the first kind of order *n*, and *α*_*n*_ are the positive roots of ${J}_{n}^{{\prime} }({\alpha }_{n}r)|_{r=R}+h{J}_{n}({\alpha }_{n}R)=0$. In the case that one half of the circular domain is bleached, the initial distribution can be written as *c*_0_(*r*′,*φ*′) = 2Θ(*φ*′ − *π*), and the following expression is obtained

17 $$	{c}_{{{{{{\rm{half}}}}}}}(r,\varphi,t)=\\ 	 \mathop{\sum }\limits_{n=-\infty }^{\infty }\mathop{\sum }\limits_{{\alpha }_{n}}\frac{4{\alpha }_{n}^{2}{(\frac{{\alpha }_{n}R}{2})}^{n}{{{\rm{cos}}}}(\frac{3n\pi }{2}-n\varphi ){{{\rm{sin}}}}(\frac{n\pi }{2}){J}_{n}({\alpha }_{n}r)PF{Q}_{n}({\alpha }_{n}R){e}^{-{D}_{{{{{{\rm{app}}}}}}}{\alpha }_{n}^{2}t}}{\pi {n}^{2}(2+n)\varGamma (n){J}_{n}^{2}({\alpha }_{n}R)({\alpha }_{n}^{2}+{h}^{2}-\frac{{n}^{2}}{{R}^{2}})}.$$

In Eq. 17, the hypergeometric function *PFQ*_*n*_ (*α*_*n*_*R*) = _1_*F*_2_$(1+\frac{n}{2};1+n,2+\frac{n}{2};-\frac{{\alpha }_{n}^{2}{R}^{2}}{4})$ was used. In the case that the entire circular domain is bleached, the initial distribution can be written as *c*_0_(*r*′,*φ*′) = 1, and the following expression is obtained

18 $${c}_{{{{{{\rm{full}}}}}}}(r,t)=\mathop{\sum}\limits_{{\alpha }_{0}}\frac{2{h}^{2}{J}_{0}({\alpha }_{0}r){e}^{-{D}_{{{{{{\rm{app}}}}}}}{\alpha }_{0}^{2}t}}{{\alpha }_{0}R({\alpha }_{0}^{2}+{h}^{2}){J}_{1}({\alpha }_{0}R)}.$$

Here, the relation ${J}_{0}^{{\prime} }({\alpha }_{0}r)|_{r=R}={-{\alpha }_{0}\,J}_{1}({\alpha }_{0}R)=-h{J}_{0}({\alpha }_{0}R)$ was used (terms for *n* ≠ 0 vanish). Equations 17 and 18 yield the distribution of bleached particles after a bleach with the respective geometry has been performed. If half of a circle has been bleached, the integrated signals in the bleached (${S}_{{{{{{\rm{half}}}}}}}^{{{{{{\rm{B}}}}}}}$) and the non-bleached (${S}_{{{{{{\rm{half}}}}}}}^{{{{{{\rm{NB}}}}}}}$) semicircles read

19 $${S}_{{{{{{\rm{half}}}}}}}^{{{{{{\rm{B}}}}}}}(t)	=1-{\int }_{\!\!\!\!\!\pi }^{2\pi }{{{{{\rm{d}}}}}}\varphi {\int }_{\!\!\!0}^{R}r{{{{{\rm{d}}}}}}r\frac{{c}_{{{{{{\rm{half}}}}}}}(r,\varphi,t)}{\pi {R}^{2}}\,\\ 	=1-\mathop{\sum }\limits_{n=-\infty }^{\infty }\mathop{\sum}\limits_{{\alpha }_{n}}\frac{8{\alpha }_{n}^{2}{\left(\frac{{\alpha }_{n}R}{2}\right)}^{2n}{{{{{\rm{sin}}}}}}^{2}\left(\frac{n\pi }{2}\right)PF{Q}_{n}^{2}({\alpha }_{n}R){{{{{\rm{e}}}}}}^{-{D}_{{{{{{\rm{app}}}}}}}{\alpha }_{n}^{2}t}}{{\pi }^{2}{n}^{4}{(2+n)}^{2}\varGamma {(n)}^{2}{J}_{n}^{2}({\alpha }_{n}R)\left({\alpha }_{n}^{2}+{h}^{2}-\frac{{n}^{2}}{{R}^{2}}\right)},\\ {S}_{{{{{{\rm{half}}}}}}}^{{{{{{\rm{NB}}}}}}}(t)	=1-{\int }_{\!\!\!0}^{\pi }{{{{{\rm{d}}}}}}\varphi {\int }_{\!\!\!0}^{R}r{{{{{\rm{d}}}}}}r\frac{{c}_{{{{{{\rm{half}}}}}}}(r,\varphi,t)}{\pi {R}^{2}}\\ 	=1-\mathop{\sum }\limits_{n=-\infty }^{\infty }\mathop{\sum}\limits_{{\alpha }_{n}}\frac{8{\alpha }_{n}^{2}{\left(\frac{{\alpha }_{n}R}{2}\right)}^{2n}{{{{{\rm{sin}}}}}}^{2}\left(\frac{n\pi }{2}\right){{{{{\rm{cos}}}}}}(n\pi )PF{Q}_{n}^{2}({\alpha }_{n}R){{{{{\rm{e}}}}}}^{-{D}_{{{{{{\rm{app}}}}}}}{\alpha }_{n}^{2}t}}{{\pi }^{2}{n}^{4}{(2+n)}^{2}\varGamma {(n)}^{2}{J}_{n}^{2}({\alpha }_{n}R)\left({\alpha }_{n}^{2}+{h}^{2}-\frac{{n}^{2}}{{R}^{2}}\right)}.$$

For *n* ≠ 0, the summands with even *n* vanish, while summands with odd *n* have the same absolute value but a different sign for ${S}_{{{{{{\rm{half}}}}}}}^{{{{{{\rm{B}}}}}}}$ and ${S}_{{{{{{\rm{half}}}}}}}^{{{{{{\rm{NB}}}}}}}$ (due to the additional *cos*(*nπ*) term). These terms describe the particle exchange between the bleached and the non-bleached half of the circle. For *n* = 0, the summands for both ${S}_{{{{{{\rm{half}}}}}}}^{{{{{{\rm{B}}}}}}}$ and ${S}_{{{{{{\rm{half}}}}}}}^{{{{{{\rm{NB}}}}}}}$ reduce according to

20 $${{S}_{{{{{{\rm{half}}}}}}}^{{{{{{\rm{B}}}}}}/{{{{{\rm{NB}}}}}}}(t)|}_{n=0}=1-\frac{2}{{R}^{2}}\mathop{\sum}\limits_{{\alpha }_{0}}\frac{{h}^{2}{{{{{\rm{e}}}}}}^{-{D}_{{{{{{\rm{app}}}}}}}{\alpha }_{0}^{2}t}}{{\alpha }_{0}^{2}({\alpha }_{0}^{2}+{h}^{2})}.$$

Equation 20 describes the particle exchange across the boundary of the circle. In the case that the entire circular domain is bleached, the integrated signal in the circle is thus given by

21 $${S}_{{{{{{\rm{full}}}}}}}^{{{{{{\rm{B}}}}}}}\left(t\right)=1-{\int }_{\!\!\!0}^{2\pi }{{{{{\rm{d}}}}}}\varphi {\int }_{\!\!\!0}^{R}{r{{{{{\rm{d}}}}}}r}\frac{{c}_{{{{{{\rm{full}}}}}}}\left(r,\varphi,t\right)}{\pi {R}^{2}}=1-\frac{4}{{R}^{2}}\mathop{\sum}\limits_{{\alpha }_{0}}\frac{{h}^{2}{{{{{\rm{e}}}}}}^{-{D}_{{{{{{\rm{app}}}}}}}{\alpha }_{0}^{2}t}}{{\alpha }_{0}^{2}\left({\alpha }_{0}^{2}+{h}^{2}\right)}.$$

In Eqs. 19–21, the characteristic diffusion time ${\tau }_{{{\rm{D}}}}{=}{R}^{2}{/}({{4}}{D}_{{{\rm{app}}}})$ and the normalized time t* = *t*/*τ*_D_ = 4*D*_app_*t*/*R*^2^ can be introduced to substitute all occurrences of *D*_app_. Accordingly, when plotting the respective curves against the normalized time t*, curves for different values of the diffusion coefficient *D*_app_ fall on a master curve (Fig. 3b). Thus, the diffusion coefficient affects the scaling of the curves along the time-axis but does not affect the dip depth, which can be obtained by numerically determining the minimum of the master curve (see Fig. 3c for the resulting dip depths).

### Half-FRAP model for diffusion in a circle with a fully permeable boundary ("free diffusion" scenario)

To consider diffusion in a circular domain with a fully permeable boundary that is embedded in a larger circular domain with an impermeable boundary (such as the nuclear membrane), we solve the problem above (semi-permeable boundary) but with different boundary conditions. This scenario corresponds to molecules freely diffusing in the absence of any membrane-less structure. We replace Eq. 14 by the following equation, which sets the flux *J* across the boundary of the larger domain to zero

22 $${\left.J=-{D}_{{{{{{\rm{app}}}}}}}\frac{\partial c\left(r,\varphi,t\right)}{\partial r}\right |}_{r={R}_{{{\rm{L}}}}}=0.$$

Here, *R*_L_ is the radius of the large circular domain. The diffusion equation (Eq. 13) with the respective boundary condition (Eq. 22) is solved by

23 $$c(r,\varphi,t)=\mathop{\sum }\limits_{n=-\infty }^{\infty }\mathop{\sum}\limits_{{\alpha }_{n}}{U}_{n,{\alpha }_{n}}(r,\varphi ){{{{{\rm{e}}}}}}^{-{D}_{{{{{{\rm{app}}}}}}}{\alpha }_{n}^{2}t},$$

where

24 $${U}_{n,{\alpha }_{n} > 0}\left(r,\varphi \right)	=\frac{{\alpha }_{n}^{2}{J}_{n}\left({\alpha }_{n}r\right)}{\pi {R}_{{{\rm{L}}}}^{2}{J}_{n}^{2}\left({\alpha }_{n}{R}_{{{\rm{L}}}}\right)\left({\alpha }_{n}^{2}-\frac{{n}^{2}}{{R}_{{{\rm{L}}}}^{2}}\right)}{\int }_{\!\!\!0}^{2\pi }{{{{{\rm{d}}}}}}{\varphi }^{{\prime} }\cos (n(\varphi -{\varphi }^{{\prime} }))\\ 	{\int }_{\!\!\!0}^{{R}_{{{\rm{L}}}}}{r}^{{\prime} }{{{{{\rm{d}}}}}}{r}^{{\prime} }{c}_{0}\left({r}^{{\prime} },{\varphi }^{{\prime} }\right){J}_{n}\left({\alpha }_{n}{r}^{{\prime} }\right),\,{U}_{0,{\alpha }_{0}=0}\left(r,\varphi \right)=\frac{{R}_{{{\rm{C}}}}^{2}}{{R}_{{{\rm{L}}}}^{2}}.$$

Here, ${c}_{0}\left({r}^{{\prime} },{\varphi }^{{\prime} }\right)$ is the initial distribution of bleached particles, *J*_*n*_ is the Bessel function of the first kind of order *n*, and *α*_*n*_ are the positive roots of ${J}_{n}^{{\prime} }({\alpha }_{n}r)|_{r=R}=0$. In the case that one half of the smaller circular domain with radius *R*_C_ is bleached, the initial distribution can be written as ${c}_{0}\left({r}^{{\prime} },{\varphi }^{{\prime} }\right)=2\varTheta \left({\varphi }^{{\prime} }-\pi \right)\varTheta \left({R}_{{{\rm{C}}}}-{r}^{{\prime} }\right)$, and the following expression is obtained

25 $$	{c}_{{{{{{\rm{half}}}}}}}(r,\varphi,t)=\frac{{R}_{{{\rm{C}}}}^{2}}{{R}_{{{\rm{L}}}}^{2}}\\ 	+\mathop{\sum }\limits_{n=-\infty }^{\infty }\mathop{\sum}\limits_{{\alpha }_{n} > 0}\frac{4{R}_{{{\rm{C}}}}^{2}{\alpha }_{n}^{2}{\left(\frac{{\alpha }_{n}{R}_{{{\rm{C}}}}}{2}\right)}^{n}{{{{{\rm{cos}}}}}}\left(\frac{3n\pi }{2}-n\varphi \right){{{{{\rm{sin}}}}}}\left(\frac{n\pi }{2}\right){J}_{n}({\alpha }_{n}r)PF{Q}_{n}({\alpha }_{n}{R}_{{{\rm{C}}}}){{{{{\rm{e}}}}}}^{-{D}_{{{{{{\rm{app}}}}}}}{\alpha }_{n}^{2}t}}{\pi {R}_{{{\rm{L}}}}^{2}{n}^{2}(2+n)\varGamma (n){J}_{n}^{2}({\alpha }_{n}{R}_{{{\rm{L}}}})\left({\alpha }_{n}^{2}-\frac{{n}^{2}}{{R}_{{{\rm{L}}}}^{2}}\right)}.$$

In Eq. 25, the hypergeometric function $PF{Q}_{n}({\alpha }_{n}{R}_{{{\rm{C}}}})={{}_{1}F}_{2}(1+\frac{n}{2};1+n,2+\frac{n}{2};-\frac{{\alpha }_{n}^{2}{R}_{{{\rm{C}}}}^{2}}{4})$ was used. In the case that the entire circular domain with radius *R*_C_ is bleached, the initial distribution can be written as ${c}_{0}\left({r}^{{\prime} },{\varphi }^{{\prime} }\right)=\varTheta \left({R}_{{{\rm{C}}}}-{r}^{{\prime} }\right)$, and the following expression is obtained

26 $${c}_{{{{{{\rm{full}}}}}}}\left(r,t\right)=\frac{{R}_{{{\rm{C}}}}^{2}}{{R}_{{{\rm{L}}}}^{2}}+\mathop{\sum }\limits_{{\alpha }_{0} > 0}\frac{2{R}_{{{\rm{C}}}}{J}_{0}\left({\alpha }_{0}r\right){J}_{1}\left({\alpha }_{0}{R}_{{{\rm{C}}}}\right){{{{{\rm{e}}}}}}^{-{D}_{{{{{{\rm{app}}}}}}}{\alpha }_{0}^{2}t}}{{\alpha }_{0}{R}_{{{\rm{L}}}}^{2}{J}_{0}^{2}\left({\alpha }_{0}{R}_{{{\rm{L}}}}\right)}.$$

Equations 25 and 26 yield the distribution of bleached particles after a bleach with the respective geometry has been performed. If half of a circle has been bleached, the integrated signals in the bleached (${S}_{{{{{{\rm{half}}}}}}}^{{{{{{\rm{B}}}}}}}$) and the non-bleached (${S}_{{{{{{\rm{half}}}}}}}^{{{{{{\rm{NB}}}}}}}$) semicircles read

27 $$	{S}_{{{{{{\rm{half}}}}}}}^{{{{{{\rm{B}}}}}}}(t)=1-{\int }_{\!\!\!\!\pi }^{2\pi }{{{{{\rm{d}}}}}}\varphi {\int }_{\!\!\!0}^{{R}_{{{\rm{C}}}}}r{{{{{\rm{d}}}}}}r\frac{{c}_{{{{{{\rm{half}}}}}}}(r,\varphi,t)}{\pi {R}_{{{\rm{C}}}}^{2}}=1-\frac{{R}_{{{\rm{C}}}}^{2}}{2{R}_{{{\rm{L}}}}^{2}}\\ 	\qquad\qquad-\mathop{\sum }\limits_{n=-\infty }^{\infty }\mathop{\sum}\limits_{{\alpha }_{n} > 0}\frac{8{R}_{{{\rm{C}}}}^{2}{\alpha }_{n}^{2}{\left(\frac{{\alpha }_{n}{R}_{{{\rm{C}}}}}{2}\right)}^{2n}{{{{{\rm{sin}}}}}}^{2}\left(\frac{n\pi }{2}\right)PF{Q}_{n}^{2}({\alpha }_{n}{R}_{{{\rm{C}}}}){{{{{\rm{e}}}}}}^{-{D}_{{{\rm{app}}}}{\alpha }_{n}^{2}t}}{{\pi }^{2}{R}_{{{\rm{L}}}}^{2}{n}^{4}{(2+n)}^{2}\varGamma {(n)}^{2}{J}_{n}^{2}({\alpha }_{n}{R}_{{{\rm{L}}}})\left({\alpha }_{n}^{2}-\frac{{n}^{2}}{{R}_{{{\rm{L}}}}^{2}}\right)},\,\\ 	{S}_{{{{{{\rm{half}}}}}}}^{{{{{{\rm{NB}}}}}}}(t)=1-{\int }_{\!\!\!0}^{\pi }{{{{{\rm{d}}}}}}\varphi {\int }_{\!\!\!0}^{{R}_{{{\rm{C}}}}}r{{{{{\rm{d}}}}}}r\frac{{c}_{{{{{{\rm{half}}}}}}}(r,\varphi,t)}{\pi {R}_{{{\rm{C}}}}^{2}}=1-\frac{{R}_{{{\rm{C}}}}^{2}}{2{R}_{{{\rm{L}}}}^{2}}\\ 	 \qquad\qquad-\mathop{\sum }\limits_{n=-\infty }^{\infty }\mathop{\sum}\limits_{{\alpha }_{n} > 0}\frac{8{R}_{{{\rm{C}}}}^{2}{\alpha }_{n}^{2}{\left(\frac{{\alpha }_{n}{R}_{{{\rm{C}}}}}{2}\right)}^{2n}{{{{{\rm{sin}}}}}}^{2}\left(\frac{n\pi }{2}\right){{{{{\rm{cos}}}}}}(n\pi )PF{Q}_{n}^{2}({\alpha }_{n}{R}_{{{\rm{C}}}}){{{{{\rm{e}}}}}}^{-{D}_{{{{{{\rm{app}}}}}}}{\alpha }_{n}^{2}t}}{{\pi }^{2}{R}_{{{\rm{L}}}}^{2}{n}^{4}{(2+n)}^{2}\varGamma {(n)}^{2}{J}_{n}^{2}({\alpha }_{n}{R}_{{{\rm{L}}}})\left({\alpha }_{n}^{2}-\frac{{n}^{2}}{{R}_{{{\rm{L}}}}^{2}}\right)}.$$

For *n* = 0, the summands for ${S}_{{{{{{\rm{half}}}}}}}^{{{{{{\rm{B}}}}}}}$ and ${S}_{{{{{{\rm{half}}}}}}}^{{{{{{\rm{NB}}}}}}}$ reduce according to

28 $${\left.{S}_{{{{{{\rm{half}}}}}}}^{{{{{{\rm{B}}}}}}/{{{{{\rm{NB}}}}}}}\left(t\right)\right | }_{n=0}=1-\frac{{R}_{{{\rm{C}}}}^{2}}{{2R}_{{{\rm{L}}}}^{2}}-\frac{2}{{R}_{{{\rm{L}}}}^{2}}\mathop{\sum}\limits_{{\alpha }_{0} > 0}\frac{{{J}_{1}^{2}\left({\alpha }_{0}{R}_{{{\rm{C}}}}\right){{{{{\rm{e}}}}}}}^{-{D}_{{{{{{\rm{app}}}}}}}{\alpha }_{0}^{2}t}}{{\alpha }_{0}^{2}{J}_{0}^{2}\left({\alpha }_{0}{R}_{{{\rm{L}}}}\right)}.$$

In the case that the entire circular domain is bleached, the integrated signal in the circle is given by

29 $${S}_{{{{{{\rm{full}}}}}}}^{{{{{{\rm{B}}}}}}}\left(t\right)=1-{\int }_{\!\!\!0}^{2\pi }{{{{{\rm{d}}}}}}\varphi {\int }_{\!\!\!0}^{{R}_{{{\rm{C}}}}}{r{{{{{\rm{d}}}}}}r}\frac{{c}_{{{{{{\rm{full}}}}}}}\left(r,\varphi,t\right)}{\pi {R}_{{{\rm{C}}}}^{2}}=1-\frac{{R}_{{{\rm{C}}}}^{2}}{{R}_{{{\rm{L}}}}^{2}}-\frac{4}{{R}_{{{\rm{L}}}}^{2}}\mathop{\sum }\limits_{{\alpha }_{0} > 0}\frac{{J}_{1}^{2}\left({\alpha }_{0}{R}_{{{\rm{C}}}}\right){{{{{\rm{e}}}}}}^{-{D}_{{{{{{\rm{app}}}}}}}{\alpha }_{0}^{2}t}}{{\alpha }_{0}^{2}{J}_{0}^{2}\left({\alpha }_{0}{R}_{{{\rm{L}}}}\right)}.$$

Equation 29 corresponds to the result published previously by Mueller and colleagues^49^.

Similar to the LLPS scenario above (Eqs. 19–21), all occurrences of *D*_app_ in Eqs. 27–29 can be substituted using the normalized time t* = *t*/*τ*_D_ = 4*D*_app_*t*/*R*^2^. Accordingly, curves for different values of the diffusion coefficient fall on a master curve, which means that the diffusion coefficient affects the scaling of the curves along the time-axis but does not affect the dip depth.

### Half-FRAP model for a reaction-diffusion process in a circle with a fully permeable boundary (ICBS scenario)

To consider a reaction–diffusion process with immobile binding sites in a circular domain with a fully permeable boundary, which reflects the ICBS scenario, we extend the scenario for free diffusion in a circle with a fully permeable boundary above and solve the following set of equations

30 $$\frac{\partial f(r,\varphi,t)}{\partial t}={D}_{{{{{{\rm{app}}}}}}}{\nabla }^{2}f(r,\varphi,t)-{k}_{{{{{{\rm{on}}}}}}}^{\ast }f+{k}_{{{{{{\rm{off}}}}}}}c,\,\frac{\partial c(r,\varphi,t)}{\partial t}={k}_{{{{{{\rm{on}}}}}}}^{\ast }f-{k}_{{{{{{\rm{off}}}}}}}c.$$

In Eq. 30, ${k}_{{{{{{\rm{on}}}}}}}^{*}$ and *k*_off_ are the pseudo-association and dissociation rates, respectively, and *f*(*r,φ,t*) and *c*(*r,φ,t*) are the distributions of free and bound bleached particles, respectively. The other parameters are introduced in the section above. Similar to Mueller and colleagues^49^, we construct a solution of the form

31 $$\begin{array}{c}f(r,\varphi,t)=\mathop{\sum }\limits_{n=-\infty }^{\infty }\mathop{\sum}\limits_{{\alpha }_{n}}({U}_{n,{\alpha }_{n}}(r,\varphi )+{W}_{n,{\alpha }_{n}}(r,\varphi )){{{{{\rm{e}}}}}}^{-({w}_{{\alpha }_{n}}+{v}_{{\alpha }_{n}})t},\\ c(r,\varphi,t)=\mathop{\sum }\limits_{n=-\infty }^{\infty }\mathop{\sum}\limits_{{\alpha }_{n}}({V}_{n,{\alpha }_{n}}(r,\varphi )+{X}_{n,{\alpha }_{n}}(r,\varphi )){{{{{\rm{e}}}}}}^{-({w}_{{\alpha }_{n}}-{v}_{{\alpha }_{n}})t},\end{array}$$

where

32 $${w}_{{\alpha }_{n}}=\frac{1}{2}\left({D}_{{{{{{\rm{app}}}}}}}{\alpha }_{n}^{2}+{k}_{{{{{{\rm{on}}}}}}}^{*}+{k}_{{{{{{\rm{off}}}}}}}\right),\;{v}_{{\alpha }_{n}}=\sqrt{\frac{1}{4}{\left({D}_{{{{{{\rm{app}}}}}}}{\alpha }_{n}^{2}+{k}_{{{{{{\rm{on}}}}}}}^{*}+{k}_{{{{{{\rm{off}}}}}}}\right)}^{2}-{k}_{{{{{{\rm{off}}}}}}}{D}_{{{{{{\rm{app}}}}}}}{\alpha }_{n}^{2}}.$$

With the initial distribution of bleached particles being ${f}_{0}\left({r}^{{\prime} },{\varphi }^{{\prime} }\right)$ and ${c}_{0}\left({r}^{{\prime} },{\varphi }^{{\prime} }\right)={\frac{{k}_{{{{{{\rm{on}}}}}}}^{*}}{{k}_{{{{{{\rm{off}}}}}}}}f}_{0}\left({r}^{{\prime} },{\varphi }^{{\prime} }\right)$, the coefficients in Eq. 31 are given by

33 $${U}_{n,{\alpha }_{n} > 0}\left(r,\varphi \right)=	\frac{\left({k}_{{{{{{\rm{off}}}}}}}-{v}_{{\alpha }_{n}}-{w}_{{\alpha }_{n}}\right)\left({v}_{{\alpha }_{n}}-{w}_{{\alpha }_{n}}\right)}{2{k}_{{{{{{\rm{off}}}}}}}{v}_{{\alpha }_{n}}}{Z}_{n,{\alpha }_{n} > 0}\left(r,\varphi \right),\\ {U}_{0,{\alpha }_{0}=0}\left(r,\varphi \right)=	0,\\ {V}_{n,{\alpha }_{n} > 0}\left(r,\varphi \right)=	\frac{\left({k}_{{{{{{\rm{off}}}}}}}+{v}_{{\alpha }_{n}}-{w}_{{\alpha }_{n}}\right)\left({v}_{{\alpha }_{n}}+{w}_{{\alpha }_{n}}\right)}{2{k}_{{{{{{\rm{off}}}}}}}{v}_{{\alpha }_{n}}}{Z}_{n,{\alpha }_{n} > 0}\left(r,\varphi \right),\\ {V}_{0,{\alpha }_{0}=0}\left(r,\varphi \right)=	\frac{1}{\pi {R}_{{{\rm{L}}}}^{2}}{\int }_{\!\!\!0}^{2\pi }{{{{{\rm{d}}}}}}{\varphi }^{{\prime} }{\int }_{\!\!\!0}^{{R}_{{{\rm{L}}}}}{r}^{{\prime} }{{{{{\rm{d}}}}}}{r}^{{\prime} }{f}_{0}\left({r}^{{\prime} },{\varphi }^{{\prime} }\right){J}_{0}\left({\alpha }_{0}{r}^{{\prime} }\right),\\ {{W}_{n,{\alpha }_{n}}\left(r,\varphi \right)}=	\frac{{k}_{{{{{{\rm{on}}}}}}}^{*}\left({v}_{{\alpha }_{n}}-{w}_{{\alpha }_{n}}\right)}{2{k}_{{{{{{\rm{off}}}}}}}{v}_{{\alpha }_{n}}}{Z}_{n,{\alpha }_{n} > 0}\left(r,\varphi \right),\\ {{X}_{n,{\alpha }_{n}}\left(r,\varphi \right)}=	\frac{{k}_{{{{{{\rm{on}}}}}}}^{*}\left({v}_{{\alpha }_{n}}+{w}_{{\alpha }_{n}}\right)}{2{k}_{{{{{{\rm{off}}}}}}}{v}_{{\alpha }_{n}}}{Z}_{n,{\alpha }_{n} > 0}\left(r,\varphi \right),$$

where

34 $${Z}_{n,{\alpha }_{n} > 0}\left(r,\varphi \right)	=\frac{{\alpha }_{n}^{2}\;{J}_{n}\left({\alpha }_{n}r\right)}{\pi {R}_{{{\rm{L}}}}^{2}\;{J}_{n}^{2}\left({\alpha }_{n}{R}_{{{\rm{L}}}}\right)\left({\alpha }_{n}^{2}-\frac{{n}^{2}}{{R}_{{{\rm{L}}}}^{2}}\right)}{\int }_{\!\!\!0}^{2\pi }{{{{{\rm{d}}}}}}{\varphi }^{{\prime} }\cos \left(n\left(\varphi -{\varphi }^{{\prime} }\right)\right)\\ 	{\int }_{\!\!\!0}^{{R}_{{{\rm{L}}}}}{r}^{{\prime} }{{{{{\rm{d}}}}}}{r}^{{\prime} }{f}_{0}\left({r}^{{\prime} },{\varphi }^{{\prime} }\right)\,{J}_{n}\left({\alpha }_{n}{r}^{{\prime} }\right).$$

In the case that one half of the smaller circular domain with radius *R*_C_ is bleached, the initial distribution can be written as ${f}_{0}\left(r^{\prime},\varphi ^{\prime} \right)=2{F}_{{{\rm{eq}}}}\varTheta \left({\varphi }^{{\prime} }-\pi \right)\varTheta \left({R}_{{{\rm{C}}}}-{r}^{{\prime} }\right)$, and the following expressions are obtained

35 $${f}_{{{{{{\rm{half}}}}}}}(r,\varphi,t)	=\mathop{\sum }\limits_{n=-\infty }^{\infty }\mathop{\sum}\limits_{{\alpha }_{n} > 0}\frac{\left({k}_{{{{{{\rm{off}}}}}}}+{k}_{{{{{{\rm{on}}}}}}}^{\ast }-{v}_{{\alpha }_{n}}-{w}_{{\alpha }_{n}}\right)\left({v}_{{\alpha }_{n}}-{w}_{{\alpha }_{n}}\right)}{2{k}_{{{{{{\rm{off}}}}}}}{v}_{{\alpha }_{n}}}\\ 	\qquad{Z}_{n,{\alpha }_{n} > 0}^{{{{{{\rm{half}}}}}}}(r,\varphi ){{{{{\rm{e}}}}}}^{-({w}_{{\alpha }_{n}}+{v}_{{\alpha }_{n}})t},{c}_{{{{{{\rm{half}}}}}}}(r,\varphi,t)={F}_{{{{{{\rm{eq}}}}}}}\frac{{R}_{{{\rm{C}}}}^{2}}{2{R}_{{{\rm{L}}}}^{2}}\\ 	\quad+\mathop{\sum }\limits_{n=-\infty }^{\infty }\mathop{\sum}\limits_{{\alpha }_{n} > 0}\frac{\left({k}_{{{{{{\rm{off}}}}}}}+{k}_{{{{{{\rm{on}}}}}}}^{\ast }+{v}_{{\alpha }_{n}}-{w}_{{\alpha }_{n}}\right)\left({v}_{{\alpha }_{n}}+{w}_{{\alpha }_{n}}\right)}{2{k}_{{{{{{\rm{off}}}}}}}{v}_{{\alpha }_{n}}}\\ 	\qquad{Z}_{n,{\alpha }_{n} > 0}^{{{{{{\rm{half}}}}}}}(r,\varphi ){{{{{\rm{e}}}}}}^{-({w}_{{\alpha }_{n}}-{v}_{{\alpha }_{n}})t},\\ {Z}_{n,{\alpha }_{n} > 0}^{{{{{{\rm{half}}}}}}}(r,\varphi )	=\frac{4{F}_{{{{{{\rm{eq}}}}}}}{R}_{{{\rm{C}}}}^{2}{\alpha }_{n}^{2}{\left(\frac{{\alpha }_{n}{R}_{{{\rm{C}}}}}{2}\right)}^{n}\cos\left(\frac{3n\pi }{2}-n\varphi \right)\sin\left(\frac{n\pi }{2}\right){J}_{n}({\alpha }_{n}r)PF{Q}_{n}({\alpha }_{n}{R}_{{{\rm{C}}}})}{\pi {R}_{{{\rm{L}}}}^{2}{n}^{2}(2+n)\varGamma (n){J}_{n}^{2}({\alpha }_{n}{R}_{{{\rm{L}}}})\left({\alpha }_{n}^{2}-\frac{{n}^{2}}{{R}_{{{\rm{L}}}}^{2}}\right)},\\ {F}_{{{{{{\rm{eq}}}}}}}	=\frac{{k}_{{{{{{\rm{off}}}}}}}}{{k}_{{{{{{\rm{on}}}}}}}^{\ast }+{k}_{{{{{{\rm{off}}}}}}}}.$$

In the case that the entire circular domain with radius *R*_C_ is bleached, the initial distribution can be written as ${f}_{0}\left(r^{{\prime}},\varphi^{{\prime}} \right)={F}_{{{{{{\rm{eq}}}}}}}\varTheta \left({R}_{C}-{r}^{{\prime} }\right)$, and the following expressions are obtained

36 $${f}_{{{{{{\rm{full}}}}}}}(r,\varphi,t)	=\mathop{\sum}\limits_{{\alpha }_{0} > 0}\frac{\left({k}_{{{{{{\rm{off}}}}}}}+{k}_{{{{{{\rm{on}}}}}}}^{\ast }-{v}_{{\alpha }_{0}}-{w}_{{\alpha }_{0}}\right)\left({v}_{{\alpha }_{0}}-{w}_{{\alpha }_{0}}\right)}{2{k}_{{{{{{\rm{off}}}}}}}{v}_{{\alpha }_{0}}}{Z}_{{\alpha }_{0} > 0}^{{{{{{\rm{full}}}}}}}(r,\varphi ){{{{{\rm{e}}}}}}^{-({w}_{{\alpha }_{0}}+{v}_{{\alpha }_{0}})t},\\ {c}_{{{{{{\rm{full}}}}}}}(r,\varphi,t)	={F}_{{{{{{\rm{eq}}}}}}}\frac{{R}_{{{\rm{C}}}}^{2}}{{R}_{{{\rm{L}}}}^{2}}\\ 	\quad+ \mathop{\sum}\limits_{{\alpha }_{0} > 0}\frac{\left({k}_{{{{{{\rm{off}}}}}}}+{k}_{{{{{{\rm{on}}}}}}}^{\ast }+{v}_{{\alpha }_{0}}-{w}_{{\alpha }_{0}}\right)\left({v}_{{\alpha }_{0}}+{w}_{{\alpha }_{0}}\right)}{2{k}_{{{\rm{off}}}}{v}_{{\alpha }_{0}}}{Z}_{{\alpha }_{0} > 0}^{{{{{{\rm{full}}}}}}}(r,\varphi ){{{{{\rm{e}}}}}}^{-({w}_{{\alpha }_{0}}-{v}_{{\alpha }_{0}})t},\\ {Z}_{{\alpha }_{0} > 0}^{{{{{{\rm{full}}}}}}}(r,\varphi )	=\frac{2{F}_{{{{{{\rm{eq}}}}}}}{R}_{{{\rm{C}}}}{J}_{0}({\alpha }_{0}r){J}_{1}({\alpha }_{0}{R}_{{{\rm{C}}}})}{{\alpha }_{0}{R}_{{{\rm{L}}}}^{2}{J}_{0}^{2}({\alpha }_{0}{R}_{{{\rm{L}}}})}.$$

Equations 35 and 36 yield the distribution of bleached particles at time *t* after a bleach with the respective geometry has been performed. If half of a circle has been bleached, the integrated signals in the bleached (${S}_{{{{{{\rm{half}}}}}}}^{{{{{{\rm{B}}}}}}}$) and the non-bleached (${S}_{{{{{{\rm{half}}}}}}}^{{{{{{\rm{NB}}}}}}}$) semicircles are given by

37 $${S}_{{{{{{\rm{half}}}}}}}^{{{{{{\rm{B}}}}}}}(t)=	1-{\int }_{\!\!\!\!\pi }^{2\pi }{{{{{\rm{d}}}}}}\varphi {\int }_{\!\!\!\!0}^{{R}_{{{\rm{C}}}}}r{{{{{\rm{d}}}}}}r\frac{{c}_{{{{{{\rm{half}}}}}}}(r,\varphi,t)+{f}_{{{{{{\rm{half}}}}}}}(r,\varphi,t)}{\pi {R}_{{{\rm{C}}}}^{2}}=1-\frac{{R}_{{{\rm{C}}}}^{2}}{2{R}_{{{\rm{L}}}}^{2}}\\ 	 -\mathop{\sum }\limits_{n=-\infty }^{\infty }\mathop{\sum}\limits_{{\alpha }_{n} > 0}[A{{{{{\rm{e}}}}}}^{-({w}_{{\alpha }_{n}}+{v}_{{\alpha }_{n}})t}+B{{{{{\rm{e}}}}}}^{-({w}_{{\alpha }_{n}}-{v}_{{\alpha }_{n}})t}]{Z}_{n,{\alpha }_{n} > 0}^{{{\rm{B}}}}(r,\varphi ),\\ {S}_{{{{{{\rm{half}}}}}}}^{{{{{{\rm{NB}}}}}}}(t)=	1-{\int }_{\!\!\!\!0}^{\pi }{{{{{\rm{d}}}}}}\varphi {\int }_{\!\!\!\!0}^{{R}_{{{\rm{C}}}}}r{{{{{\rm{d}}}}}}r\frac{{c}_{{{{{{\rm{half}}}}}}}(r,\varphi,t)+{f}_{{{{{{\rm{half}}}}}}}(r,\varphi,t)}{\pi {R}_{{{\rm{C}}}}^{2}}=1-\frac{{R}_{{{\rm{C}}}}^{2}}{2{R}_{{{\rm{L}}}}^{2}}\\ 	 -\mathop{\sum }\limits_{n=-\infty }^{\infty }\mathop{\sum}\limits_{{\alpha }_{n} > 0}[A{{{{{\rm{e}}}}}}^{-({w}_{{\alpha }_{n}}+{v}_{{\alpha }_{n}})t}+B{{{{{\rm{e}}}}}}^{-({w}_{{\alpha }_{n}}-{v}_{{\alpha }_{n}})t}]{Z}_{n,{\alpha }_{n} > 0}^{{{\rm{NB}}}}(r,\varphi ),$$

where

38 $${Z}_{n,{\alpha }_{n} > 0}^{{{{{{\rm{B}}}}}}}\left(r,\varphi \right)	=\frac{{8{{F}_{{{{{{\rm{eq}}}}}}}R}_{{{\rm{C}}}}^{2}\alpha }_{n}^{2}{\left(\frac{{\alpha }_{n}{R}_{{{\rm{C}}}}}{2}\right)}^{2n}{\sin }^{2}\left(\frac{n\pi }{2}\right){{PFQ}}_{n}^{2}\left({\alpha }_{n}{R}_{{{\rm{C}}}}\right)}{{\pi }^{2}{R}_{{{\rm{L}}}}^{2}{n}^{4}{\left(2+n\right)}^{2}{\varGamma \left(n\right)}^{2}{J}_{n}^{2}\left({\alpha }_{n}{R}_{{{\rm{L}}}}\right)\left({\alpha }_{n}^{2}-\frac{{n}^{2}}{{R}_{{{\rm{L}}}}^{2}}\right)},\\ {Z}_{n,{\alpha }_{n} > 0}^{{{{{{\rm{NB}}}}}}} \left(r,\varphi \right)	=\frac{{8{F}_{{{{{{\rm{eq}}}}}}}{R}_{{{\rm{C}}}}^{2}\alpha }_{n}^{2}{\left(\frac{{\alpha }_{n}{R}_{{{\rm{C}}}}}{2}\right)}^{2n}{\sin }^{2}\left(\frac{n\pi }{2}\right)\cos \left(n\pi \right){{PFQ}}_{n}^{2}\left({\alpha }_{n}{R}_{{{\rm{C}}}}\right)}{{\pi }^{2}{R}_{{{\rm{L}}}}^{2}{n}^{4}{\left(2+n\right)}^{2}{\varGamma \left(n\right)}^{2}{J}_{n}^{2}\left({\alpha }_{n}{R}_{{{\rm{L}}}}\right)\left({\alpha }_{n}^{2}-\frac{{n}^{2}}{{R}_{{{\rm{L}}}}^{2}}\right)},\\ A	=\frac{\left({k}_{{{{{{\rm{off}}}}}}}+{k}_{{{{{{\rm{on}}}}}}}^{*}-{v}_{{\alpha }_{n}}-{w}_{{\alpha }_{n}}\right)\left({v}_{{\alpha }_{n}}-{w}_{{\alpha }_{n}}\right)}{2{k}_{{{{{{\rm{off}}}}}}}{v}_{{\alpha }_{n}}},\\ B	=\frac{\left({k}_{{{{{{\rm{off}}}}}}}+{k}_{{{{{{\rm{on}}}}}}}^{*}+{v}_{{\alpha }_{n}}-{w}_{{\alpha }_{n}}\right)\left({v}_{{\alpha }_{n}}+{w}_{{\alpha }_{n}}\right)}{2{k}_{{{{{{\rm{off}}}}}}}{v}_{{\alpha }_{n}}}.$$

For *n* = 0, the summands for ${S}_{{{{{{\rm{half}}}}}}}^{{{{{{\rm{B}}}}}}}$ and ${S}_{{{{{{\rm{half}}}}}}}^{{{{{{\rm{NB}}}}}}}$ reduce according to

39 $${{S}_{{{{{{\rm{half}}}}}}}^{{{{{{\rm{B}}}}}}/{{{{{\rm{NB}}}}}}}(t)|}_{n=0}=1-\frac{{R}_{{{\rm{C}}}}^{2}}{2{R}_{{{\rm{L}}}}^{2}}-\frac{2{F}_{{{{{{\rm{eq}}}}}}}}{{R}_{{{\rm{L}}}}^{2}}\mathop{\sum}\limits_{{\alpha }_{0} > 0}\frac{{J}_{1}^{2}({\alpha }_{0}{R}_{{{\rm{C}}}})}{{\alpha }_{0}^{2}{J}_{0}^{2}({\alpha }_{0}{R}_{{{\rm{L}}}})}\left[A{{{{{{\rm{e}}}}}}}^{-({w}_{{\alpha }_{0}}+{v}_{{\alpha }_{0}})t}+B{{{{{{\rm{e}}}}}}}^{-({w}_{{\alpha }_{0}}-{v}_{{\alpha }_{0}})t}\right].$$

In the case that the entire circular domain is bleached, the integrated signal in the circle is given by

40 $${S}_{{{{{{\rm{full}}}}}}}^{{{{{{\rm{B}}}}}}}(t)	=1-{\int }_{\!\!\!0}^{2\pi }{{{{{\rm{d}}}}}}\varphi {\int }_{\!\!\!0}^{{R}_{{{\rm{C}}}}}r{{{{{\rm{d}}}}}}r\frac{{c}_{{{{{{\rm{full}}}}}}}(r,\varphi,t)+{f}_{{{{{{\rm{full}}}}}}}(r,\varphi,t)}{\pi {R}_{{{\rm{C}}}}^{2}}\\ 	=1-\frac{{R}_{{{\rm{C}}}}^{2}}{{R}_{{{\rm{L}}}}^{2}}-\frac{4{F}_{{{{{{\rm{eq}}}}}}}}{{R}_{{{\rm{L}}}}^{2}}\mathop{\sum}\limits_{{\alpha }_{0} > 0}\frac{{J}_{1}^{2}({\alpha }_{0}{R}_{{{\rm{C}}}})}{{\alpha }_{0}^{2}{J}_{0}^{2}({\alpha }_{0}{R}_{{{\rm{L}}}})}\left[A{{{{{\rm{e}}}}}}^{-({w}_{{\alpha }_{0}}+{v}_{{\alpha }_{0}})t}+B{{{{{\rm{e}}}}}}^{-({w}_{{\alpha }_{0}}-{v}_{{\alpha }_{0}})t}\right].$$

Eq. 40 corresponds to the result published previously by Mueller and colleagues^49^.

As opposed to the equations above that describe LLPS (Eqs. 19–21) or free diffusion (Eqs. 27–29), there is no straightforward way to remove the dependence of the equations for ICBS on the diffusion coefficient *D*_app_. However, as discussed by Mueller and colleagues^49^, the reaction-diffusion model solved by Eqs. 37–40 converges to simpler models for several limiting cases, i.e., a pure diffusion model for ${k}_{{{{{{\rm{on}}}}}}}^{\ast }/{k}_{{{{{{\rm{off}}}}}}}\ll 1$, an effective diffusion model for ${k}_{{{{{{\rm{on}}}}}}}^{\ast }{R}^{2}/{D}_{{{{{{\rm{app}}}}}}}\gg 1$, and a reaction-dominant model for ${k}_{{{{{{\rm{on}}}}}}}^{\ast }{R}^{2}/{D}_{{{{{{\rm{app}}}}}}}\ll 1$ and ${k}_{{{{{{\rm{off}}}}}}}/{k}_{{{{{{\rm{on}}}}}}}^{\ast }\lesssim 1$. In these limiting cases, normalized times can be introduced to make the equations independent of the diffusion coefficient (Fig. 3e). For the pure diffusion model and the effective diffusion model, the dip depths are equal to that for free diffusion in a domain with a fully permeable boundary. For the reaction-dominant model, the dip depth depends on the binding strength ${k}_{{{{{{\rm{on}}}}}}}^{*}/{k}_{{{{{{\rm{off}}}}}}}$, i.e., the dip vanishes for large binding strengths and converges to the value obtained for free diffusion for small binding strengths. Numerical evaluation of the dip depth for parameter combinations that do not correspond to these limiting cases shows that dip depths that lie between the values for the limiting cases are obtained (Fig. 3f, gray area).

### Statistics and reproducibility

The microscopy images presented in Fig. 1c, d and Supplementary Fig. 1, are representative images of a pool of at least 12 images.

All the 1,6-HD experiments in Fig. 1e are presented as dot plots / box plots (with the upper and lower bounds of the box corresponding to the first and third quartiles, and the line in the center to the median of the data), using data from 350 (PLL-HA, LLPS), 91 (PLL, ICBS), 143 (CD, in living cells) or 288 (DDX4, in living cells) structures of interest in 18 (DDX4) or 8 (CD) cells.

The partial-FRAP curves presented in Fig. 1g, i are the means of 16 (PLL-HA, 0 mM MgCl_2_), 16 (PLL-HA, 25 mM MgCl_2_), 34 (PLL-HA, 50 mM MgCl_2_), 24 (PLL-HA, 100 mM MgCl_2_), 19 (PLL-HA, 150 mM MgCl_2_), 7 (PLL, ICBS), 10 (CD*-YFP, in living cells) or 13 (DDX4, in living cells) independent experiments, +/−SEM.

The full-FRAP curves presented in Fig. 1f, h are the means of 6 (PLL-HA, 0 mM MgCl_2_), 6 (PLL-HA, 25 mM MgCl_2_), 13 (PLL-HA, 50 mM MgCl_2_), 7 (PLL-HA, 100 mM MgCl_2_), 10 (PLL-HA, 150 mM MgCl_2_), 7 (PLL, ICBS), 10 (CD*-YFP, in living cells) or 12 (DDX4, in living cells) independent experiments, +/−SEM.

The half-FRAP curves presented in Fig. 2a–c, g–i, and Supplementary Figs. 2–5, 8 are the means of 16 (PLL-HA, 0 mM MgCl_2_), 16 (PLL-HA, 25 mM MgCl_2_), 34 (PLL-HA, 50 mM MgCl_2_), 24 (PLL-HA, 100 mM MgCl_2_), 19 (PLL-HA, 150 mM MgCl_2_), 7 (PLL, ICBS), 6 (PLL-HA gels), 7 (PLL in solution), 15 (PEG-Rhodamine/dextran), 7 (DDX4, in vitro), 10 (CD*-YFP, in living cells), 13 (DDX4, in living cells), 12 (GFP-HP1α, in vitro), 9 (GFP-HP1α + ssDNA, in vitro), 11 (GFP-HP1α, in living cells), 14 (FUS-mCherry, in living cells), 16 (RGG-GFP-RGG, in living cells) or 10 (NPM1, in living cells) independent experiments, +/−SEM.

For the droplet coalescence analysis in Supplementary Fig. 10, 38 (PLL-HA, 0 mM MgCl_2_), 31 (PLL-HA, 25 mM MgCl_2_), 26 (PLL-HA, 50 mM MgCl_2_), 38 (PLL-HA, 100 mM MgCl_2_), 16 (PLL-HA, 150 mM MgCl_2_), 15 (PEG-Rhodamine/dextran), 11 (DDX4, in vitro), 63 (GFP-HP1α, in vitro) or 15 (GFP-HP1α + ssDNA, in vitro) independent fusion events were examined.

The FCS results in Supplementary Fig. 12 are presented as box plots (with the upper and lower bounds of the box corresponding to the first and third quartiles, and the line in the center to the median of the data), using data from 26 (PLL-HA, 0 mM MgCl_2_), 17 (PLL-HA, 25 mM MgCl_2_), 32 (PLL-HA, 50 mM MgCl_2_), 22 (PLL-HA, 100 mM MgCl_2_), 5 (PLL in glycerol) or 10 (PLL, ICBS) independent experiments.

Numbers of independent experiments above refer to experiments with independent condensates or independent cells. No statistical method was used to predetermine sample sizes. No data were excluded from the analyses. The experiments were not randomized. The Investigators were not blinded to allocation during experiments and outcome assessment.

## Supplementary information

Supplementary Information

Editorial Assessment Report

## Data Availability

The source data for all the relevant figures are provided with this paper. Source data are provided with this paper.

## Source data

Source Data

## Code availability

No custom algorithms that are central to the research presented here have been utilized. Scripts for the normalization and interpretation of half-FRAP data are available in the GitHub repository https://github.com/dymochro/MOCHA. An executable Google Colab notebook is also available at https://colab.research.google.com/github/dymochro/MOCHA/blob/main/MOCHA_ColabNotebook.ipynb.

## References

[1] Banani SF, Lee HO, Hyman AA, Rosen MK (2017). Biomolecular condensates: organizers of cellular biochemistry. Nat. Rev. Mol. Cell Biol..

[2] McSwiggen DT, Mir M, Darzacq X, Tjian R (2019). Evaluating phase separation in live cells: diagnosis, caveats, and functional consequences. Genes Dev..

[3] Erdel F, Rippe K (2018). Formation of chromatin subcompartments by phase separation. Biophys. J..

[4] Erdel F (2020). Mouse heterochromatin adopts digital compaction states without showing hallmarks of HP1-driven liquid-liquid phase separation. Mol. Cell.

[5] McSwiggen, D. T. et al. Evidence for DNA-mediated nuclear compartmentalization distinct from phase separation. *Elife* **8**, e47098 (2019). 10.7554/eLife.47098 PMC6522219 31038454

[6] Collombet, S. et al. RNA polymerase II depletion from the inactive X chromosome territory is not mediated by physical compartmentalization. Preprint at *bioRxiv*10.1101/2021.03.26.437188 (2021). 10.1038/s41594-023-01008-5 PMC10442225 37291424

[7] Musacchio A (2022). On the role of phase separation in the biogenesis of membraneless compartments. EMBO J..

[8] Sprague BL (2006). Analysis of binding at a single spatially localized cluster of binding sites by fluorescence recovery after photobleaching. Biophys. J..

[9] Sabari BR, Dall’Agnese A, Young RA (2020). Biomolecular condensates in the nucleus. Trends Biochem. Sci..

[10] Brackley CA, Taylor S, Papantonis A, Cook PR, Marenduzzo D (2013). Nonspecific bridging-induced attraction drives clustering of DNA-binding proteins and genome organization. Proc. Natl. Acad. Sci. USA.

[11] Ancona M, Brackley CA (2022). Simulating the chromatin-mediated phase separation of model proteins with multiple domains. Biophys. J..

[12] Alberti S, Gladfelter A, Mittag T (2019). Considerations and challenges in studying liquid–liquid phase separation and biomolecular condensates. Cell.

[13] Mir, M., Bickmore, W., Furlong, E. E. M. & Narlikar, G. Chromatin topology, condensates, and gene regulation: shifting paradigms or just a phase? *Development* **146**, dev182766 (2019). 10.1242/dev.182766 PMC6803379 31554625

[14] Park S (2020). Dehydration entropy drives liquid–liquid phase separation by molecular crowding. Commun. Chem..

[15] Yewdall, N. A., André, A. A. M., Lu, T. & Spruijt, E. Coacervates as models of membraneless organelles. *Curr. Opin. Colloid Interface Sci*. **52**, 101416 (2021).

[16] Nott TJ (2015). Phase transition of a disordered nuage protein generates environmentally responsive membraneless organelles. Mol. Cell.

[17] Hard R (2018). Deciphering and engineering chromodomain-methyllysine peptide recognition. Sci. Adv.

[18] Nielsen PR (2002). Structure of the HP1 chromodomain bound to histone H3 methylated at lysine 9. Nature.

[19] Kroschwald S, Maharana S, Simon A (2017). Hexanediol: a chemical probe to investigate the material properties of membrane-less compartments. Matters.

[20] Taylor NO, Wei M-T, Stone HA, Brangwynne CP (2019). Quantifying dynamics in phase-separated condensates using fluorescence recovery after photobleaching. Biophys. J..

[21] Brangwynne CP (2009). Germline P granules are liquid droplets that localize by controlled dissolution/condensation. Science.

[22] Novikov DS, Fieremans E, Jensen JH, Helpern JA (2011). Random walk with barriers. Nat. Phys..

[23] Qin J (2014). Interfacial tension of polyelectrolyte complex coacervate phases. ACS Macro Lett..

[24] Cahn JW, Hilliard JE (1958). Free energy of a nonuniform system. I. Interfacial free energy. J. Chem. Phys..

[25] Gouveia B (2022). Capillary forces generated by biomolecular condensates. Nature.

[26] Wang H, Kelley FM, Milovanovic D, Schuster BS, Shi Z (2021). Surface tension and viscosity of protein condensates quantified by micropipette aspiration. Biophys. Rep..

[27] Quail, T. et al. Force generation by protein–DNA co-condensation. *Nat. Phys.* **17**, 1007–1012 (2021).

[28] Muzzopappa F, Hertzog M, Erdel F (2021). DNA length tunes the fluidity of DNA-based condensates. Biophys. J..

[29] Caragine CM, Haley SC, Zidovska A (2018). Surface fluctuations and coalescence of nucleolar droplets in the human cell nucleus. Phys. Rev. Lett..

[30] Renger R (2022). Co-condensation of proteins with single- and double-stranded DNA. Proc. Natl. Acad. Sci. USA.

[31] Jawerth L (2020). Protein condensates as aging Maxwell fluids. Science.

[32] Elbaum-Garfinkle S (2015). The disordered P granule protein LAF-1 drives phase separation into droplets with tunable viscosity and dynamics. Proc. Natl. Acad. Sci. USA.

[33] Shinoda W (2016). Permeability across lipid membranes. Biochim. Biophys. Acta.

[34] Wei C, Pohorille A (2017). Sequence-dependent interfacial adsorption and permeation of dipeptides across phospholipid membranes. J. Phys. Chem. B.

[35] Müller-Ott K (2014). Specificity, propagation, and memory of pericentric heterochromatin. Mol. Syst. Biol..

[36] Schuster BS (2018). Controllable protein phase separation and modular recruitment to form responsive membraneless organelles. Nat. Commun..

[37] Britton S (2014). DNA damage triggers SAF-A and RNA biogenesis factors exclusion from chromatin coupled to R-loops removal. Nucleic Acids Res..

[38] Team, R. C. R: A language and environment for statistical computing. R Foundation for Statistical Computation, Vienna, Austria (2022). https://www.R-project.org.

[39] Pau G, Fuchs F, Sklyar O, Boutros M, Huber W (2010). EBImage-an R package for image processing with applications to cellular phenotypes. Bioinformatics.

[40] Wickham, H. *ggplot2 - Elegant Graphics for Data Analysis* (Springer-Verlag, 2016).

[41] Müller, P. *Python Multiple-tau Algorithm* (Python Package Index, 2012).

[42] Greene, E. C., Wind, S., Fazio, T., Gorman, J. & Visnapuu, M.-L. *Single Molecule Tools: Fluorescence Based Approaches, Part A* Vol. 472, 293–315 (Elsevier, 2010).

[43] Cheng N-S (2008). Formula for the viscosity of a glycerol−water mixture. Ind. Eng. Chem. Res..

[44] Soumpasis DM (1983). Theoretical analysis of fluorescence photobleaching recovery experiments. Biophys. J..

[45] Thompson AP (2022). LAMMPS - a flexible simulation tool for particle-based materials modeling at the atomic, meso, and continuum scales. Comput. Phys. Commun..

[46] Groot RD, Warren PB (1997). Dissipative particle dynamics: Bridging the gap between atomistic and mesoscopic simulation. J. Chem. Phys..

[47] Savitzky A, Golay MJE (1964). Smoothing and differentiation of data by simplified least squares procedures. Anal. Chem..

[48] Carslaw, H. S. & Jaeger, J. C. *Conduction of Heat in Solids* (Oxford University Press, 1959).

[49] Mueller F, Wach P, McNally JG (2008). Evidence for a common mode of transcription factor interaction with chromatin as revealed by improved quantitative fluorescence recovery after photobleaching. Biophys. J..

