# Supplementary material for: Detecting and quantifying liquid–liquid phase separation in living cells by model-free calibrated half-bleaching
Source: Nat Commun. 2022 Dec 16;13:7787. doi: 10.1038/s41467-022-35430-y (PMC9758202; doi:10.1038/s41467-022-35430-y)
Supplement: Supplementary file 1 — Supplementary Information [file 41467_2022_35430_MOESM1_ESM.pdf]

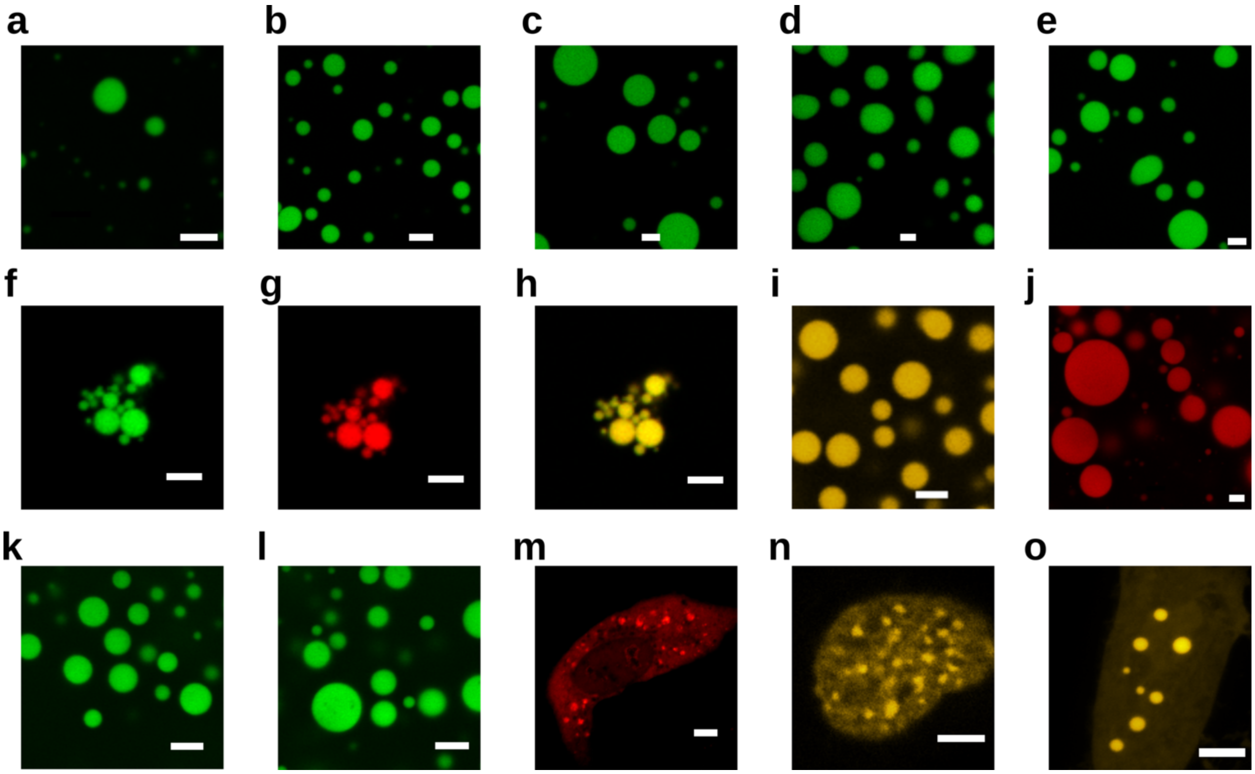

Supplementary Fig. 1. Model systems. Confocal microscopy images of the model systems used in this work. **a-e**, Coacervates composed of PLL-FITC and HA without $MgCl_2$ (a), in the presence of 25 mM $MgCl_2$ (b), 50 mM $MgCl_2$ (c), 100 mM $MgCl_2$ (d), and 150 mM $MgCl_2$ (e). **f-h**, PLL-FITC (f) bound to cross-linked coacervates composed of PLL and HA-Rhodamine (g), with merge image (h)**. i**, DDX4-YFP/PEG condensates. **j**, PEG-Rhodamine/dextran condensates. **k-l**, GFP-HP1α condensates in the presence of 300 mM $CaCl_2$ (k) and additional 50 μM single-stranded DNA (l). **m**, NIH 3T3 cell expressing FUS-mCherry. **n**, NIH 3T3 cell expressing CD*-YFP, a version of the chromodomain of CBX1/HP1β that binds stronger to H3K9me2/3. **o**, NIH 3T3 cell expressing DDX4-YFP. Scale bars, 5 μm.

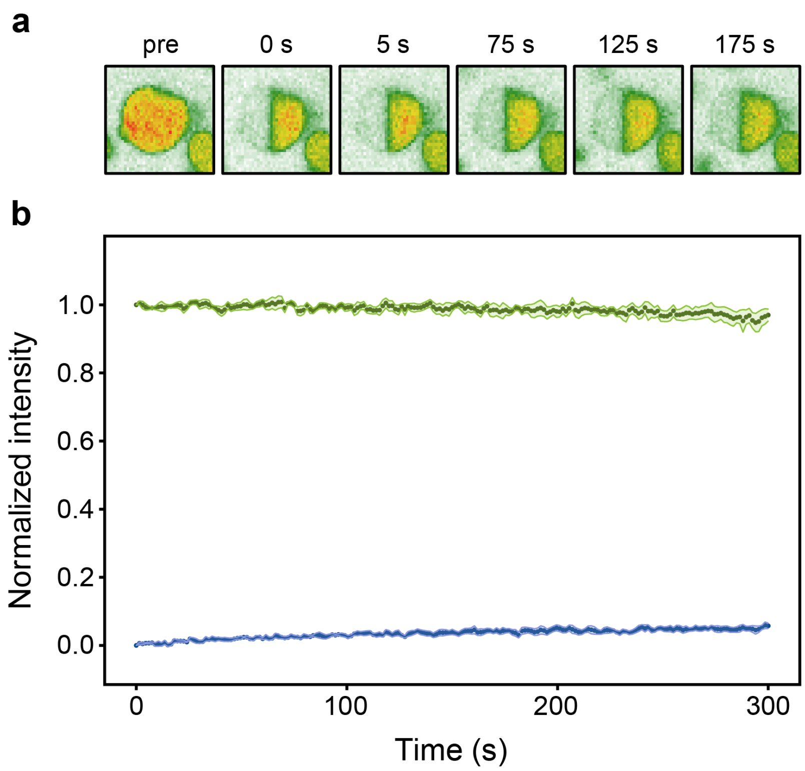

Supplementary Fig. 2. Cross-linked PLL-HA coacervates resemble hydrogels. a, Confocal microscopy images of cross-linked PLL-HA (HA labeled with rhodamine) coacervates during a half-FRAP experiment. Snapshots before the bleach and 0 s, 5 s, 75 s, 125 s and 175 s after the bleach are shown. **b**, Fluorescence recovery in the bleached (blue) and non-bleached (green) halves. The lack of recovery or internal mixing suggests that cross-linked coacervates resemble solid-like hydrogels. Data are shown as mean ± standard error of the mean (s.e.m.) of 5 independent experiments. Source data are provided as a Source Data file.

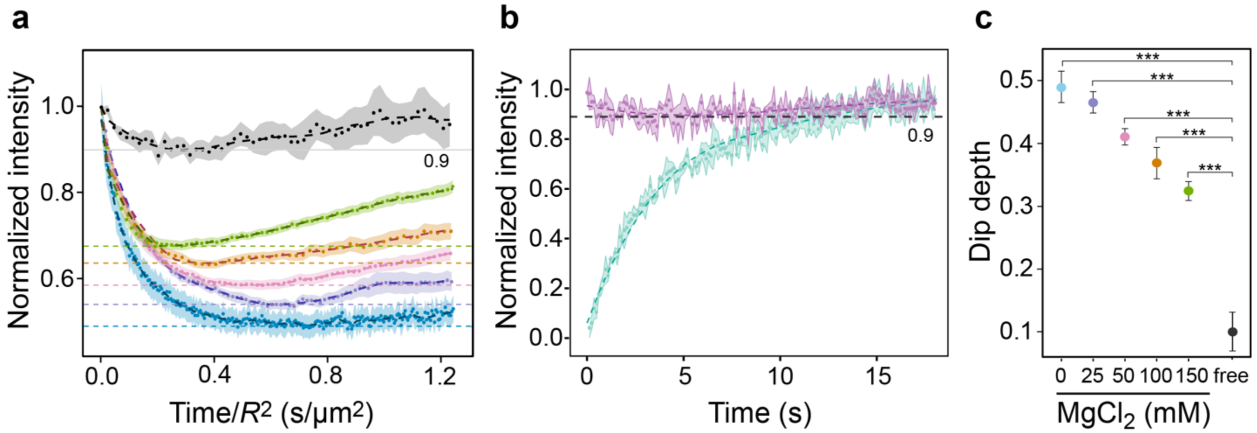

Supplementary Fig. 3. Half-FRAP of PLL-HA condensates at different salt concentrations and of free PLL. a, Recovery curves for the non-bleached halves in half-FRAP experiments performed with PLL-HA without $MgCl_2$ (blue) or in the presence of 25 mM $MgCl_2$ (violet), 50 mM $MgCl_2$ (magenta), 100 mM $MgCl_2$ (orange) and 150 mM $MgCl_2$ (green). The horizontal dashed lines indicate the dip depths for the different conditions. The recovery curve for the non-bleached half of PLL undergoing ICBS at cross-linked coacervates is also depicted (black). The horizontal gray line marks the dip depth for a PLL-FITC solution (free diffusion in the 1-phase regime, see panel b). The following *p*-values were obtained when comparing the dip depths for PLL-HA coacervates to those for PLL-FITC in solution using a one-sided Student's *t*-test: $2 \cdot 10^{-16}$ (0 mM $MgCl_2$), $3 \cdot 10^{-15}$ (25 mM $MgCl_2$), $1 \cdot 10^{-15}$ (50 mM $MgCl_2$), $2 \cdot 10^{-12}$ (100 mM $MgCl_2$), $3 \cdot 10^{-12}$ (150 mM $MgCl_2$), 0.5 (cross-linked coacervates). The time was divided by the squared radius of the droplets to normalize for differences in droplet size. Data are shown as mean ± standard error of the mean (s.e.m.) of at least 8 independent experiments, with dashed lines corresponding to smoothed data (see Methods). **b**, Half-FRAP curves for the bleached half (green) and non-bleached half (violet) of 1 mg/mL PLL-FITC in 50 mM Tris-Cl pH 8 and 25% glycerol (1-phase regime*)*. For the half-FRAP experiment, the solution was pipetted onto a PEG-passivated glass slide, a rectangular ROI of 1.5 µm x 1 µm was bleached, and the intensity was measured in the bleached ROI and in a non-bleached ROI of the same size adjacent to the bleached ROI. The dashed gray line marks the maximum decrease of fluorescence of the non-bleached half in the 1-phase regime. Data are shown as mean ± standard error of the mean (s.e.m.) of 7 independent experiments, with colored dashed lines corresponding to smoothed data (see Methods). The black horizontal dashed line marks the dip depth. **c**, Dip depths for the experiments shown in panels a and b. Dip depths obtained for PLL-HA coacervates at different $MgCl_2$ concentrations are significantly larger than those obtained for free diffusion of PLL (***, $p < 0.001$, one-sided Student's *t*-test). Error bars represent the standard error of the mean (s.e.m.). Source data are provided as a Source Data file.

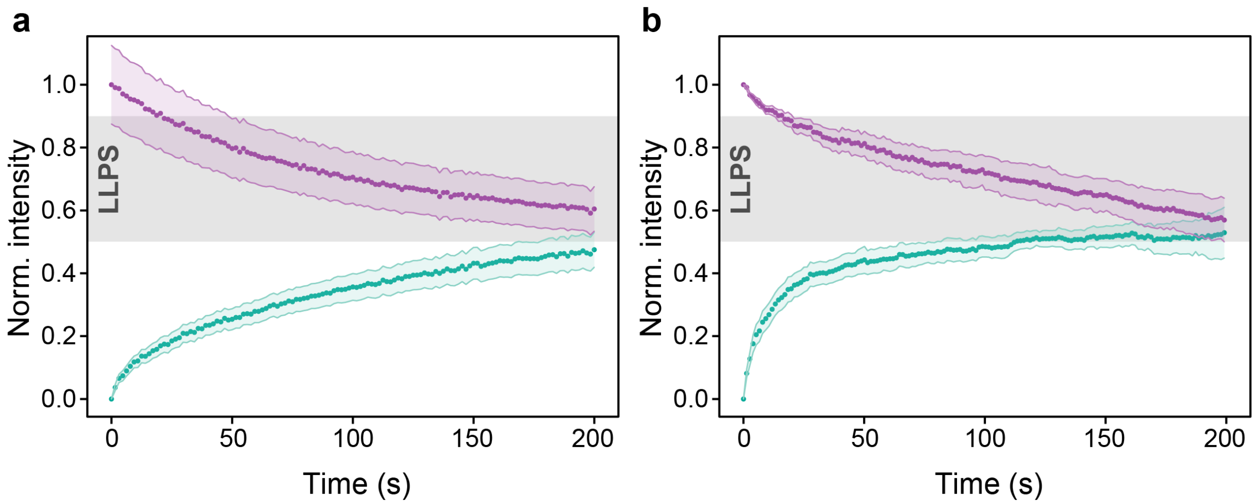

Supplementary Fig. 4. Half-FRAP of PEG/dextran and DDX4-YFP/PEG *in vitro*. a,b, Half-FRAP curves of PEG-Rhodamine/dextran (a) and DDX4-YFP/PEG condensates (b) reconstituted *in vitro*. Violet points represent the normalized recovery in the non-bleached half and green points that in the bleached half. The dip depths fall in the LLPS regime (gray). Data are shown as mean ± standard error of the mean (s.e.m.) of at least 10 independent experiments. Source data are provided as a Source Data file.

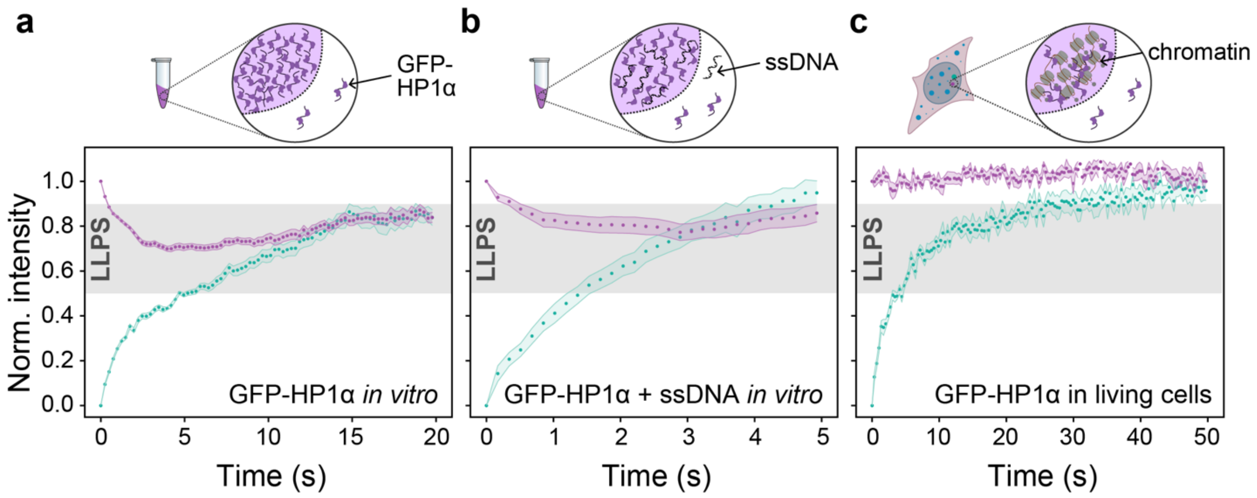

Supplementary Fig. 5. Half-FRAP of GFP-HP1α *in vitro* and in living cells. a,b, Half-FRAP of GFP-HP1α condensates prepared with 50 µM GFP-HP1α in 50 mM Tris-Cl pH 8, 0.33% PEG 20,000 and 300 mM $CaCl_2$ in the absence (a) or presence (b) of additional 50 µM of a 96-mer single-stranded DNA (ssDNA). **c**, Half-FRAP of GFP-HP1α in heterochromatin foci of living NIH 3T3 cells (reanalyzed data from ref. [1]). In all panels, violet data represent the normalized recovery in the non-bleached half and green data the normalized recovery in the bleached half. Data are shown as mean ± standard error of the mean (s.e.m.) of at least 10 independent experiments. Source data are provided as a Source Data file.

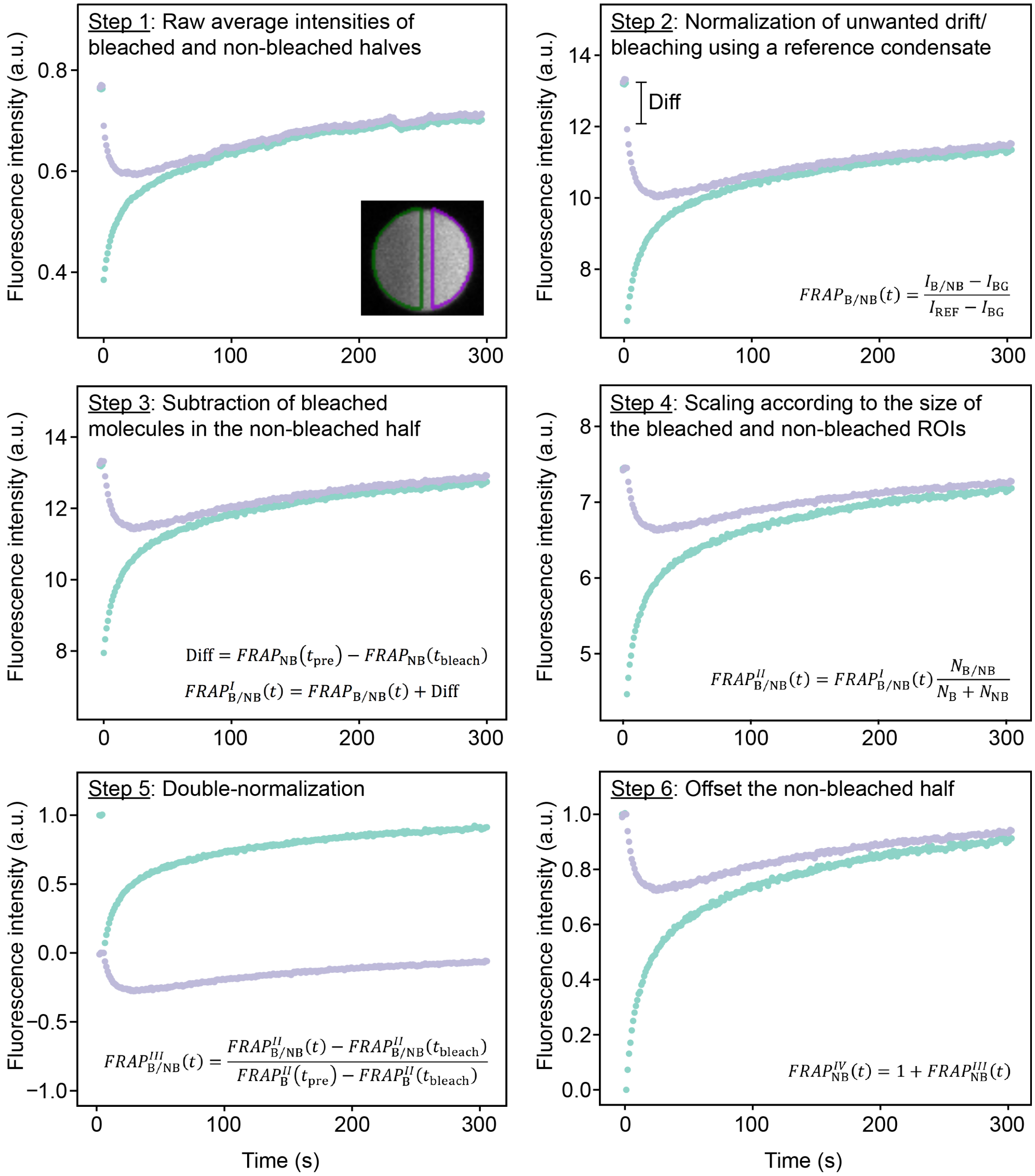

Supplementary Fig. 6. Normalization workflow shown for half-FRAP of a PLL-HA coacervate. Step 1: Image segmentation to quantify the raw fluorescence intensity in the bleached ROI (left ROI, green) and non-bleached ROI (right ROI, violet). The following panels depict the normalization process based on these raw intensities. Step 2: The curves are normalized with an internal reference (another droplet/structure in the field of view) to account for acquisition photobleaching and drift. Step 3: If significant bleaching has taken place in the non-bleached half, it is removed. Step 4: Both halves are multiplied by the ratio between their respective ROI area and the total area of the segmented object (the sum of both ROIs). Step 5: Both halves are double-normalized by subtraction of the intensity of the bleached ROI measured in the first post-bleach frame (proportional to the pool of non-bleached molecules) and division by the intensity difference between the intensity in the bleached ROI in the pre-bleach frames and the first post-bleach frame. Step 6: Finally, the non-bleached half is corrected by addition of an offset so that it equals unity in the pre-bleach frames. These curves can be used to determine the dip depth that is interpreted in MOCHA-FRAP.

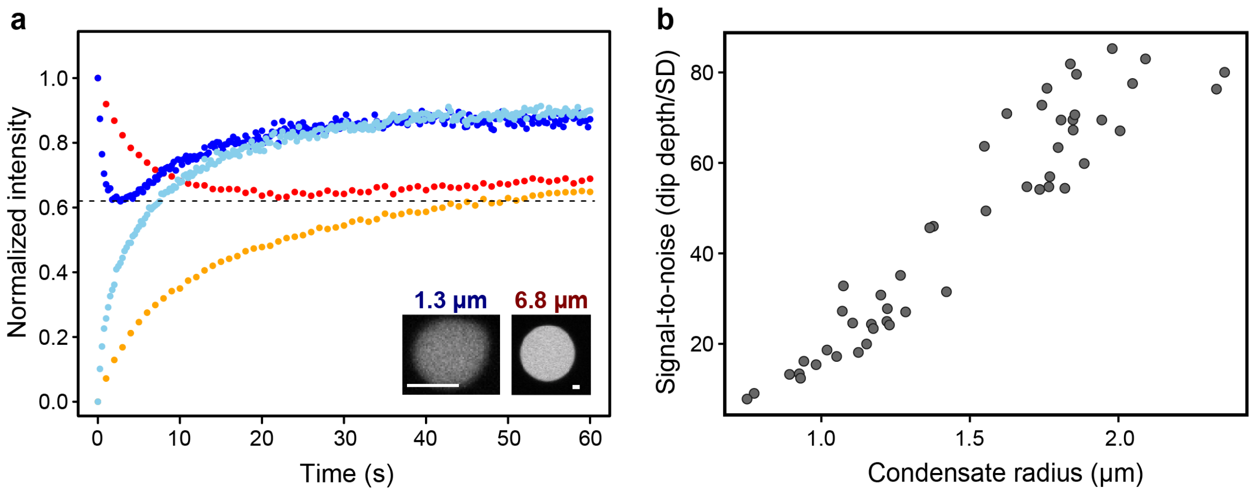

Supplementary Fig. 7. Minimum condensate size required for half-FRAP. a, Representative half-FRAP curves for PLL-HA coacervates with radii of 1.3 µm (blue and skyblue curves) and 6.8 µm (red and orange curves) in the presence of 150 mM $MgCl_2$. The dashed gray line marks the average dip depth for a data set comprising 19 independent experiments. Insets show snapshots of the condensates. Scale bars, 2 µm. **b**, Signal-to-noise ratio calculated as ratio of the measured dip depth over the standard deviation of the curve as a function of the size of the PLL-HA condensates (at 100 mM $MgCl_2$). To obtain the standard deviation, data were smoothed using a Savitzky-Golay filter, the smoothed data were subtracted from the measured data, and the standard deviation of this difference was calculated. Source data are provided as a Source Data file.

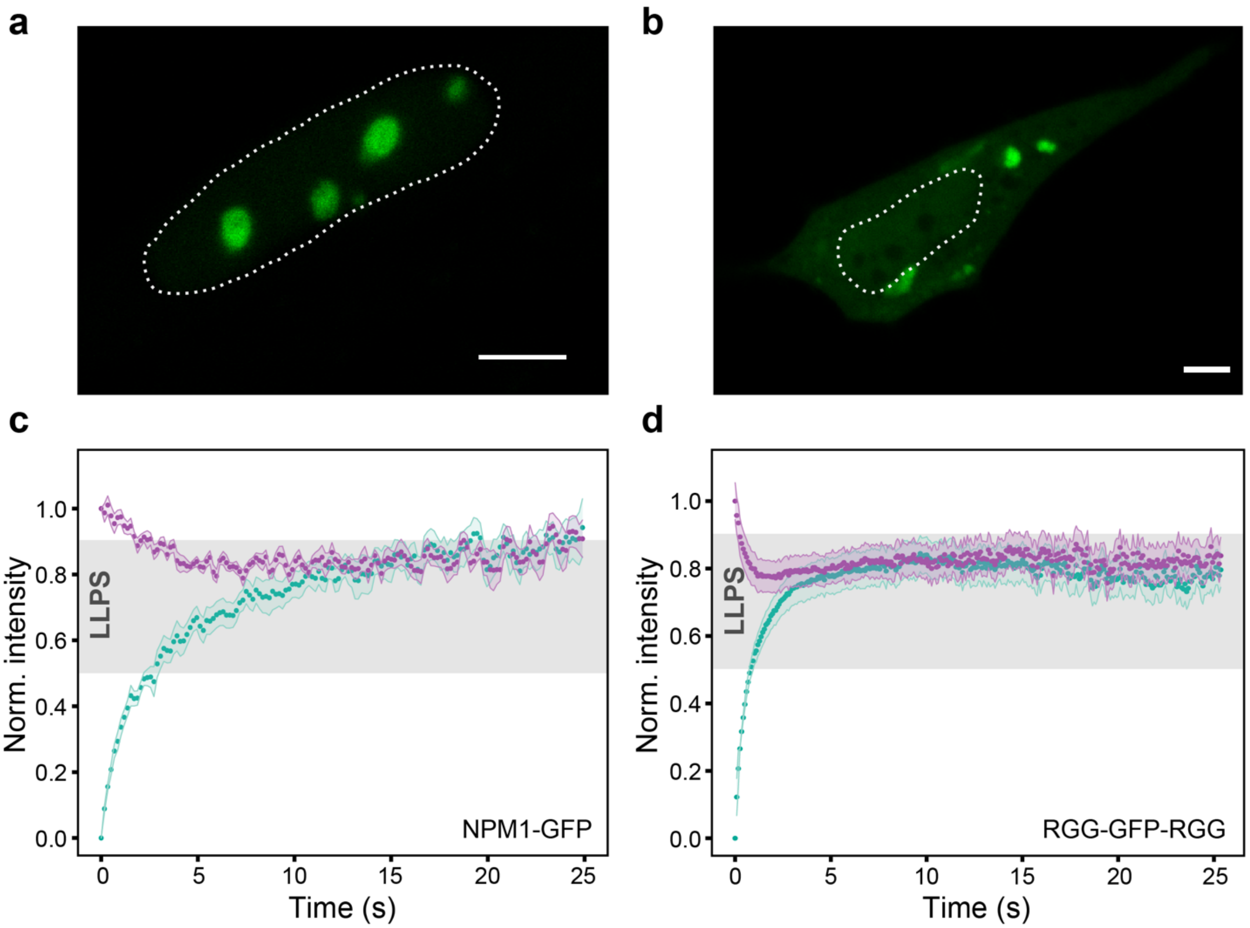

Supplementary Fig. 8. Half-FRAP of NPM1-GFP and RGG-GFP-RGG in living cells. a-b, Confocal microscopy images of NIH 3T3 cells expressing NPM1-GFP (a) and RGG-GFP-RGG derived from LAF-1 (b). Scale bar, 5 µm. **c-d,** Half-FRAP curves of NPM1-GFP in nucleoli and RGG-GFP-RGG in cytoplasmic condensates in living cells. Violet data represent the normalized recovery in the non-bleached half and green data the normalized recovery in the bleached half. The dip depths fall in the LLPS regime (gray). Data are shown as mean ± standard error of the mean (s.e.m.) of at least 10 independent experiments. Source data are provided as a Source Data file.

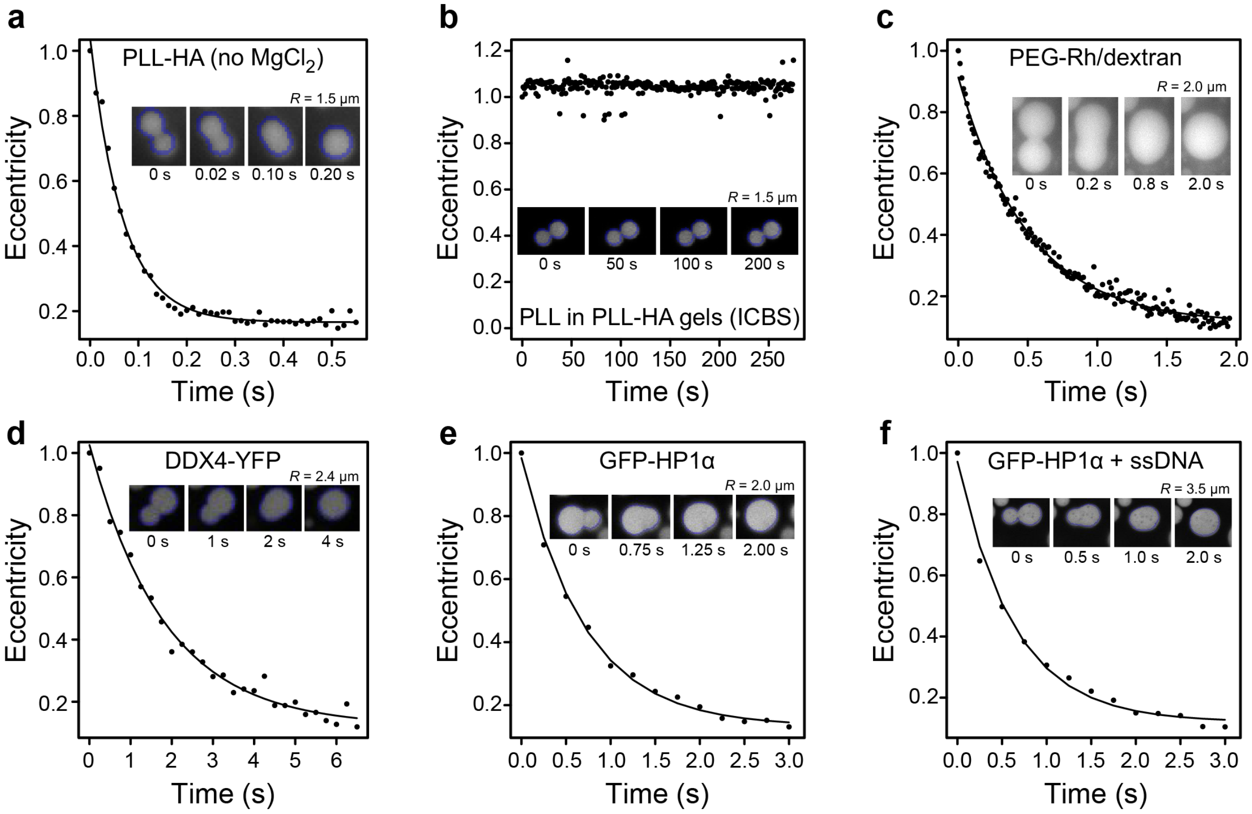

Supplementary Fig. 9. Droplet coalescence measurements. Snapshots of different types of condensates undergoing coalescence *in vitro*. During coalescence, the eccentricity, which reflects the condensate shape, was quantified and fitted to a decaying exponential function. Representative droplet coalescence events of PLL-HA coacervates (a), crosslinked PLL-HA coacervates (b), PEG-Rhodamine/dextran (c), DDX4-YFP/PEG (d), GFP-HP1α/PEG (e) and GFP-HP1α/PEG + single-stranded DNA (f) are depicted. The radius of the corresponding droplet is shown above each series of snapshots. No coalescence was observed for cross-linked PLL-HA coacervates. Source data are provided as a Source Data file.

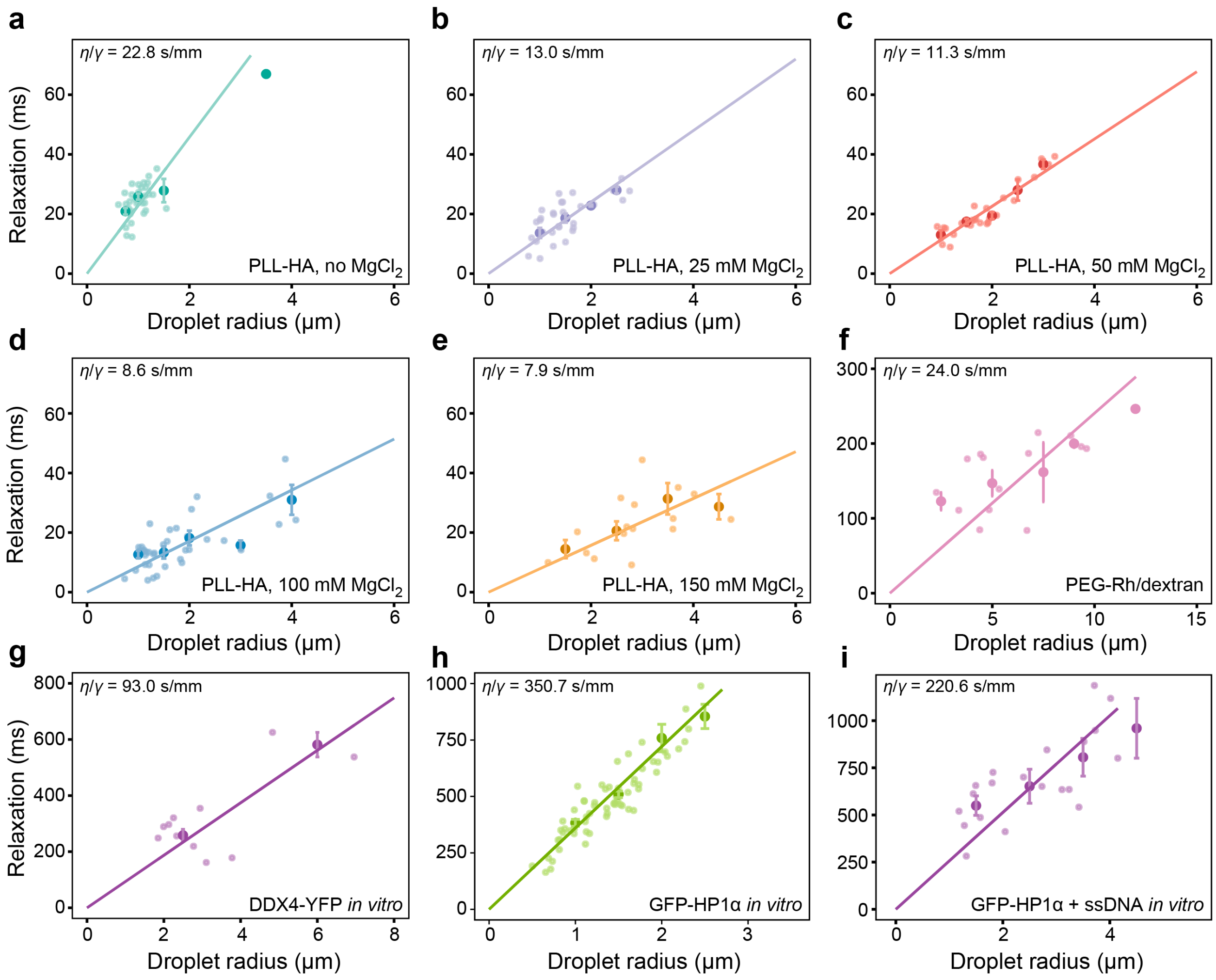

Supplementary Fig. 10. Inverse capillary velocity measurement. Relaxation times obtained from the exponential fits of the droplet eccentricity during coalescence were plotted versus the size of the droplets to obtain the inverse capillary velocity for PLL-HA coacervates in the absence (a) or presence of 25 mM (b), 50 mM (c), 100 mM (d) and 150 mM (e) $MgCl_2$, for PEG-Rhodamine/dextran (f), DDX4-YFP/PEG (g), GFP-HP1α/PEG (h) and GFP-HP1α/PEG condensates in the presence of single-stranded DNA (i). The inverse capillary velocities obtained from fitting the data to linear functions are depicted in each plot. Error bars represent standard deviations. Source data are provided as a Source Data file.

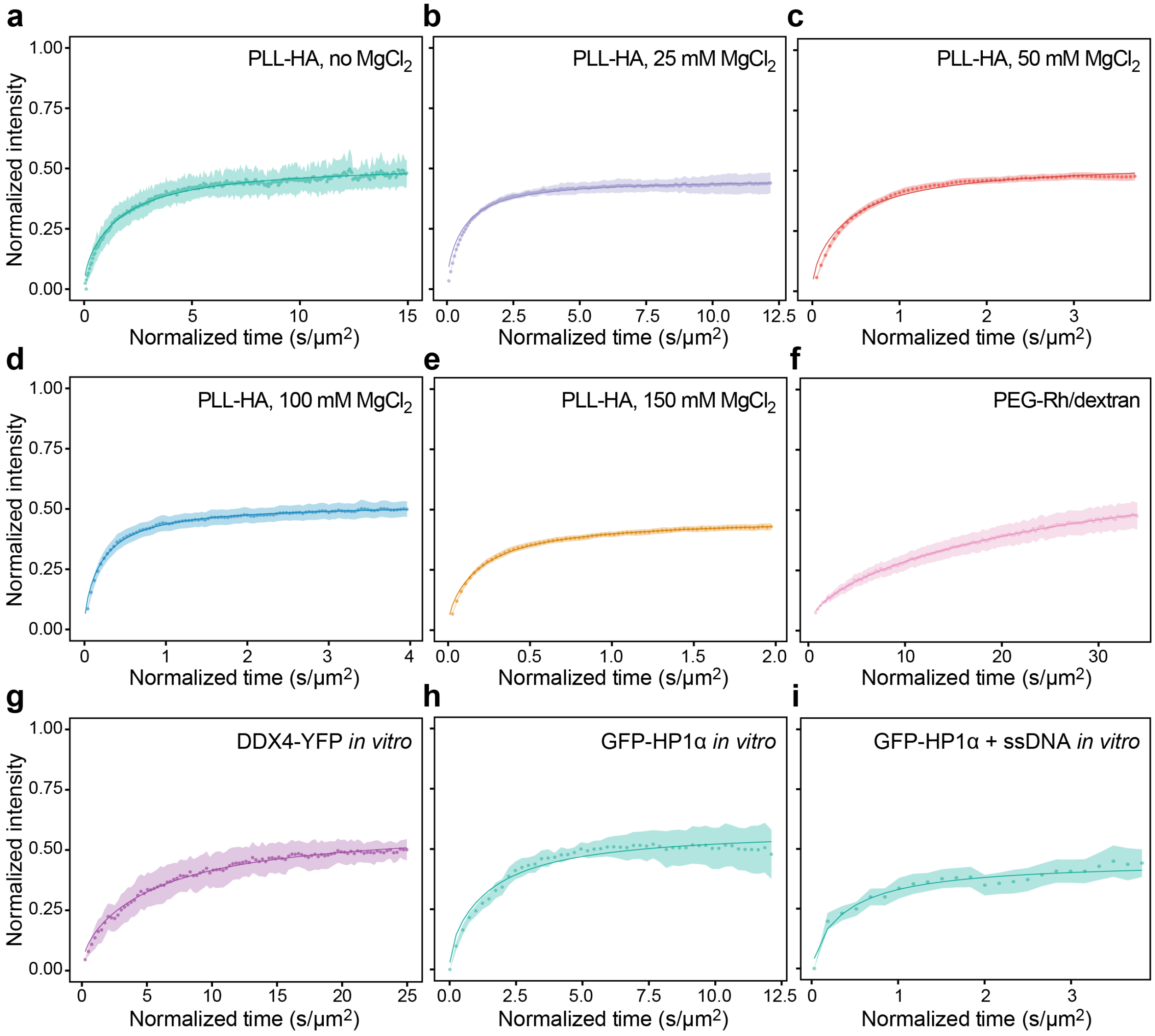

Supplementary Fig. 11. Estimation of diffusion times from half-FRAP. Recovery curves for internal mixing, calculated as F_{half} - F_{full}, from half-FRAP experiments of PLL-HA in the absence of $MgCl_2$ (a) and in the presence of 25 mM (b), 50 mM (c), 100 mM (d) and 150 mM (e) $MgCl_2$. Same for DDX4-YFP/PEG (f), PEG-Rhodamine/dextran (g), GFP-HP1α/PEG (h) and GFP-HP1α/PEG in the presence of single-stranded DNA (i). Data are shown as mean ± standard error of the mean (s.e.m.) of at least 8 independent experiments. Solid lines correspond to fits using a pure diffusion model (see Methods). Normalized times correspond to times divided by the squared radius of the droplets. Source data are provided as a Source Data file.

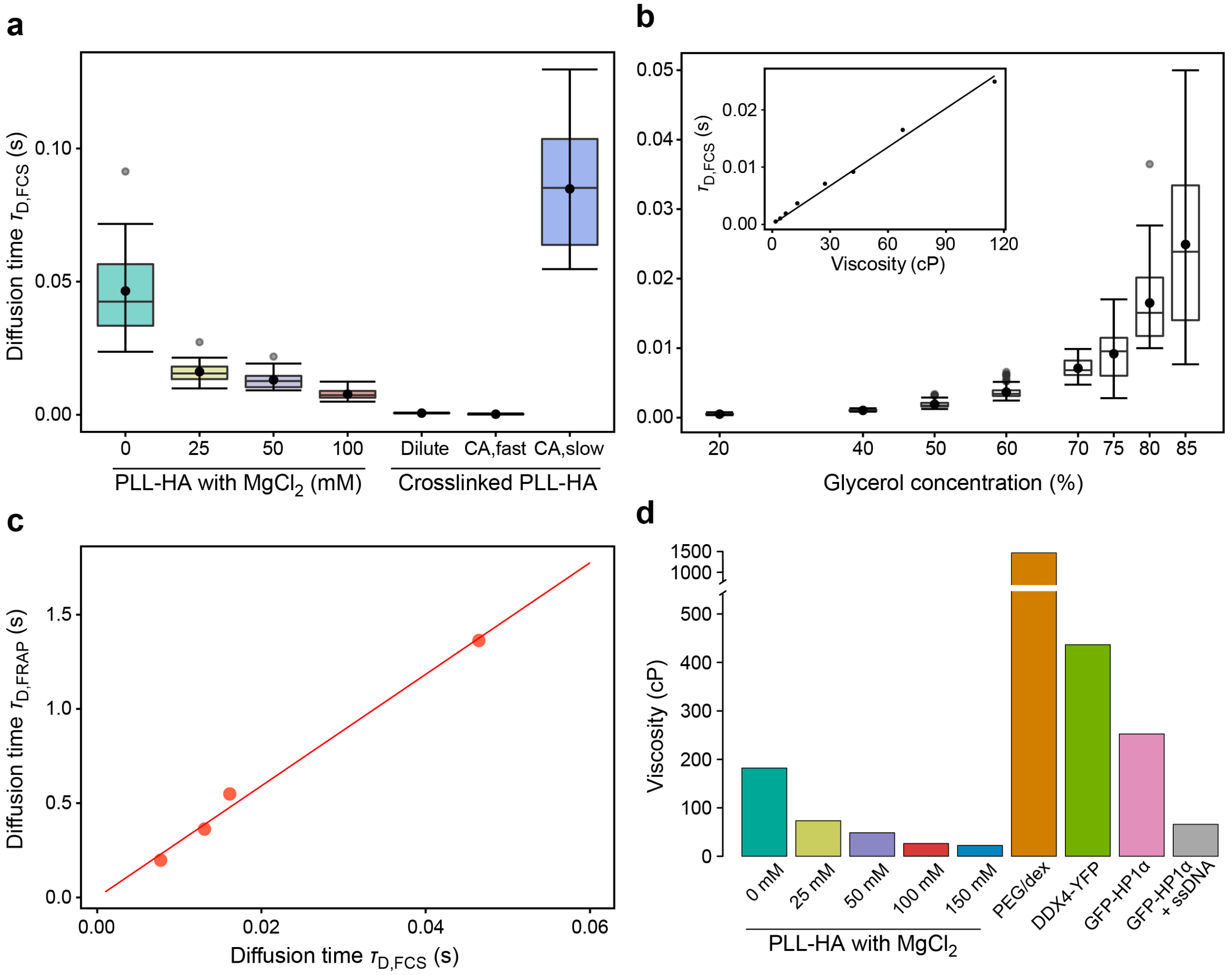

Supplementary Fig. 12. Viscosity measurement by Fluorescence Correlation Spectroscopy. a, Translational diffusion times determined by FCS for PLL-ATTO647N in PLL-HA coacervates at different $MgCl_2$ concentrations. For cross-linked coacervates, the diffusion time obtained in the dilute phase and the two diffusion times for the two components in the coacervate are shown. The slow/fast components likely correspond to PLL-ATTO647N molecules diffusing across the observation volume with/without interacting with cross-linked coacervates, respectively. **b**, Translational diffusion times determined by FCS for PLL-ATTO647N in different glycerol-water mixtures with known viscosities, which were obtained as previously described [1]. The inset shows the linear relationship between diffusion time and viscosity. Error bars represent standard deviations. **c**, Relationship between diffusion times obtained by FCS and half-FRAP for PLL-HA coacervates at different $MgCl_2$ concentrations. Based on this calibration curve, diffusion times from half-FRAP (Supplementary Fig. 11) were converted to viscosities. **d**, Viscosities obtained from the diffusion times measured in different condensates. Source data are provided as a Source Data file.

KEY REAGENTS			
**Name of product**	**Source**	**Reference code**	**Lot number**
Poly-L-Lysine hydrobromide	Sigma-Aldrich	P7890-100MG	SLCC9284
Poly-L-Lysine-FITC labeled	Sigma-Aldrich	P3543-10MG	SLBQ1702V
EDC (1-Ethyl-3-(3-dimethylaminopropyl) carbodiimide	Thermo Fisher Scientific	22980	VB291479
Fluorescein isothiocyanate -— DEXTRAN	Sigma-Aldrich	46946-100MG	BCCB3932
N-Hydroxysulfosuccinimide sodium	Sigma-Aldrich	56485-1G	BCCC9854
Hyaluronic acid sodium salt (HA)	Sigma-Aldrich	40583-10MG	BCCC8097
Hyaluronate Rhodamine (HA-Rh)	HAWORKS	HA-Rhodamine-10K	TM3-002
NH2-PEG-NH2 (8000)	Nanocs	PG2-AM-8k	140224
NHS-Rhodamine	Thermo Fisher Scientific	46406	VC2953691
Atto 647N - NHS	Atto	AD647N-31	08AA03
PEG 8 KDa	Sigma-Aldrich	P-5413	128400015
PEG 20 kDa	Fluka	813000	2034733
$MgCl_2$	Sigma-Aldrich	M2670	BCCB1114
1,6-Hexanediol	Sigma-Aldrich	240117-50G	MKCL0369
Lipofectamine 2000	invitrogen	52887	2141457
1,2-Dioleoyl-sn-glycero-3-phosphocholine (DOPC)	Avanti - Lipids	850375C-1G-B-332	4235-95-4
1,2-dioleoyl-sn-glycero-3-phosphoethanolamine-N-[methoxy(polyethylene glycol)-2000] (DOPE-PEG)	Avanti - Lipids	880130P-25MG-049	474922-90-2

OLIGONUCLEOTIDES	
**Name**	**Sequence**
DDX4-YFP_Fw	gcttggtaccgagctcggatccaacATGgGCG
DDX4-YFP_Rv	TGAAgaattcTTAAGTGATCCCGGCGGCGGTCAC
ssDNA for GFP-HP1α phase separation assays	TATGCGGCCGCTTACTTGTCATCGTCATCCTTGTAATCGATG TCATGATCTTTA TAATCACCGTCATGGTCTTTGTAATCTGGC TTGAGATAATGAAAG

Supplementary Table 1. Key reagents. List of key reagents used in this work, including source, reference code and lot number. The oligonucleotides used to clone DDX4-YFP into the pGEX-6P-1 expression plasmid and the oligonucleotide used for phase separation assays are also listed.

FIT TABLE					
**MODEL**	$F_{\mathrm{diff}}(t) = Ae^{-\frac{2\tau_D}{t}}[I_0(2\tau_D/t) + I_1(2\tau_D/t)]$				
**SAMPLE**	PLL-HA	PLL-HA 25 mM $MgCl_2$	PLL-HA 50 mM $MgCl_2$	PLL-HA 100 mM $MgCl_2$	PLL-HA 150 mM $MgCl_2$
**EXPERIMENT**	F_{half} - F_{full}	F_{half} - F_{full}	F_{half} - F_{full}	F_{half} - F_{full}	F_{half} - F_{full}
$\boldsymbol{T_D}$	1.36 ± 0.02	0.55 ± 0.01	0.36 ± 0.01	0.20 ± 0.01	0.17 ± 0.01
$\boldsymbol{A}$	0.52 ± 0.01	0.46 ± 0.01	0.54 ± 0.01	0.52 ± 0.01	0.47 ± 0.01
**Resid. fit error**	0.002355	0.001889	0.003437	0.002017	0.000979
**SAMPLE**	PEG/dextran	DDX4-YFP/ PEG	GFP-HP1α/ PEG	GFP-HP1α/ PEG + ssDNA	PLL-HA
**EXPERIMENT**	F_{half} - F_{full}	F_{half} - F_{full}	F_{half} - F_{full}	F_{half} - F_{full}	Partial-FRAP
$\boldsymbol{T_D}$	17.7 ± 3.1	1.7 ± 0.1	1.9 ± 0.1	0.35 ± 0.04	2.9 ± 0.5
$\boldsymbol{A}$	0.55 ± 0.06	0.53 ± 0.01	0.58 ± 0.01	0.48 ± 0.01	0.52 ± 0.01
**Resid. fit error**	0.002856	0.002904	0.02358	0.03464	0.002355
**SAMPLE**	PLL-HA 25 mM $MgCl_2$	PLL-HA 50 mM $MgCl_2$	PLL-HA 100 mM $MgCl_2$	PLL-HA 150 mM $MgCl_2$	PLL undergoing ICBS
**EXPERIMENT**	Partial-FRAP	Partial-FRAP	Partial-FRAP	Partial-FRAP	Partial-FRAP
$\boldsymbol{T_D}$	2.5 ± 0.2	1.7 ± 0.1	1.1 ± 0.2	1.2 ± 0.3	2.2 ± 0.3
$\boldsymbol{A}$	0.75 ± 0.01	0.87 ± 0.01	0.95 ± 0.01	1.00 ± 0.01	1.00 ± 0.01
**Resid. fit error**	0.003481	0.004367	0.006185	0.002381	0.009392
**SAMPLE**	CD*-YFP live-cell	DDX4-YFP live-cell	PLL-HA 25 mM $MgCl_2$	PLL-HA 50 mM $MgCl_2$	PLL-HA 100 mM $MgCl_2$
**EXPERIMENT**	Partial-FRAP	Partial-FRAP	Full-FRAP	Full-FRAP	Full-FRAP
$\boldsymbol{T_D}$	0.4 ± 0.1	7.0 ± 0.2	20 ± 8	9 ± 1	10 ± 1
$\boldsymbol{A}$	0.54 ± 0.01	0.53 ± 0.01	0.19 ± 0.03	0.36 ± 0.01	0.82 ± 0.01
**Resid. fit error**	0.01785	0.001628	0.002758	0.001332	0.002721
**SAMPLE**	PLL-HA 150 mM $MgCl_2$	PLL undergoing ICBS	CD*-YFP live-cell	DDX4-YFP live-cell	
**EXPERIMENT**	Full-FRAP	Full-FRAP	Full-FRAP	Full-FRAP	
$\boldsymbol{T_D}$	5 ± 1	47 ± 12	1.7 ± 0.1	6.6 ± 0.3	
$\boldsymbol{A}$	0.90 ± 0.01	2.00 ± 0.25	0.76 ± 0.01	0.71 ± 0.01	
**Resid. fit error**	0.00373	0.002482	0.008249	0.006284	

FIT TABLE					
**SAMPLE**	PLL-HA	PLL-HA 25 mM $MgCl_2$	PLL-HA 50 mM $MgCl_2$	PLL-HA 100 mM $MgCl_2$	PLL-HA 150 mM $MgCl_2$
**EXPERIMENT**	*Viscosity measurement*				
Viscosity η (cP)	182 ± 8	73 ± 4	49 ± 6	26 ± 7	22 ± 2
**SAMPLE**	PEG/dextran	DDX4/PEG	HP1α/PEG	HP1α/PEG + ssDNA	
**EXPERIMENT**	*Viscosity measurement*				
Viscosity η (cP)	1612 ± 53	436 ± 12	270 ± 4	65 ± 4	
**SAMPLE**	PLL-HA	PLL-HA 25 mM $MgCl_2$	PLL-HA 50 mM $MgCl_2$	PLL-HA 100 mM $MgCl_2$	PLL-HA 150 mM $MgCl_2$
**EXPERIMENT**	*Inverse capillary velocity measurement*				
η/γ (s/mm)	22.8 ± 0.8	13.0 ± 0.6	11.3 ± 0.3	8.6 ± 0.5	7.9 ± 0.7
**SAMPLE**	PEG/dextran	DDX4/PEG	HP1α/PEG	HP1α/PEG + ssDNA	
**EXPERIMENT**	*Inverse capillary velocity measurement*				
η/γ (s/mm)	24.0 ± 1.0	93.0 ± 1.0	350.7 ± 5.0	220.6 ± 1.3	

**MODEL**	$Dip = \frac{0.5 - b}{1 + \left(\frac{\Delta E}{a}\right)^n} + 0.5$	**MODEL**	$T_{D,FCS} = \eta * A$	$T_{D,FRAP} = T_{D,FCS} * A$
**SAMPLE**	*in vitro* LLPS systems	**SAMPLE**	PLL-ATTO647N in water/glycerol	PLL-HA
**EXPERIMENT**	Energy ΔE vs dip depth	**EXPERIMENT**	FCS calibration	FCS & FRAP
a	(0.026 ± 0.003) kT	***A***	(0.003 ± 0.001) s/cP	29.6 ± 0.9
n	2.00 ± 0.38	**Resid. fit error**	9.6E-06	0.04753
b	0.12 ± 0.03			
**Resid. fit error**	0.154			

Supplementary Table 2. Table of fit parameters. In the first part of the table, the parameters used to fit the partial-FRAP, full-FRAP and internal mixing (F_{half} - F_{full} from half-FRAP) curves with a pure diffusion model (see Methods) are shown. Inverse capillary velocities obtained by fitting the droplet coalescence relaxation for different droplet sizes (Supplementary Figs. 9 and 10) and viscosities obtained from fitting FCS/FRAP curves (Supplementary Fig. 11) are also reported. Errors correspond to standard fit errors. The second part of the table contains the fit parameters to fit the dip depth of half-FRAP *in vitro* experiments vs. the interfacial energy per molecule to a Hill equation. It also contains the fit parameters of the linear equation used to fit the diffusion times obtained from FCS of PLL in glycerol-water mixtures to the viscosity and to fit the diffusion times obtained from FCS to those obtained from half-FRAP measurements.

**Hydrodynamic radii**			
molecule	*hydrodynamic radius (nm)*	*technique*	*reference*
DDX4-YFP	5.15	Alphafold prediction	(2)
Poly-lysine, MW = 24 kDa	2.7	Stokes-Einstein equation	(3)
PEG, MW = 8 kDa	2.4	SANS	(4)
NPM1-GFP	3.75	Alphafold prediction	(2)
RGG-GFP-RGG	5.81	Alphafold prediction	(2)
FUS-mCherry	6.20	Alphafold prediction	(2)
GFP-HP1α	5.0	Alphafold prediction	(2)

Supplementary Table 3. Literature values of hydrodynamic radii. Hydrodynamic radii that were used to calculate the interfacial energy per molecule.

Protein/ Organelle	Experiment	Interfacial tension (μN/m), interfacial energy (μJ/m^2)	Reference	Comment
**LAF-1**	*in vitro*	680	(5)	-
**LAF-1**	*in vitro*	250	(6)	in absence of RNA/DNA
**LAF-1**	*in vitro*	150	(7)	in absence of RNA/DNA
RGG-GFP-RGG	live-cell	0.5	this work	
**FUS**	*in vitro*	3	(8)	in absence of RNA/DNA
**FUS**	*in vitro*	0.015	(9)	in presence of ssDNA
**FUS**	*in silico*	10-400	(10)	-
FUS-mCherry	live-cell	0.3	this work	
**NPM1**	*in vitro*	~ 0.8	(11)	*Xenopus* NPM1
**Nucleoli**	live-cell	~ 0.4	(11)	*Xenopus* oocytes
**Nucleoli**	live-cell	~ 1	(12)	HeLa cells
NPM1-GFP	live-cell	0.5	this work	
DDX4-YFP	live-cell	1.7	this work	
DDX4-YFP/PEG	*in vitro*	4.7	this work	
GFP-HP1α/PEG	*in vitro*	0.8	this work	
GFP-HP1α/PEG	*in vitro*	0.3	this work	in presence of ssDNA (1:1)

Supplementary Table 4. Values of interfacial tensions and energies. Values refer to reconstituted condensates *in vitro*, entire organelles in living cells, or *in silico* simulations (as indicated).

Supplementary Note 1. Kinetic models for LLPS and ICBS.

This Supplementary Note is intended to provide a motivation and an extended explanation of the kinetic models we used to describe molecules undergoing liquid-liquid phase separation (LLPS) or low-valency interactions with spatially clustered binding sites (ICBS) on a long polymeric scaffold. The key difference between both scenarios is that molecules undergoing LLPS establish multivalent interactions with one another, which are critical for LLPS [13–16]. Monovalent molecules, which can only interact with one other molecule, would rather form a solution of dimers than a condensate, which is enthalpically equivalent but entropically more favorable. Divalent molecules would form a solution of filaments [15]. As molecules in a condensate formed by LLPS establish multiple interactions simultaneously, the probability that they are "free", in the sense that they do not undergo any interaction at a given time point, is very low. Rather, they form a percolated network of interactions, which has been shown to be important for LLPS to occur [15]. Consistently, the molecules in a condensate formed by LLPS can be considered to reside in an effective potential well, whose "wall" coincides with the interface of the condensate [17]. To leave the condensate, molecules have to break the interactions with their partners and overcome this "wall" at the interface. We have therefore described the dynamics of these molecules with a random walk across a barrier [18]. The detailed calculations, which follow previous work [18,19], are presented in the Methods section (see *Half-FRAP model for diffusion in a circle with a semi-permeable boundary*).

In the ICBS scenario, molecules do not establish multivalent interactions with one another but rather low-valency interactions with a long polymer segment, such as a piece of a chromosome. For simplicity, the polymer segment can be considered to be immobile, which is justified if the bulk of the polymer stays within the structure of interest during the course of the experiment. In contrast to the LLPS scenario above, molecules alternate between a "bound" state, during which they interact with a binding site on the immobile polymer segment, and a "free" state, during which they freely diffuse. The average durations that molecules spend in each of these states are given by the inverse binding rates [20]. Accordingly, there is a considerable probability that a molecule is "free", which is different from the LLPS scenario, in which molecules are multivalent and can therefore be expected to still undergo interactions after a particular interaction has been broken. The "free" molecules in the ICBS scenario, which are the mobile molecules within the structure of interest, do not reside in a potential well and do not have to cross a "wall" at the interface when leaving the structure. The detailed calculations, which follow previous work [20–22], are presented in the Methods section (see *Half-FRAP model for a reaction-diffusion process in a circle with a fully permeable boundary*). They explicitly contain expressions describing the dynamics of the pools of molecules that are "free" or "bound" at a given time point.

The models we used here to describe LLPS and ICBS are minimal models with similar complexity, allowing for a straightforward comparison between both scenarios. They capture the fundamental difference between both cases, namely the presence or absence of an interfacial barrier, which can be traced back to the prevalence of multivalent interactions (see for example our simulations in **Fig. 3g-i**). This feature is expected to be also present in more complex models, e.g., if both weak interactions with immobile binding sites and multivalent self-interactions are considered (similar to the scenario simulated in **Fig. 3g-i**).

Supplementary Note 2. MOCHA-FRAP tutorial.

MOCHA-FRAP is a quantitative half-FRAP approach to assess if molecules in a structure of interest undergo liquid-liquid phase separation (LLPS) driven by multivalent interactions, or if they undergo low-valency interactions with spatially clustered binding sites (ICBS) on an immobile scaffold. MOCHA-FRAP also quantifies the apparent energy barrier at the interface of structures of interest. This tutorial is intended to give an overview of the individual steps of the workflow. A Google Colab and a Jupyter notebook (see Code Availability Statement) have been made available for the analysis of MOCHA-FRAP experiments.

1. **Sample considerations**

   MOCHA-FRAP can be used to study both *in vitro* systems and living cells. When studying labeled proteins in living cells, it should be beard in mind that LLPS is a concentration-dependent process that can be triggered by excessive overexpression, which should be avoided. For *in vitro* systems, ideally only a small fraction of proteins is labeled to reduce potential artifacts induced by the dye. Furthermore, the buffer conditions and the protein concentration should be optimized to mimic those inside the cell. The glass surface on which the sample is placed should be passivated before the experiment to mitigate potential artifacts caused by interactions of the studied molecules with the glass surface.

2. **Half-FRAP experiment**

   There are several microscopy parameters that should be optimized for a half-FRAP experiment. The size of the frame as well as the scanning speed should be chosen to reach the desired time resolution. From a first batch of experiments conducted with high time resolution, the half time of recovery should be estimated to then choose a time resolution that allows proper sampling of the recovery process. This is important because the dip in the non-bleached half can be present at very early time points. However, oversampling could lead to unnecessary acquisition photobleaching and should be avoided. Similarly, the laser intensity for image acquisition is chosen to obtain a good compromise between a high signal-to-noise ratio and low acquisition photobleaching. The parameters for the photobleaching step should be chosen so that at least half of the intensity in one half is bleached while photobleaching in the other half should be minimal.

3. **Analysis of individual half-FRAP curves**

   After data acquisition, each half-FRAP experiment is analyzed individually. In brief, the intensities of the bleached and non-bleached halves are quantified along with the total intensity in the cell/nucleus, which serves as a reference for acquisition photobleaching. In case the condensate has moved during the experiment, a registration is performed. These steps can be done with an image analysis software, such as Fiji, or with the python script that we have integrated into a Google Colab notebook (see Code Availability Statement). The intensities in the bleached and non-bleached half are then normalized as described in the Methods section (*Half-FRAP data analysis*). This normalization includes the removal of a potential immobile fraction, which is important to accurately quantify the dip depth.

4. **Analysis of averaged half-FRAP curves**

Once a sufficiently large number of individual half-FRAP experiments have been analyzed as described in the step above, the average and standard deviation of the normalized intensities in the bleached and non-bleached half are calculated, using any data analysis software (e.g., Excel, RStudio). To average curves originating from different-sized structures, for which dips are present at different time points, the time axes should be normalized before averaging. To do so, normalized half-FRAP curves are rescaled along the time axis using the normalized time $t' = t/R^2$, where t is the time and R is the radius of the half-bleached structure. This rescaling step does not affect the dip depth. Next, the average curve is smoothed with a Savitzky-Golay filter and its minimum is numerically determined, yielding the dip depth. The error of the dip depth corresponds to the standard deviation of the curve at the minimum. According to our experiments, freely diffusing proteins yield a dip depth of 10 ± 3 %, while LLPS yields larger dip depths and ICBS yields equal or smaller dip depths (see also our calculations). To test if the measured dip depth is significantly larger than that obtained for the case of free diffusion, which would mean that molecules undergo LLPS, a one-sided Student's t-test can be performed. Our reference data set for free diffusion, which can be used in this test, has a mean dip depth $\mu = 0.1$ (10 %), a standard deviation $\sigma = 0.0283$ (2.83 %), and a sample size $N = 7$. Alternatively, a reference data set can be generated by bleaching the protein of interest in solution or in a living cell outside of a condensate, if applicable. In general, the sample size for both data sets should be chosen according to the expected standard deviation (in our experiments, typical standard deviations of $\sigma \approx 0.03$ were obtained) and the desired confidence level for the distinction between LLPS and ICBS (e.g., $\alpha = 0.01$).

5. **Determination of the energy barrier**

If the dip depth obtained in the step above signals the presence of an interfacial barrier, i.e., if it is significantly larger than that for free diffusion, this dip depth can be used along with the calibration curve determined in this manuscript to estimate the energy barrier at the interface of the condensate. This energy corresponds to the energetic cost for leaving the condensate, which can also be used as a proxy for the cohesive intermolecular interactions in the condensate. A more detailed explanation of the energy barrier and its interpretation is provided in the manuscript.

6. **Troubleshooting**

**Problem**	**Potential reason**	**Solution**
Strong bleaching in the nominal non-bleached half	Excessive bleaching	Decrease laser power or shorten the pixel dwell time during bleaching
Condensate gets smaller or bigger during the experiment	Condensate moves along the *z*-axis	Open the pinhole to minimize intensity changes due to motion along the *z*-axis
Total intensity decreases during the experiment	Acquisition photobleaching	Decrease the laser intensity used to acquire the post-bleach images

Supplementary References

1. Erdel, F. *et al.* Mouse Heterochromatin Adopts Digital Compaction States without Showing Hallmarks of HP1-Driven Liquid-Liquid Phase Separation. *Mol. Cell* **78,** 236–249.e7 (2020).
2. Tunyasuvunakool, K. *et al.* Highly accurate protein structure prediction for the human proteome. *Nature* **596,** 590–596 (2021).
3. Chittchang, M. *et al.* Poly(L-lysine) as a model drug macromolecule with which to investigate secondary structure and microporous membrane transport, part 2: diffusion studies. *J. Pharm. Pharmacol.* **54,** 1497–1505 (2002).
4. Rubinson, K. A. & Krueger, S. Poly(ethylene glycol)s 2000–8000 in water may be planar: A small-angle neutron scattering (SANS) structure study. *Polymer* **50,** 4852–4858 (2009).
5. Taylor, N. *et al.* Biophysical characterization of organelle-based RNA/protein liquid phases using microfluidics. *Soft Matter* **12,** 9142–9150 (2016).
6. Elbaum-Garfinkle, S. *et al.* The disordered P granule protein LAF-1 drives phase separation into droplets with tunable viscosity and dynamics. *Proc. Natl. Acad. Sci. USA* **112,** 7189–7194 (2015).
7. Wang, H., Kelley, F. M., Milovanovic, D., Schuster, B. S. & Shi, Z. Surface tension and viscosity of protein condensates quantified by micropipette aspiration. *Biophysical Reports* **1,** 100011 (2021).
8. Jawerth, L. *et al.* Protein condensates as aging Maxwell fluids. *Science* **370,** 1317–1323 (2020).
9. Renger, R. *et al.* Co-condensation of proteins with single- and double-stranded DNA. *Proc. Natl. Acad. Sci. USA* **119,** e2107871119 (2022).
10. Benayad, Z., von Bülow, S., Stelzl, L. S. & Hummer, G. Simulation of FUS Protein Condensates with an Adapted Coarse-Grained Model. *J. Chem. Theory Comput.* **17,** 525–537 (2021).
11. Feric, M. *et al.* Coexisting liquid phases underlie nucleolar subcompartments. *Cell* **165,** 1686–1697 (2016).
12. Caragine, C. M., Haley, S. C. & Zidovska, A. Surface fluctuations and coalescence of nucleolar droplets in the human cell nucleus. *Phys. Rev. Lett.* **121,** 148101 (2018).
13. Banani, S. F., Lee, H. O., Hyman, A. A. & Rosen, M. K. Biomolecular condensates: organizers of cellular biochemistry. *Nat. Rev. Mol. Cell Biol.* **18,** 285–298 (2017).
14. Sabari, B. R., Dall'Agnese, A. & Young, R. A. Biomolecular condensates in the nucleus. *Trends Biochem. Sci.* **45,** 961–977 (2020).
15. Espinosa, J. R. *et al.* Liquid network connectivity regulates the stability and composition of biomolecular condensates with many components. *Proc. Natl. Acad. Sci. USA* **117,** 13238–13247 (2020).
16. Li, P. *et al.* Phase transitions in the assembly of multivalent signalling proteins. *Nature* **483,** 336–340 (2012).
17. Bo, S., Hubatsch, L., Bauermann, J., Weber, C. A. & Jülicher, F. Stochastic dynamics of single molecules across phase boundaries. *Phys. Rev. Research* **3,** 043150 (2021).
18. Novikov, D. S., Fieremans, E., Jensen, J. H. & Helpern, J. A. Random walk with barriers. *Nat. Phys.* **7,** 508–514 (2011).
19. Carslaw, H. S. & Jaeger, J. C. *Conduction of Heat in Solids*. (Oxford University Press, 1959).
20. Sprague, B. L., Pego, R. L., Stavreva, D. A. & McNally, J. G. Analysis of binding reactions by fluorescence recovery after photobleaching. *Biophys. J.* **86,** 3473–3495 (2004).
21. Sprague, B. L. *et al.* Analysis of binding at a single spatially localized cluster of binding sites by fluorescence recovery after photobleaching. *Biophys. J.* **91,** 1169–1191 (2006).
22. Mueller, F., Wach, P. & McNally, J. G. Evidence for a common mode of transcription factor interaction with chromatin as revealed by improved quantitative fluorescence recovery after photobleaching. *Biophys. J.* **94,** 3323–3339 (2008).
