# Supplementary material for: Detecting and quantifying liquid–liquid phase separation in living cells by model-free calibrated half-bleaching
Source: Nat Commun. 2022 Dec 16;13:7787. doi: 10.1038/s41467-022-35430-y (PMC9758202; doi:10.1038/s41467-022-35430-y)
Supplement: Supplementary file 2 — Editorial Assessment Report [file 41467_2022_35430_MOESM2_ESM.pdf]

nature portfolio

GUIDED OPEN ACCESS

nature methods | nature communications | communications biology

Contents of this report

1. Manuscript details: overview of your manuscript and the editorial team.
2. Review synthesis: summary of the reviewer reports provided by the editors.
3. Editorial recommendation: personalized evaluation and recommendation from all 3 journals.
4. Annotated reviewer comments: the referee reports with comments from the editors.
5. Open research evaluation: advice for adhering to best reproducibility practices.

About the editorial process

Because you selected the **Nature Portfolio Guided Open Access** option, your manuscript was assessed for suitability in three of our titles publishing high-quality work across the spectrum of methods research: ***Nature Methods***, ***Nature Communications***, and ***Communications Biology***. More information about Guided Open Access can be found here.

Collaborative editorial assessment

Your editorial team discussed the manuscript to determine its suitability for the Nature Portfolio Guided OA pilot. Our assessment of your manuscript takes into account several factors, including whether the work meets the **technical standard** of the Nature Portfolio and whether the findings are of **immediate significance** to the readership of at least one of the participating journals in the Nature Portfolio Guided Open Access methods cluster.

Peer review

Experts were asked to evaluate the following aspects of your manuscript:

- **Novelty** in comparison to prior publications;
- **Likely audience** of researchers in terms of broad fields of study and size;
- **Potential impact** of the study on the immediate or wider research field;
- **Evidence** for the claims and whether additional experiments or analyses could feasibly strengthen the evidence;
- **Methodological detail** and whether the manuscript is reproducible as written;
- Appropriateness of the **literature review**.

Editorial evaluation of reviews

Your editorial team discussed the potential suitability of your manuscript for each of the participating journals. They then discussed the revisions necessary in order for the work to be published, keeping each journal's specific editorial criteria in mind.

Journals in the Nature portfolio will support authors wishing to transfer their reviews and (where reviewers agree) the reviewers' identities to journals outside of Springer Nature.
If you have any questions about review portability, please contact our editorial office at guidedoa@nature.com.

Manuscript details

Tracking number	Submission date	Decision date	Peer review type
GUIDEDOA-21-00321	Nov 25, 2021	Jan 27, 2022	Single-blind

Manuscript title	Author details
MOCHA-FRAP: Detecting and quantifying liquid-liquid phase separation by half-bleaching	Fabian Erdel<br>**Affiliation:** Center for Integrative Biology (CBI), CNRS

Editorial assessment team

**Primary editor**	**Cara Eldridge**<br>Home journal: ***Nature Communications***<br>ORCID: **0000-0001-7001-2312**<br>Email: **cara.eldridge@nature.com**
**Other editors consulted**	**Rita Strack**<br>Home journal: ***Nature Methods***<br>ORCID: **0000-0003-1845-7116**<br><br>**Anam Akhtar**<br>Home journal: ***Communications Biology***<br>ORCID: **0000-0002-8820-8468**
**About your primary editor**	Cara joined Nature Communications in March 2020. After conducting her undergraduate and Master's degrees in Natural Sciences at the University of Cambridge, she went on to complete a PhD at the MRC-Laboratory of Molecular Biology and continued there as a postdoctoral researcher. The focus of her research has been on the effect of replication impediments on stem cell differentiation, and as part of this work she established human embryonic stem cells and induced pluripotent stem cells into the lab. She handles manuscripts in the areas of cellular biotechnology and methods, as well as imaging. Cara is based in the London office.

Editorial assessment and review synthesis

**Editor's summary and assessment**	Condensates can be formed by two different mechanisms, by undergoing interactions with clustered binding sites (ICBS) or by liquid-liquid phase separation (LLPS). Although phenotypic similarities exist, these structures entail differences. Here the authors propose a strategy to characterise the properties of condensates of different origins.
**Editorial synthesis of reviewer reports**	The reviewers felt that this manuscript covered an important topic and that the presented method has the potential to be very useful to researchers in the LLPS field.<br><br>Reviewer #1 had concerns on whether this could be applied to other in vitro and in vivo systems, and whether this could be used as a stand-alone method.<br><br>Reviewer #2 thought that the design was innovative but didn't feel that the experimental details were such that this could be easily repeated by other labs. A big concern was the qualitative nature of the detection method and the size of puncta needed for analysis.<br><br>Reviewer #3 felt that the problem being addressed was of high interest to the community, but had technical concerns about the experiments and their interpretation. They also felt that there was a lack of novelty compared to some existing methods.

Editorial recommendation

Nature Methods<br><br>Revision not invited	We share the concerns about novelty and general applicability of the approach with the referees. We think these issues, along with the technical concerns, are sufficient to preclude further consideration in Nature Methods.
Nature Communications<br><br>Major revisions with extension of the work	For resubmission to Nature Communications, we would require all of the reviewers' comments to be addressed in full. We would require you to show and discuss the novelty in your method over the references mentioned by Reviewer #1 and Reviewer #3. We would also require additional benchmarking as requested by Reviewer #1, and application of the method to more LLPS systems as suggested by Reviewer #1 and #2.
Communications Biology<br><br>Major revisions	For consideration at Communications Biology, we would also require you to address all reviewers' comments. However, we are willing to forego Reviewer #1 and Reviewer #2's request to characterise more LLPS systems with this method.

Next steps

**Editorial recommendation 1:**	Our top recommendation is to revise and resubmit your manuscript to *Nature Communications.* We feel the additional experiments required require a major revision with some extension of the work.
**Editorial recommendation 2:**	You may also choose to revise and resubmit your manuscript to *Communications Biology.* This option might be best if the requested experimental revisions are not feasible at this time.
**Note**	As stated on the previous page *Nature Methods* is not inviting a revision at this time. Please keep in mind that the journal will not be able to consider any appeals of their decision through Guided Open Access.

Revision

To follow our recommendation, please upload the revised manuscript files using **the link provided in the decision letter**. Should you need assistance with our manuscript tracking system, please contact Adam Lipkin, our Nature Portfolio Guided OA support specialist, at guidedOA@nature.com.

Revision checklist

- ☐ Cover letter, stating to which journal you are submitting
- ☐ Revised manuscript
- ☐ Point-by-point response to reviews
- ☐ Updated Reporting Summary and Editorial Policy Checklist
- ☐ Supplementary materials (if applicable)

Submission elsewhere

If you choose not to follow our recommendations, you can still take the reviewer reports with you.

Option 1: Transfer to another Nature Portfolio journal
Springer Nature provides authors with the ability to transfer a manuscript within the Nature Portfolio, without the author having to upload the manuscript data again. To use this service, **please follow the transfer link provided in the decision letter.** If no link was provided, please contact guidedOA@nature.com.

> *Note that any decision to opt in to In Review at the original journal is not sent to the receiving journal on transfer. You can opt in to In Review at receiving journals that support this service by choosing to modify your manuscript on transfer.*

Option 2: Portable Peer Review option for submission to a journal outside of Nature Portfolio
If you choose to submit your revised manuscript to a journal at another publisher, we can share the reviews with another journal outside of the Nature Portfolio if requested. You will need to request that the receiving journal office contacts us at guidedOA@nature.com. We have included editorial guidance below in the reviewer reports and open research evaluation to aid in revising the manuscript for publication elsewhere.

Annotated reviewer reports

The editors have included some additional comments on specific points raised by the reviewers below, to clarify requirements for publication in the recommended journal(s). However, please note that all points should be addressed in a revision, even if an editor has not specifically commented on them.

Reviewer #1 information

**Expertise**	Biophysics, LLPS and methods to study, fluorescence tools
**Editor's comments**	This reviewer found your work to be on an important topic, given uncertainty in classification methods in the field. Their main concerns were whether this method could be applied to other in vitro and in vivo systems, and whether this method could be used alone to distinguish between LLPS and ICBS.

Reviewer #1 comments

Section	Annotated Reviewer Comments
**Remarks to the Author: Overall significance**	The authors present a method, MOCHA-FRAP, to distinguish liquid condensates from clusters formed via other mechanisms, specifically ICBS. This is an important topic given the uncertainty in the field as to the specific requirements to classify a condensate as liquid-like. First, they develop clever model systems for LLPS and ICBS, one of each both in vitro and in vivo (mouse fibroblasts). Using these well-defined systems, the authors show that 1,6-hexandiol treatment and standard FRAP (full or partial) are not capable of distinguishing between LLPS and ICBS. Rather, they show that a substantial decrease in fluorescence intensity (quantified as dip depth) in the un-bleached half of a half-FRAP experiment is a signature of a liquid droplet while it does not occur for ICBS clusters. Thus, they present a straightforward and model-free method to distinguish LLPS from ICBS.
**Remarks to the Author: Strength of the claims**	There are two main issues that the authors should consider. First, although this is a simple way to distinguish between the two modes (LLPS vs. ICBS), it is not clear if this will apply to other in vitro and in vivo situations. Does this distinction apply to other LLPS or ICBS studies from other labs performed in different systems? Second, related to the first, can this be a stand-alone method to distinguish between the two modes? It will be much more convincing to demonstrate the distinguishing feature(s) of the two modes by orthogonal means that probe the microrheological property such as fusion, diffusion via single particle tracking etc.<br><br>**Distinguishing between two modes, as requested here, will not be a mandatory requirement for consideration at *Communications Biology*. This is required for *Nature Communications*.**

	Below are additional comments.<br><br>Comments:<br>1. This experiment has been published previously by the corresponding author (Erdel, 2020), which may decrease the novelty and impact, but the comparison to other methods and quantification are new to this study.<br>**For both the journals, the novelty of your method presented here must be explained over this existing publication.**<br>2. Fusion of droplets is another common test of liquid-like condensates; the authors should consider testing this in their benchmark as well.<br>**For both journals, we would require you to add in benchmarking experiments on the fusion of droplets.**<br>3. Can the authors discuss how the cutoff between ICBS and LLPS was determined?<br>**Both journals would require more information on the distinction between the two here.**<br>Also, it is interesting to consider what is occurring molecularly at this boundary: is there a switch between non-liquid and liquid or is it a gradient as implied by Fig 2i?<br>**Kindly address this.**<br>This is probably outside the scope of this study, but in the PLL-HA in vitro system, can the extent of cross-linking be tuned to probe the line between LLPS and ICBS?<br>**It would be interesting if you were able to address this, but we do not require it for *Nature Communications* or *Communications Biology*.**<br>Minor:<br>• Line 87, “halve” should be “half”.
**Remarks to the Author: Reproducibility**	Statistical significance is unclear. For example, there are no statements about how many times the same experiments were conducted or how many sets of result (for example, FRAP) were combined to generate the curve with error bars.<br>**For both journals, full details on the statistics are required.**

Reviewer #2 information

**Expertise**	Biophysics and single molecule imaging, LLPS, FRAP
**Editor’s comments**	The reviewer also feels that the manuscript looks to address an important issue in the field and is an interesting contribution. However, they were concerned that the practical details here are missing and it would be hard to implement this method.

Reviewer #2 comments

**Section**	**Annotated Reviewer Comments**

**Remarks to the Author: Overall significance**	In this manuscript by Muzzopappa et al., the authors characterize preferential internal mixing as a robust signature of liquid-liquid phase separation (LLPS) and suggest that detecting this signature by FRAP (MOCHA-FRAP) is a non-invasive method to identify LLPS. This work aims to address an important question in the field of LLPS - how to differentiate puncta formed by LLPS in living cells from those formed by other mechanisms, e.g., proteins interacting with clustered binding sites (ICBS). This work is certainly of interest to the LLPS field.
**Remarks to the Author: Impact**	The reviewer finds some of the experimental designs are innovative, e.g., 1) using Poly-L-Lysine (PLL) interacting with chemically cross-linked coacervates composed of PLL and Hyaluronic Acid (HA) to mimic ICBS, and 2) doing half-FRAP and measuring the dip depth of the non-bleached half to determine preferential internal mixing. However, because important details of the new method are missing, it is practically difficult for readers to implement this method to detect LLPS. Also, whereas the field's difficulties in determining LLPS happens in live cells and especially for small puncta with sizes near diffraction limits (Mcswiggen DT et al, Genes Dev 33, 1619-1634 (2009)), MOCHA-FRAP is benchmarked in vitro, demonstrated for only one specific LLPS system (DDX4) in live cells, and is only applicable to large enough puncta as stated by the authors. Overall, the paper influences thinking in the field of LLPS, but the above described facts limit the impact of this work.
**Remarks to the Author: Strength of the claims**	Detailed comments:<br>1. The method the authors propose to detect LLPS is qualitative ("large" instead of "small" dip depth after half-FRAP) instead of quantitative, making it difficult for readers to implement the method to determine whether a punctum is formed by LLPS in their own systems. How large a dip depth is considered characteristic of LLPS? Since the internal mixing rate compared with external mixing rate is dependent on the protein of interest and environmental parameters it is necessary to characterize more published LLPS systems, e.g., the FET family proteins, to quantify the characteristic dip depth of LLPS than what the authors have done. Given the goal of this work, characterizing more live-cell LLPS systems is especially important. Multivalent interactions that drive LLPS in live cells have different dynamics depending on the scaffold protein (Chong S et al, Science 361, eaar2555 (2018)), which can cause differences in the external mixing rates and how they compare with internal mixing rates.<br>**For *Nature Communications* we would require you to characterise more LLPS systems to address this concern. We would also require more information on how you assign LLPS from your qualitative data. For *Communications Biology*, we do not mandate using more LLPS systems, but we do ask you to give more details on LLPS assignment with the data, as raised by the reviewer.**<br>2. As the authors have discussed, MOCHA-FRAP can only be used for detecting LLPS within large enough puncta. How large do puncta need to be for this

	method to be applicable? It seems to the reviewer that the limitation of sizes comes from the fact that a punctum needs to be at least in the scale of microns so that a half of the punctum can be precisely portrayed on a fluorescence microscope in preparation of FRAP. Such information needs to be provided in the paper as readers need it to decide whether the method is applicable to their systems.<br>**For *Nature Communications* we require the full information on the puncta size required, and all experimental details needed for these experiments to be repeated. If the manuscript were to be accepted, we would also require deposition of the full method to the protocol exchange. For *Communications Biology*, please address this comment textually as completely as possible.**<br>3. A live-cell image of chromodomain (CD) of CBX1/HP1beta is needed. Currently there is only FRAP data of one punctum of CD (Fig. 2h).<br>**We would require this for both the journals.**<br>4. It is unclear how a sigmoidal relationship between dip depth and interfacial energy per molecule (Fig. 2i) is determined.<br>**Please add this information for both the journals.**

Reviewer #3 information

**Expertise**	Biophysics, FRAP, phase separation
**Editor's comments**	This reviewer finds that the problem tackled here is an important one, but they had some concerns about the premise of these experiments, as well as the interpretation.

Reviewer #3 comments

Section	Annotated Reviewer Comments
**Remarks to the Author: Overall significance**	The work of Muzzopappa et al. focuses on demonstrating a new method for detecting and quantifying liquid-liquid phase separation. The proposed approach focuses on the use of FRAP and, more precisely, in half bleaching the condensate and studying the different response of the two halves. The authors benchmarked the approach using synthetic polymers and established phase-separation constructs, both in vitro and in living cells. However, while overall the data presented are reasonable and interesting, the premise of the experiments and their interpretation raise some concerns that I described in the following sections.
**Remarks to the**	The problem tackled by the authors is of high relevance, because identifying

**Author: Impact**	the nature of puncta in cells and distinguishing condensates from aggregates or large complexes is a current challenge in the field. In the same spirit, the use of quantitative FRAP experiments that go beyond simply testing for presence or absence of recovery in biomolecular condensates is very welcome.
**Remarks to the Author: Strength of the claims**	While overall the data presented are reasonable and interesting, the premise of the experiments and their interpretation raise some concerns that I have attempted to detail in the following.<br><br>Major concerns:<br>- The authors refer to liquid-liquid phase-separation (LLPS) and interactions with clustering binding sites (ICBS) such as the case of chromatin. While the first term (LLPS) is abundantly used in literature, I could not find any usage of the terminology of ICBS. I think it is important for the authors to provide a very precise definition of the two distinct phenomena that they want to capture. The terminology of LLPS invokes a liquid nature and a phase separation process. However, as discussed in recent reviews (e.g. ref 8), LLPS can give rise to fluid, gel, or even solid-like condensates. This largely reflects the nature of the interactions at play across the different molecules and does not necessary invoke clustering of binding sites. LLPS can occur even for a single polymer mixture and its fluidity and recovery will depend on the number and type of interactions. Since LLPS can also evolve over time, this can further complicate the interpretation of the nature of the puncta. A better clarification of the terms is essential to understand which phenomenon is being described and where the threshold is traced between LLPS and ICBS.<br>**For both journals, we would require a thorough explanation of these two terms and the differences between them, highlighting the significance of your method.**<br>- The idea that the recovery time in FRAP largely depends on viscosity of the solution, area that is photobleached as well as the existence of long-lived contacts is largely discussed in ref. 16, where photobleaching of different areas is proposed as a methodology to verify whether the system is under a diffusion limited control or a kinetic/interaction control. The authors may want to explicit this point and discuss the differences between the approaches<br>**For both the journals, we would need you to show the novelty of your method, and highlight the differences compared to this approach outlined by the reviewer here.**<br>- The most interesting result of the manuscript is the link between dip-depth and the interfacial interaction per molecule. Some additional points along the curve with different systems from the ones shown may solidify the point that there is a master curve controlling the link between dip-depth and interfacial interaction. In this respect the simulation may provide some further insights. However, the simulation procedure used to produce Supplementary Fig. 8 is not describe, few details are reported in the figure caption, and it is not clear whether one can extract a simulated trend that would support the empirical finding (perhaps panel c in Supplementary Fig. 8).

	**Both journals require a thorough description of the simulation, and the extraction of a simulated trend.**<br>- The model that produces the theoretical predictions in Supplementary Fig. 7 is not described.<br>**Both journals require a thorough explanation of your model.**
**Remarks to the Author: Reproducibility**	The experiments seem reproducible.<br>However no description is provided for the theoretical model and simulation procedure, which makes difficult to evaluate the work and reproduce it.<br>**Both journals would need a full description of this.**<br><br>The data availability statement states that "no datasets were generated or analyzed during the current study", which does not seem accurate.<br>**As stated in the Open Research Evaluation section, on resubmission we would need a full Source Data file.**<br><br>The code availability statement states that no custom code were used, but simulation have been performed and there is no reference to the software or the code that has been used.<br>**As stated in the Open Research Evaluation section, on resubmission we would need the custom code to be accessible, and ideally uploaded to github.**

Open research evaluation

General information

Guidelines for Transparency and Openness Promotion (TOP) in Journal Policies and Practices ("TOP Guidelines")

The recommendations and requests in the table below are aimed at bringing your manuscript in line with common community standards as exemplified by the TOP Guidelines. While every publisher and journal will implement these guidelines differently, the recommendations below are all consistent with the policies at Nature Portfolio. In most cases, these will align with TOP Guidelines Level 2.

FAIR Principles

The goal of the recommendations in the table below related to **data or code** availability is to promote the FAIR Guiding Principles for scientific data management and stewardship (*Scientific Data* **3**: 160018, 2016). The FAIR Principles are a set of guidelines for improving 4 important aspects of digital research objects: **F**indability, **A**ccessibility, **I**nteroperability and **R**eusability.

ORCID

ORCID is a non-profit organization that provides researchers with a unique digital identifier. These identifiers can be used by editors, funding agencies, publishers, and institutions to reliably identify individuals in the same way that ISBNs and DOIs identify books and articles. Thus the risk of confusing your identity with another researcher with the same name is eliminated. **The ORCID website** provides researchers with a page where your comprehensive research activity can be stored.

Springer Nature collaborates with the ORCID organization to ensure that your research contributions (as authors and peer reviewers) are correctly attributed to you. Learn more at https://www.springernature.com/gp/researchers/orcid

Data availability
**Data Availability Statement**
Thank you for including a Data Availability statement. While you have included some important information, the editors have noted that some details appear to be missing. The Data Availability Statement should be as detailed as possible and include accession codes or other unique IDs for deposited data, information about where source data can be found, and specify any restrictions to data access that may apply. At a minimum, the statement should indicate that data are available upon request and explain how data access can be granted. If data access is not possible, the reasons for this must be made clear in the Data Availability Statement.<br><br>More information about the Nature Portfolio data availability policy can be found here: https://www.nature.com/nature-portfolio/editorial-policies/reporting-standards#availability-of-data
**Other data requests**
We strongly encourage the deposition of your full microscopy image data sets in the Image Data Resource: https://idr.openmicroscopy.org/about
All source data underlying the graphs and charts presented in the main figures must be made available as Supplementary Data (in Excel or text format) or via a generalist repository (eg, Figshare or Dryad). This is mandatory for publication in a Nature Portfolio journal, but is also best practice for publication in any venue.<br><br>The following figures require associated source data: Fig.1e-i, Fig 2a-j, FigS3, Fig.S8, Fig.S9, Fig.S11.
**Data publishing recommendations**
Please note that the legend for figure 1e is incorrectly labelled as ‘1d’. Please rectify this in the figure legend.
**Code availability and citation**

Thank you for including a Code Availability statement in
your manuscript. However, we noted that you have only indicated that custom code are available upon request. To adhere to community standards and promote transparency in research, the Code Availability Statement must indicate whether and how the code or algorithm can be accessed, including any restrictions to access. Public release of custom software may be required for publication in a Nature Portfolio journal.

Upon publication, Nature Portfolio journals consider it best practice to release custom computer code in a way that allows readers to repeat the published results. Code should be deposited in a DOI-minting repository such as Zenodo, Gigantum or Code Ocean and cited in the reference list following the guidelines described in our policy pages (see link below). Authors are encouraged to manage subsequent code versions and to use a license approved by the open source initiative. Full details about how the code can be accessed and any restrictions must be described in the Code Availability statement.

See here for more information about Nature Portfolio's code availability policies:
https://www.nature.com/nature-portfolio/editorial-policies/reporting-standards#availability-of-computer-code

We also provide a Code and Software submission checklist that you may find useful:
https://www.nature.com/documents/nr-software-policy.pdf

Please note: because of advanced features used in this form, you must use Adobe Reader to open the document and complete it.

Reporting & reproducibility

We encourage you to share your step-by-step experimental protocols on a protocol sharing platform of their choice. The Nature Portfolio’s Protocol Exchange is a free-to-use and open resource for protocols; protocols deposited in Protocol Exchange are citable and can be linked from the published article. More details can be found at
www.nature.com/protocolexchange/about

Please state in the legends how many times each
experiment was repeated independently with similar results. This is needed for all experiments, but is particularly important wherever results from representative experiments (such as micrographs) are shown. If space in the legends is limiting, this information can be included in a section titled "Statistics and Reproducibility" in the methods section.

Legends requiring revision:

Please note that this information is missing in the legends of figure 1c and supplementary figures 1a-j.

Materials availability

We recommend that you deposit your newly generated plasmids in a community repository, such as https://www.addgene.org/ , to support open research efforts.

Statistical reporting

Wherever statistics have been derived (e.g. error bars, box plots, statistical significance) the legend needs to provide and define the n number (i.e. the sample size used to derive statistics) as a precise value (not a range), using the wording "n=X biologically independent samples/animals/cells/independent experiments/n= X cells examined over Y independent experiments" etc. as applicable.

Legends requiring revision:

1. Please note that this information is missing in the legends of figures 1e, f-i; 2g-i and supplementary figures 6; 9c-i; 11a, b.

Statistics such as error bars, significance and p values cannot be derived from n<3 and must be removed from all such cases.

We strongly discourage deriving statistics from technical replicates, unless there is a clear scientific justification for why providing this information is important. Conflating technical and biological variability, e.g., by pooling technically replicates samples across independent experiments is strongly discouraged. (For examples of expected description of statistics in figure legends, please see the following https://www.nature.com/articles/s41467-019-11636-5 or https://www.nature.com/articles/s41467-019-11510-4).

All error bars need to be defined in the legends (e.g. SD, SEM) together with a measure of centre (e.g. mean, median). For example, the legends should state something along the lines of "Data are presented as mean values +/- SEM" as appropriate.

All box plots need to be defined in the legends in terms of<br>minima, maxima, centre, bounds of box and whiskers and percentile.<br><br>**<u>Legends requiring revision:</u>**<br><br>1. Please note that the error bars need to be defined in the legends of supplementary figures 9c-i.<br>2. Please note that the measure of centre for the error bars needs to be defined in the legends of figures 1e; 2j.<br>3. If the shaded areas denote error bands then the error bands need to be defined in the legends of figures 2a-c.<br>4. Please note that the box plots need to be defined in terms of minima, maxima, centre, bounds of box and whiskers and percentile in the legends of supplementary figures 11a, b.<br><br>The figure legends must indicate the statistical test used. Where appropriate, please indicate in the figure legends whether the statistical tests were one-sided or two-sided and whether adjustments were made for multiple comparisons.<br>For null hypothesis testing, please indicate the test statistic (e.g. F, t, r) with confidence intervals, effect sizes, degrees of freedom and P values noted.<br>Please provide the test results (e.g. P values) as exact values whenever possible and with confidence intervals noted.
**Data presentation**
Bar graphs should only be used to present counts or proportions. If you are using bar graphs that present means/averages, it is best practice to include individual data points and/or convert the graph to a boxplot or dot-plot. You may wish to refer to this blog post (https://ecrlife420999811.wordpress.com/2018/07/10/beyond-bar-graphs-free-tools-and-resources-for-creating-more-transparent-figures-for-small-datasets/) about representing data distribution in plots (particularly for small datasets).
Please state in the figure legends how many times each experiment was repeated independently with similar results. This is needed for all experiments, but is particularly important wherever results from representative experiments (such as micrographs) are shown. If space in the legends is limiting, this information can be included in a section titled “Statistics and Reproducibility” in the methods section.
Please ensure that data presented in a plot, chart or other visual representation format shows data distribution clearly (e.g. dot plots, box-and-whisker plots). When using bar charts, please overlay the corresponding data points (as dot plots) whenever possible and always for n ≤ 10. (Please see the following editorial for the rationale behind this request and an example https://www.nature.com/articles/s41551-017-0079).

**Panels requiring revision:**<br>Please note that data presentation has to be revised to comply with our policy in figures 1e; 2j and supplementary figure 3b.
Please ensure that all micrographs include a scale bar and this scale bar is defined on the panels or in the figure legends.
